# Sample size matters when estimating test–retest reliability of behaviour

**DOI:** 10.3758/s13428-025-02599-1

**Published:** 2025-03-21

**Authors:** Brendan Williams, Lily FitzGibbon, Daniel Brady, Anastasia Christakou

**Affiliations:** 1 Centre for Integrative Neuroscience and Neurodynamics, University of Reading, Harry Pitt Building, Reading, UK; 2 School of Psychology and Clinical Language Sciences, University of Reading, Reading, UK; 3 Division of Psychology, Faculty of Natural Sciences, University of Stirling, Stirling, UK; 4 Department of Computer Science, Faculty of Engineering, University of Sheffield, Sheffield, UK

**Keywords:** Reliability, Test retest, Sample size, Reinforcement learning, Computational modelling, Reversal learning, Cognitive flexibility

## Abstract

Intraclass correlation coefficients (ICCs) are a commonly used metric in test–retest reliability research to assess a measure’s ability to quantify systematic between-subject differences. However, estimates of between-subject differences are also influenced by factors including within-subject variability, random errors, and measurement bias. Here, we use data collected from a large online sample (*N* = 150) to (1) quantify test–retest reliability of behavioural and computational measures of reversal learning using ICCs, and (2) use our dataset as the basis for a simulation study investigating the effects of sample size on variance component estimation and the association between estimates of variance components and ICC measures. In line with previously published work, we find reliable behavioural and computational measures of reversal learning, a commonly used assay of behavioural flexibility. Reliable estimates of between-subject, within-subject (across-session), and error variance components for behavioural and computational measures (with ± .05 precision and 80% confidence) required sample sizes ranging from 10 to over 300 (behavioural median *N*: between-subject = 167, within-subject = 34, error = 103; computational median *N*: between-subject = 68, within-subject = 20, error = 45). These sample sizes exceed those often used in reliability studies, suggesting that sample sizes larger than are commonly used for reliability studies (circa 30) are required to robustly estimate reliability of task performance measures. Additionally, we found that ICC estimates showed highly positive and highly negative correlations with between-subject and error variance components, respectively, as might be expected, which remained relatively stable across sample sizes. However, ICC estimates were weakly or not correlated with within-subject variance, providing evidence for the importance of variance decomposition for reliability studies.

**Supplementary Information:**

The online version contains supplementary material available at 10.3758/s13428-025-02599-1.

## Introduction

The study of learning and decision-making processes in psychology and neuroscience research typically relies on the use of conditioning tasks to assay behaviour. These include instrumental conditioning tasks where subjects learn associations between actions and outcomes, such as pulling a slot machine lever and winning money, through experience. These associations either increase or decrease the likelihood of a given action being made in the future, depending on whether the outcome was rewarding or punishing. However, these associations also need to be updated flexibly for an individual to respond adaptively to dynamic and changing environments. Supporting this flexible updating of action–outcome associations relies on cognitive flexibility. Cognitive flexibility, broadly defined, is a complex competence that enables the maintenance of a given goal (such as winning money) while appropriately updating the required actions to achieve that goal (Dajani & Uddin, 2015), by identifying, selecting, and executing the optimal response strategy (Yu et al., 2019).

A commonly used measure of cognitive flexibility is the reversal learning task (Izquierdo et al., 2017). In this task, actions are associated with differing probabilities of rewards and/or punishments. These varying probabilities mean that different actions will, on average, have outcomes that make them more or less favourable than other actions. During the task, the probabilities associated with the available actions change such that previously favourable actions become less favourable, and vice versa. When this occurs, a cognitively flexible agent shifts action selection towards the newly favourable and away from the previously favourable outcomes to maintain goal-directed behaviour (assuming the agent’s goal is to maximise gains). Performance in the reversal learning task can be indexed using summary measures of behaviour, such as choice accuracy, reaction time, and the pattern of win–stay/lose–shift behaviour, or by deriving latent descriptors of performance using computational modelling. For these latter indices, reinforcement learning models can be fitted to choice behaviour to estimate parameters that describe features of behaviour, such as the rate of learning, or to what degree behaviour is driven by estimates of expected value.

The reliability of reversal learning behaviour has been previously reported in several papers using behavioural and computational modelling (Schaaf et al., 2023; Waltmann et al., 2022), in schizophrenia (Reddy et al., 2016), and using functional magnetic resonance imaging (Freyer et al., 2009). At the group level, reversal learning performance has been shown to produce reliable activation in regions of prefrontal and parietal cortices and the cingulate gyrus (Freyer et al., 2009), and reliable behavioural effects in both individuals with schizophrenia (Reddy et al., 2016) and healthy non-clinical populations (Schaaf et al., 2023; Waltmann et al., 2022). However, there appears to be less agreement in the current literature about the reliability of parameters derived using computational modelling.

Two recent studies are particularly pertinent to this issue, both exploring the test–retest reliability of computational model parameters, but drawing somewhat different conclusions. Waltmann et al. (2022) assessed the reliability of behavioural and computationally derived measures of performance during reversal learning. They used several approaches, including the calculation of intraclass correlation coefficients (ICCs), split-half reliability, variance decomposition, and simulation work. Behavioural measures of performance (e.g., reaction time, accuracy, stay behaviour, and perseveration) had good to excellent reliability (based on ICC and correlation coefficients). Waltmann et al. (2022) also used variance decomposition to demonstrate that some behavioural measures (such as accuracy and staying after losses) had high between-subject variability while others (such as reaction time measures) had high within-subject variability. Similarly, parameter estimates from the best-fitting computational model were found to have good to excellent reliability. Schaaf et al. (2023) assessed the reliability of behaviour and computational measures of performance during both a reversal learning and a two-armed bandit task. Using the same ICC coefficient interpretations as Waltmann et al. (2022), Schaaf et al. (2023) found fair reliability for accuracy and lose–shift behaviour and good reliability for win–stay behaviour in the two-armed bandit task. Additionally, Schaaf et al. (2023) found good reliability for accuracy and lose–shift behaviour and excellent reliability for win–stay behaviour in the reversal learning task. Schaaf et al. (2023) also found that parameter estimates showed good identifiability (meaning equivalent likelihoods cannot be produced by different sets of parameter estimates during fitting (Gershman, 2016)) for simulated behaviour but not for human subject data. Thus, this suggests that although computational models can recover parameters reliably when behaviour is truly stable (i.e., generated by an artificial agent), they struggle with behaviour from real subjects, which is variable and influenced by context. Indeed, Schaaf et al. (2023) also demonstrate that momentary mood can influence model parameters estimated from behaviour, with happiness and stress being associated with decreased and increased sensitivity to negative feedback, respectively, in the two-armed bandit task.

ICCs are widely used in reliability studies as an indicator of a method’s ability to measure systematic differences between subjects. However, a method’s ability to capture systematic differences between subjects is influenced by factors including within-subject variability, random errors, and measurement bias (Liljequist et al., 2019). Generally, an ICC is calculated by taking the ratio of between-subject variance and the total amount of variance for a given measure (McGraw & Wong, 1996). However, because an ICC is a ratio, the individual contributions of variance components to the overall coefficient cannot be accounted for. A second limitation for only using ICCs to determine reliability is that ICC calculations are more heavily influenced by increases in between-subject variance than session variance effects (Barnhart et al., 2007, 2016; Gorgolewski et al., 2013). Therefore, stable between-subject effects over time are essential to minimise disproportionate biasing of ICC estimates. Variance decomposition, by contrast, keeps the variance of a given measure in its composite parts; in the case of ICC(A,1), this includes measures of within-subject session variance, between-subject variance, and error variance.

Waltmann et al. (2022) used variance decomposition to demonstrate that some behavioural measures (such as accuracy and staying after losses) and some computational model parameters (learning rate) had high between-subject variability, while others (such as reaction time measures and reinforcement sensitivities for wins and losses) had high within-subject variability. Schaaf et al. (2023) did not use variance decomposition to assess the reliability of their computational modelling parameters, but they measured subjects’ mood at each time point and found some evidence that changes in mood can explain within-subject variability in some model parameters.

Important﻿ factors to consider when assessing the reliability of a variable are the precision and accuracy of the metric used to quantify its variability. One factor that influences precision and accuracy is sample size, with smaller sample sizes generally producing less precise estimates of reliability (Chen et al., 2023; Clarke & Wheaton, 2007; Maas & Hox, 2004, 2005; Neubauer et al., 2020). Previous work has also suggested that the precision of variance component estimates can be improved by increasing sample size (Paccagnella, 2011), and increasing sample sizes can reduce bias in estimates of reliability measures within subjects (Neubauer et al., 2020). Here, we present results on the reliability of reversal learning using a sample size larger than in previous reports (Freyer et al., 2009; Reddy et al., 2016; Schaaf et al., 2023; Waltmann et al., 2022).

To complement previous research, we firstly replicate the analytical pipeline of Waltmann et al. (2022) using data from a large sample of subjects (*N* = 150) on a similar reversal learning task. We then build upon previous research by investigating the effect of sample size on estimates of reliability using synthetic datasets based on the statistical properties of the collected “ground truth” data. To pre-empt our results, our replication analysis showed that behavioural variables and computational modelling parameters had similar reliability patterns to Waltmann et al. (2022) as assessed using ICCs. We then used simulation to investigate the effects of sample size on estimates of variance components, and the association between these variance components and ICC values. We generated synthetic datasets based on the underlying distributions and associations between sessions from our collected data. We generated 1000 synthetic datasets with a sample size of 300 for each task performance measure. For each synthetic dataset we calculated estimates of variance components and ICC measures at each sample size from 10 to 300. We then determined at what sample sizes the proportions of variance components stabilised in our synthetic dataset, based on our ground truth data. Our results show that the critical sample size for stable estimates of variance components for our task performance measures change as a function of the level of precision (half-width) and confidence (*point of stability* percentile). Stable estimates of variance components required sample sizes ranging from 10 to over 300 participants (behavioural median *N*: between subjects = 167, within subjects = 34, error = 103; computational median *N*: between subjects = 68, within subjects = 20, error = 45) with ± 0.05 precision and 80% confidence, which exceeds sample sizes typically observed in test–retest research. Thus, our results suggest that a larger than usual sample size (and/or data density) is required to accurately estimate variance components and, in turn, infer reliability.

## Methods

### Subjects

The Prolific online recruitment platform (https://www.prolific.co/) was used to enrol eligible subjects (filters described in supplementary materials) in this study over two waves (wave 1: August–September 2021; wave 2: March–April 2022). A total of 251 subjects completed the experiment in the first part of the study. After the first phase, two subjects were excluded because they failed instructional attention check questions, and 14 were excluded because they failed nonsensical attention check questions or careless/insufficient effort (C/IE) responding checks (further described below). A total of 222 subjects completed the second part of the experiment. After the second phase, one subject was excluded because they failed instructional attention check questions, and eight were excluded because they failed nonsensical attention check questions or the C/IE responding checks. The mean interval between the two sessions was 12.53 days (*SD* = 1.89, range = 11.45–22.25 days). Lastly, we used a binomial test to identify 58 subjects performing at or below chance level in the reversal learning task in at least one session, and excluded them from further analyses (Zorowitz et al., 2023). Our final sample used for statistical analysis included 150 subjects (mean age = 35.450, *SD* = 13.30, range = 19–73, female = 97), and demographic information for these participants is summarised in Table 1.

**Table 1:** Demographic information for the participants included in this study

	Mean	*SD*	Min	Max
Age	35.45	13.30	19	73
Prolific approvals	511.27	442.50	32	2672
	Range	Frequency		
Age	19–25	43		
	26–39	59		
	40–59	39		
	60–75	8		
	NA	1		
Sex	Male	53		
	Female	97		
Student	No	106		
	Yes	32		
	NA	12		
Prolific approvals	0–100	15		
	101–200	20		
	201–300	24		
	301–400	17		
	401–500	18		
	501–750	23		
	751–1000	16		
	1001–1500	11		
	1501–2000	4		
	2001–2500	0		
	2501–3000	2		
Prolific rejections	0	57		
	1	42		
	3	13		
	2	25		
	4	8		
	6	2		
	5	3		
Country of residence	United Kingdom	143		
	Portugal	3		
	Greece	3		
	Poland	1		
Employment status	Full-time	74		
	Not in paid work	13		
	Other	10		
	Part-time	28		
	Unemployed (and job-seeking)	10		
	Due to start a new job within the next month	2		
	NA	13		

Subjects who successfully completed the first phase of the study were reimbursed £1.75 for their time, framed as a basic pay rate of £1.25 plus a 50p bonus based on their performance in the reversal learning task. This was done to maximise the likelihood that subjects would remain focused while completing the task. Subjects who successfully completed the second phase of the study were reimbursed £2.50 for their time, framed as a basic rate of £1.25 plus a performance bonus of £1.25. This payment bonus was larger during the second phase of the study than the first to encourage subjects to complete both parts of the study. This study was approved by the research ethics committee of the University of Reading (2021–50-AC).

### Reversal learning task

#### Task overview

**Fig. 1:**
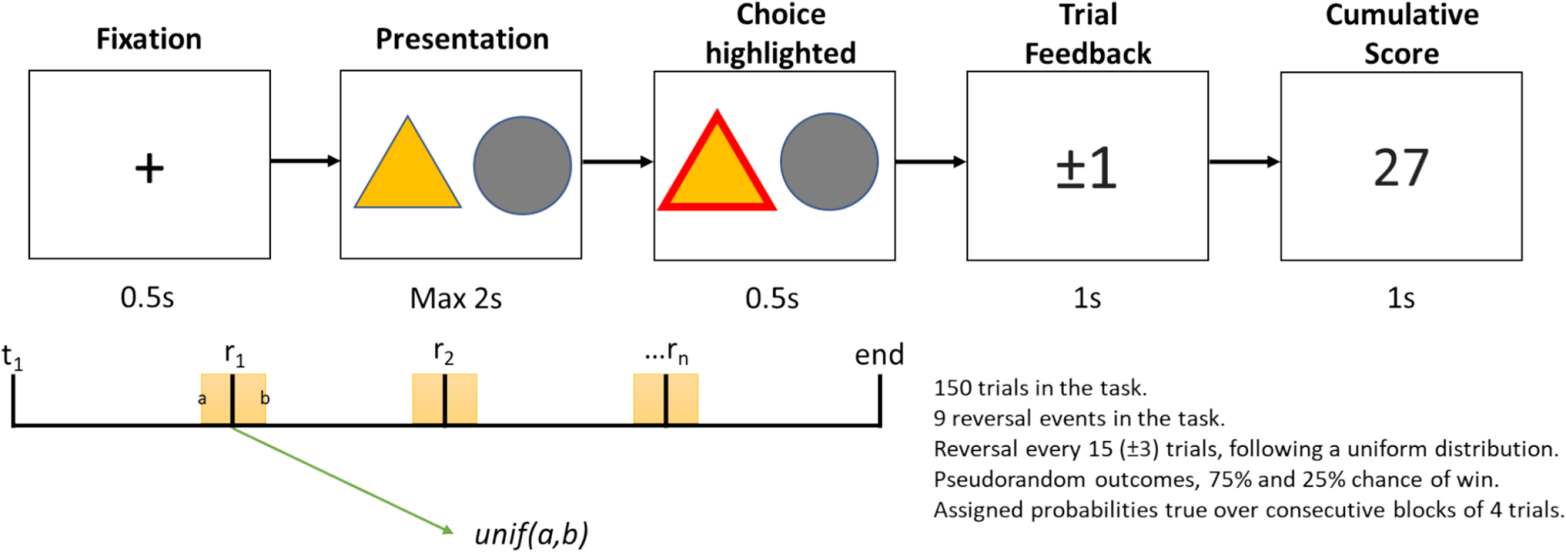
Reversal learning task overview. Subjects completed 150 trials. On each trial they were presented with two abstract stimuli, one of which was associated with reward on 75% of trials, and the other on 25% of trials (true over consecutive blocks of four trials). After subjects made their choice, they received either reward (+ 1 point) or punishment (− 1 point). The assigned reward probabilities reversed nine times during the task, and this reversal occurred every 15 ± up to 3 trials, based on a uniform distribution

Subjects were presented with two visually distinguishable abstract stimuli that would appear randomly on screen, one left of centre, and one right of centre. Subjects selected one of these stimuli by pressing the ipsilateral arrow key on their keyboard. Subjects were given up to two seconds to make a valid choice response. After stimulus selection, subjects were presented with the outcome of their choice. Choice outcomes were either the gain or loss of a single point. Initially, one stimulus was randomly assigned as the “correct” stimulus. Selection of the correct stimulus meant subjects had a 75% chance of gaining a point and 25% chance of losing a point. The “incorrect” stimulus had the inverse probabilities for gains and losses. Outcomes for the correct and incorrect stimuli were pseudorandomised, so the assigned outcome probabilities were true over contiguous blocks of four trials. Subjects experienced nine reversals during the task, with each reversal occurring every 15 ± up to 3 trials (uniform distribution). At the point of reversal, the identity of the correct stimulus was changed, such that the correct stimulus became the incorrect stimulus and vice versa. If subjects did not make a valid choice response within the two-second time limit, then they were told they were too slow, and lost a single point. Subjects completed 150 trials of the reversal learning task. This task was created using the JavaScript library jsPsych (https://www.jspsych.org/) version 6.1.0, and custom JavaScript code (Fig. 1). The JavaScript code for running the task is available in the following GitHub repository: (https://github.com/bwilliams96/SR_Online_task). Testing was hosted on the Gorilla online platform (https://gorilla.sc).

### Careless/insufficient effort responding checks

The reversal learning task included automated careless/insufficient effort (C/IE) responding checks that terminated the task prematurely if met. These conditions were (1) not making a valid choice over five consecutive trials, or (2) not making a valid choice for over 5% of the total number of trials. As part of the study, subjects completed the 12-item version of the Intolerance of Uncertainty Scale (Carleton et al., 2007) (data not reported here). To check for C/IE, we added two instructional attention check questions and one infrequency attention check question (see supplementary materials), following best practice guidelines for online research (Huang et al., 2015; Zorowitz et al., 2023). Subjects were made explicitly aware of the use of attentional checks, and that their responses to the Intolerance of Uncertainty Scale questions would not influence their bonus payment.

#### Behavioural measures

Behavioural measures of task performance were derived from choices made in the reversal learning task. Accuracy is the probability of selecting the “best” (most likely to be rewarding) choice on trial *n*, regardless of whether a reward was obtained or not. Perseveration is a measure of persistence in choosing the previously “best” choice after reversal, and is defined as the probability of selecting the “worst” (least likely to be rewarding) choice on trial *n*, after receiving two losses when making that choice following a reversal. Stay/switching behaviour is determined as the probability of making the same choice as on the previous trial, and is defined as the probability of staying both overall and after either a win or loss on the previous trial. Reaction time is defined as the amount of time the participant took to make a choice on trial *n* and, like staying behaviour, is calculated both overall and whether a win or loss was experienced on the previous trial. We also calculated the difference in reaction time following a win and loss (win RT − loss RT). Behavioural measures were calculated using (1) simple means (referred to as “mean”), estimated from mixed-effects logistic/linear (dependent on whether the variable was binary) regression models that either (2) calculated estimates for each session using separate regression models (referred to as “separate”), or (3) calculated estimates for each session using a single model which explicitly modelled the effect of session (referred to as “joint”).

### Computational modelling

#### Overview

Reinforcement learning models, which are commonly used for modelling reward learning tasks, were fitted to reversal learning behaviour, replicating the methods of Waltmann et al. (2022). We fit two families of models, differentiated by how the expected value determined choice. The first family used an inverse temperature parameter (β) in the softmax choice function to define choice stochasticity by determining the steepness of the softmax function. The second family used a reinforcement sensitivity parameter (ρ) to determine the maximum difference in expected values between choices, placing a lower bound on choice stochasticity. To determine the best-fitting model within each family, we fitted a range of models with combinations of parameters that are commonly used in the reinforcement learning literature. Models had either a single inverse temperature (β)/sensitivity parameter (ρ) or separate inverse temperature (${\beta }_{win/loss}$)/sensitivity parameters (${\rho}_{win/loss}$) for wins and losses.

Expected values for actions were updated using prediction errors $\left({\lambda }_{t}-{V}_{t}^{k}\right)$, the difference in value of the actual (λ) and expected outcome ($V$) of action $k$ on trial $t$ (${V}_{t}^{k}$). The rate of expected value updating was captured by the learning rate, with models having either a single learning rate ($\alpha$) or separate learning rates for wins and losses (${\alpha }^{+/-}$, dual learning rate models). Models either updated the expected value of only the chosen action (single update models) or of both the chosen and unchosen actions (dual update models) using the inverse outcome for updating the unchosen action. The dual update models were fitted with and without a discount weight ($\kappa$) for the unchosen action. An in-depth explanation of model variants can be found in the supplementary materials.

#### Model fitting

Our model fitting replicated the approach taken by Waltmann et al. (2022). Briefly, each model described in the previous section was fitted to the data with one of three estimation methods, maximum likelihood (ML), maximum a posteriori estimation with uninformative priors (MAP0), and maximum a posteriori estimation with empirical priors inferred from the multivariate distribution of parameter estimates across subjects (expectation–maximisation [EM]). The best-fitting model for each family was determined using the integrated Bayesian information criterion (iBIC) from the EM fitting approach, with a lower iBIC indicating a better model fit (Huys et al., 2011, 2012; Waltmann et al., 2022).

#### Synthetic dataset generation and reliability assessment

**Fig. 2:**
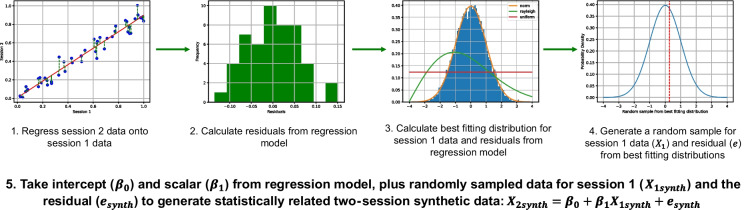
Regression-based method for generating plausible, statistically related two-session synthetic data from our “ground truth” behavioural data

We firstly replicated the ICC, correlational, split-half, and variance decomposition-based reliability assessment of behavioural and computational measures as reported by Waltmann et al. (2022) (for a detailed overview see supplementary materials and the original publication). We assessed the effect of sample size on components of between-subject, within-subject session, and error variance for our behavioural and computational modelling measures of task performance ($X$). To do this, we used a regression-based approach to generate statistically related and plausible synthetic two-session data based on the underlying statistical properties of our collected data (Fig. 2). Firstly, we regressed session 2 measures of task performance (${X}_{2}$) onto session 1 measures (${X}_{1}$), then extracted the residuals ($e$) from this regression for each participant. We used histograms to estimate the probability density function of our task performance measure in session 1 and the residuals from the regression model, using the Freedman–Diaconis estimator (a robust estimator that accounts for data variability and sample size) to determine the optimal number of bins. We used the Fitter Python package (Cokelaer et al., 2024) to identify the best-fitting probability distribution function and calculated its parameters (from the 80 available in SciPy using sum of squares error as the fitting metric) for our task performance measure in session 1 (${X}_{1}$) and the residuals ($e$) from the regression model. We then drew *n* random samples from the probability distribution functions of task performance measure in session 1 (${X}_{1synth}$), and the residuals from the regression model (${e}_{synth}$). Lastly, we took the intercept (${\beta }_{0}$) and scalar (${\beta }_{1}$) coefficients from regressing our task performance measure from session 2 onto session 1, and generated session 2 values using the following equation: ${X}_{2synth}={\beta }_{0}+{\beta }_{1}{X}_{1synth}+{e}_{synth}$. We used this regression-based approach to generate 1000 synthetic datasets with sample sizes of *n* = 300.

For each synthetic dataset we iterated through sample sizes ranging from 10 to 300 and calculated variance components and ICC(A,1) values. We used the following formulae to calculate components of between-subject, within-subject session, and error variance. Between-subject variance was calculated as $Between=\frac{k}{n-1}\sum_{i=1}^{n}{\left({\overline{x} }_{i}-\overline{x }\right)}^{2}$, where $k$ is the number of sessions and $n$ is the number of subjects. Within-subject session variance was calculated as $Within=\frac{n}{k-1}\sum_{j=1}^{k}{({\overline{x} }_{j}-\overline{x })}^{2}$. Error variance was calculated as $Error=\frac{SS-\left(n-1\right)MSr-\left(k-1\right)MSc}{(n-1)(k-1)}$, where $SS$ is the total sum of squares, $MSr$ is the between-subject variance, and $MSc$ is the within-subject session variance. We then corrected within-subject session variance to control for varying sample sizes by multiplying $Within$ by $\frac{k}{n}$ (as is the case when calculating ICC(A,1)), and normalised variance components so they summed to one. Lastly, we calculated ICC(A,1) for each synthetic dataset. This reliability assessment procedure was carried out using our three methods for estimating behavioural measures of task performance and the computational model parameter estimates from the best-fitting model. ICCs were interpreted following guidelines from Cicchetti (1994; but also see Gell et al., 2023, for an overview of how even a small change in reliability can affect accuracy), with poor: ICC < 0.4; fair: 0.4 ≤ ICC < 0.6; good: 0.6 ≤ ICC < 0.75; excellent: 0.75 ≤ ICC.

To test whether the effect of sample size on variance component estimates changed as a function of between-session variance, we also generated synthetic datasets with noise added to the session 2 estimates. To ensure noise was equally scaled across measures, we first generated statistically related two-session data as above (Fig. 2), then *z*-scored the data from the two sessions (mean = 0, *SD* = 1) and added Gaussian noise to values from session 2 sampled from a normal distribution (mean = 0) with varying levels of noise (*SD* = [0.25, 0.5, 1, 1.5, 2]). Finally, values for both sessions were reverse *z*-scored. Using this procedure we generated 1000 independent synthetic datasets with sample sizes of *n* = 300 for each behavioural and computational modelling variable, for each noise level (*SD* = [0.25, 0.5, 1, 1.5, 2]).

**Fig. 3:**
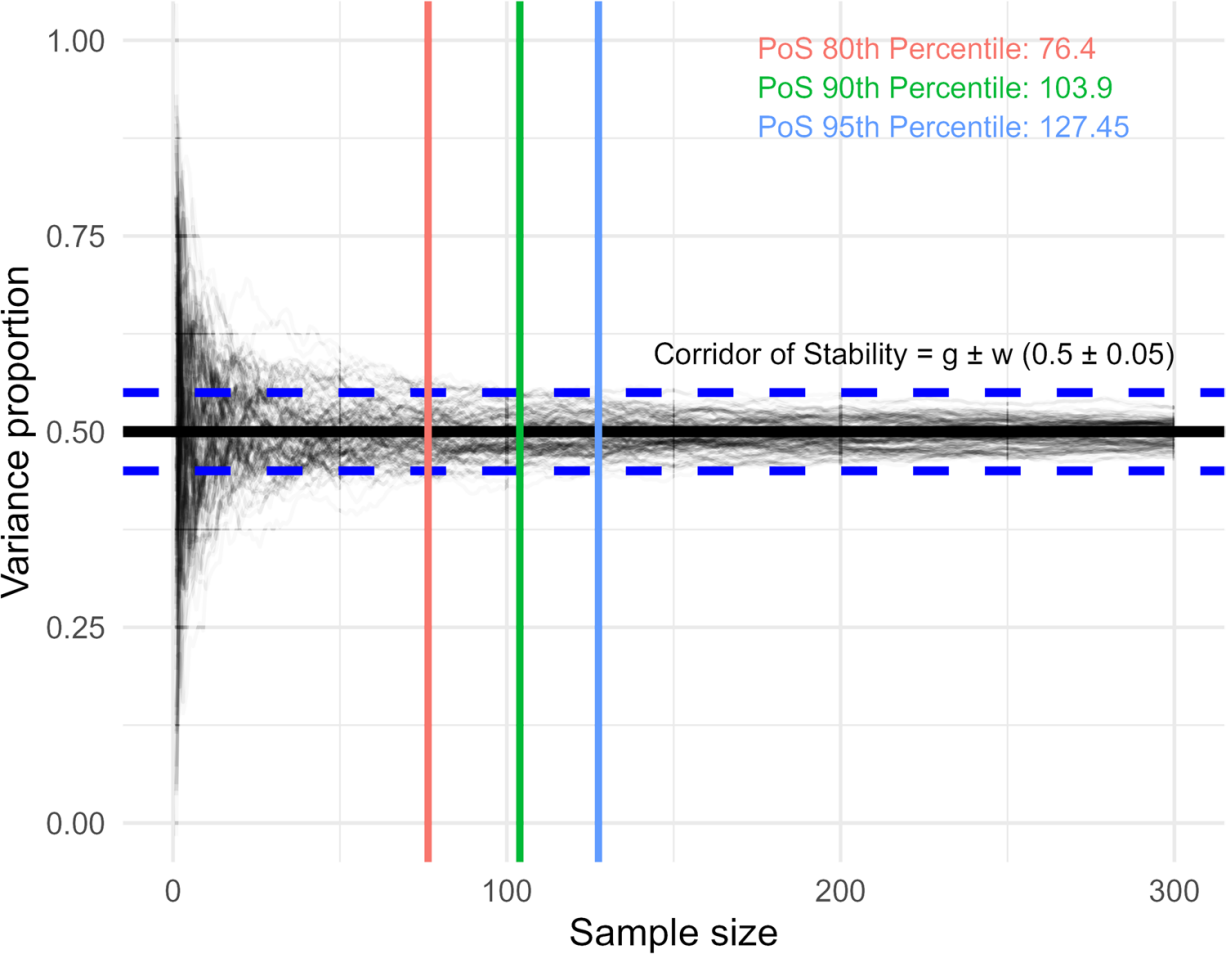
Actual (thick black line) and simulated (transparent grey lines, each line represents one iteration of the simulation) variance proportions for a synthetic dataset, where the true variance proportion (*g*) is equal to 0.5. For a given width (0.05), the *n*th percentile of the *point of stability* (PoS) can be calculated within a *corridor of stability* (CoS range = g ± w), with the *corridor of stability* half-width in this example equal to 0.05 (CoS range = 0.45–0.55). More explicitly, this is the sample size at which the *n*th percentile of simulations had variance proportions that no longer exceeded the range of the *corridor of stability*

**Fig. 4:**
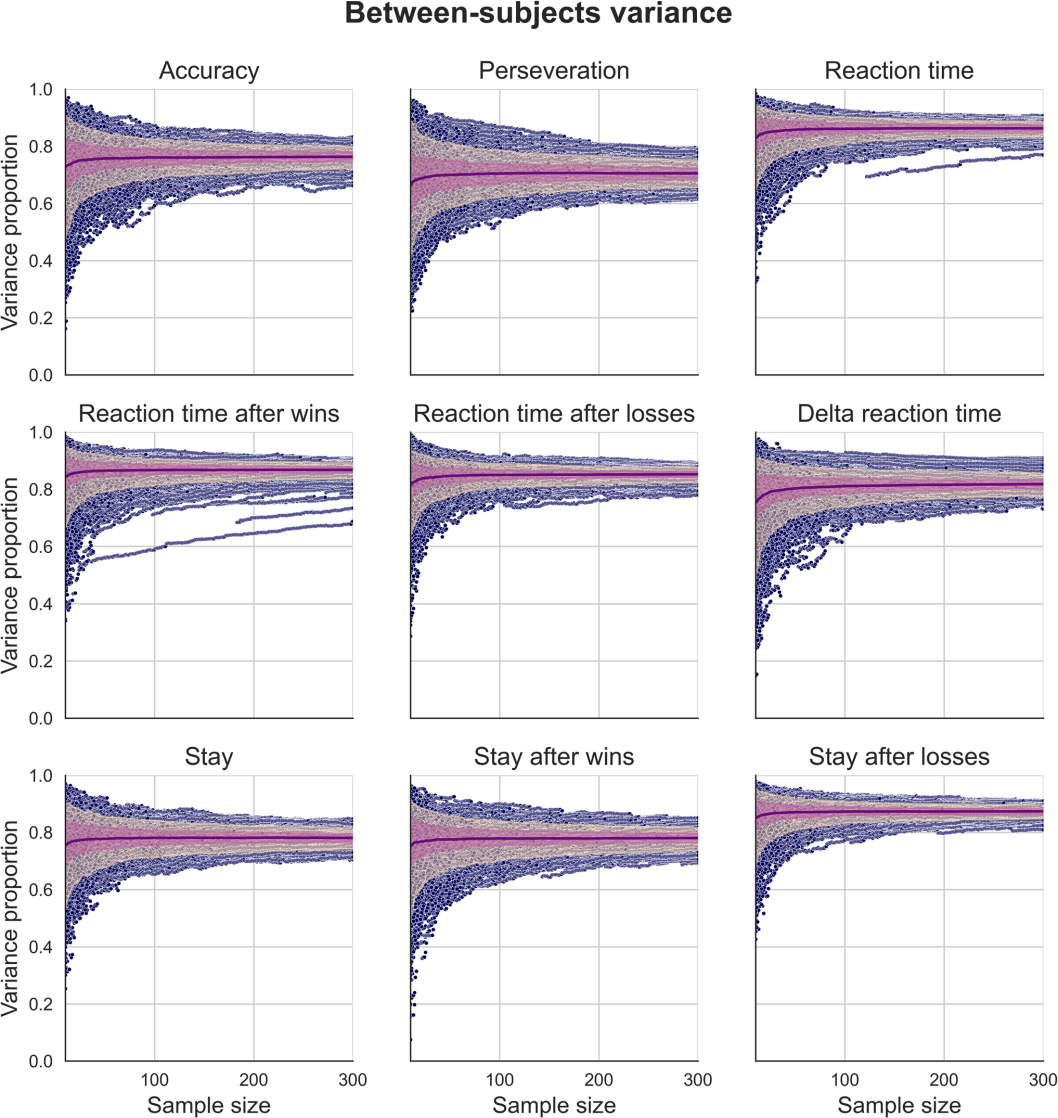
Distributions of between-subject variance proportions for simulated measures of behavioural performance generated using our regression-based approach. These data were generated using behavioural measures estimated using the “joint” regression model, which explicitly modelled the effect of session. The mean proportion of variance for each sample size (purple), 90th inter-percentile range (dark pink), and interquartile range (light pink) are overlaid on individual data points (blue). Distributions of simulated data generated using behavioural measures from the “separate” regression model and using simple means can be found in the supplementary figures

**Fig. 5:**
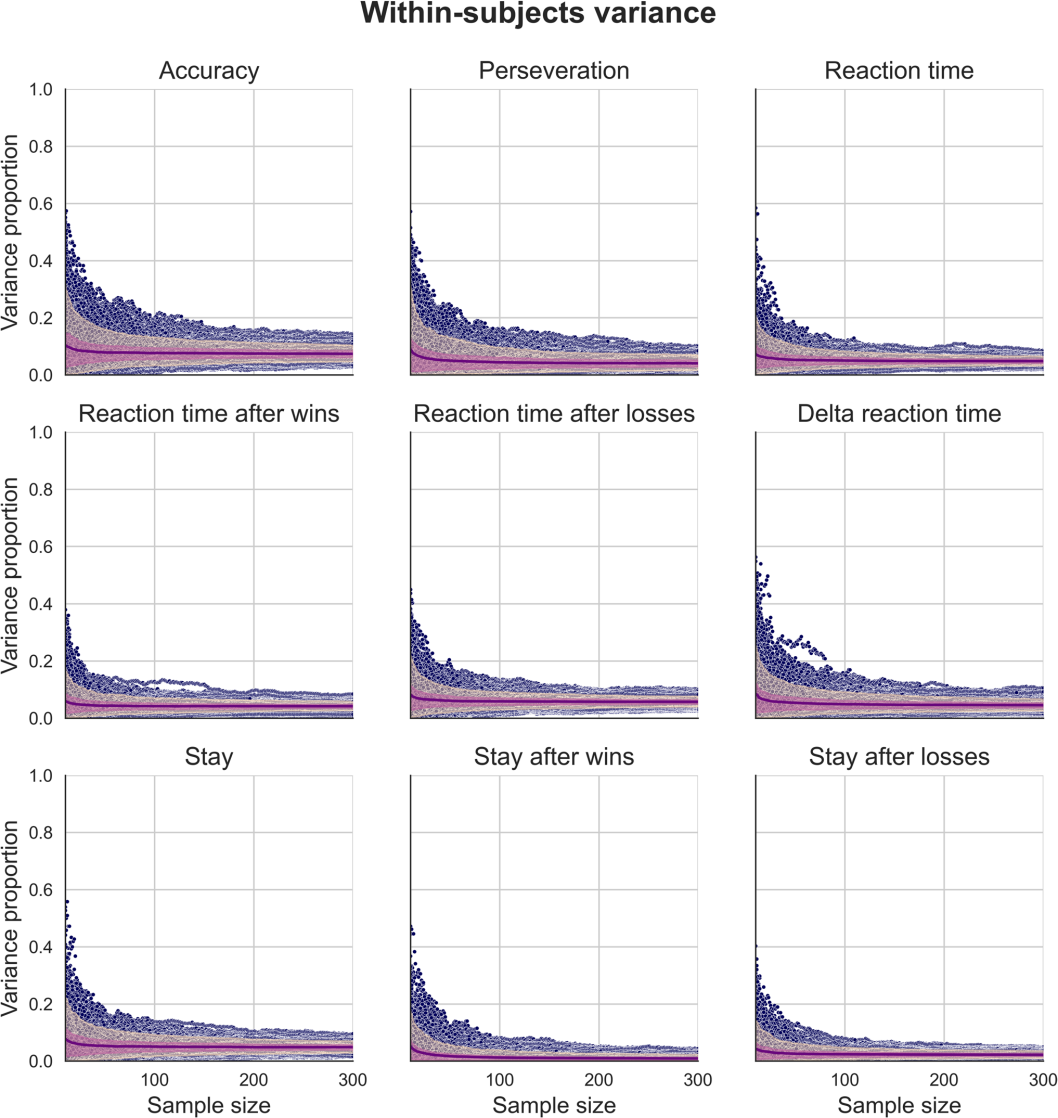
Distributions of within-subject variance proportions for simulated measures of behavioural performance generated using our regression-based approach. These data were generated using behavioural measures estimated using the “joint” regression model, which explicitly modelled the effect of session. The mean proportion of variance for each sample size (purple), 90th inter-percentile range (dark pink), and interquartile range (light pink) are overlaid on individual data points (blue). Distributions of simulated data generated using behavioural measures from the “separate” regression model and using simple means can be found in the supplementary figures

**Fig. 6:**
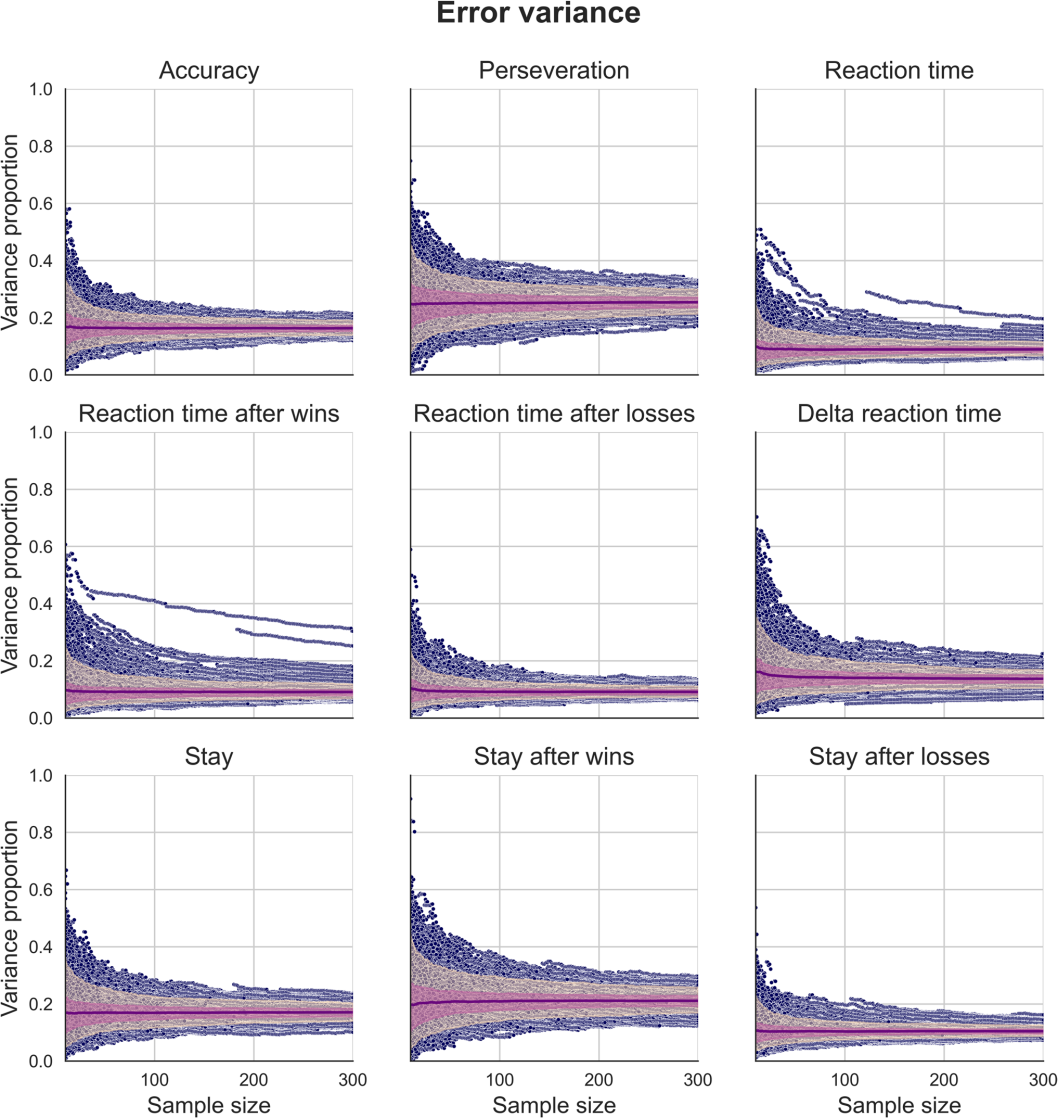
Distributions of error variance proportions for simulated measures of behavioural performance generated using our regression-based approach. These data were generated using behavioural measures estimated using the “joint” regression model, which explicitly modelled the effect of session. The mean proportion of variance for each sample size (purple), 90th inter-percentile range (dark pink), and interquartile range (light pink) are overlaid on individual data points (blue). Distributions of simulated data generated using behavioural measures from the “separate” regression model and using simple means can be found in the supplementary figures

To determine the critical sample size at which variance component estimates stabilised, we determined the *point of stability* using the method described by Schönbrodt and Perugini (2013). The *point of stability* was determined based on two values. These were the parameters *g*, which was the ground truth of a given measure, and *w,* which was the half-width of a *corridor of stability* around *g*, (CoS = *g* ± *w*). Here, g was defined using variance component proportions calculated from our observed data collected from participants, and we had corridors of stability with half-widths *w* = [0.025, 0.05, 0.75, 0.1, 0.15, 0.2]. The *point of stability* was defined as the smallest sample size in a vector of values (in our case these were variance component estimates for sample sizes ranging from 10 to 300 for one iteration of our simulation) from which all subsequent sample sizes had values that fell within the defined *corridor of stability*. This procedure was performed for each synthetic dataset for each variance component, meaning a distribution of *points of stability* could be generated. Critical sample sizes were then calculated for a given percentile (here, we use 80th, 90th and 95th percentiles) of the distribution of *points of stability*(Fig. 3). If a vector of variance components never stabilised within the *corridor of stability* then the *point of stability* was defined as the maximum sample size (300), while a vector of variance components that never deviated outside the *corridor of stability* had the *point of stability* defined as the minimum sample size (10), as in Schönbrodt and Perugini (2013). We determined critical sample sizes for our synthetic datasets that were generated both with and without additional Gaussian noise (Figs. 4, 5 and 6).

## Results

### Reversal learning performance reliability

First, we assessed reversal learning task performance using data collected from our participants. Behavioural performance in the reversal learning task was measured using accuracy, perseveration, staying behaviour (making the same choice on trial *t* as *t* − 1), and reaction time. Simple means and regression models were used to estimate behavioural measures from trial-wise measures; both a separate regression model for each session (separate) and a single model which explicitly estimated the effect of session (joint) were used. Accuracy, perseveration, and stay behaviour were calculated as proportions, while reaction time measures were calculated in milliseconds. Summary statistics for mean, standard deviation, and range are presented in Table 2 for all behavioural estimates. Overall, behavioural estimates of task performance show small but statistically significant increases in performance between sessions 1 and 2 (e.g., increased accuracy, reduced perseveration, increased staying after wins; paired-samples *t*-tests, all *p* values < [0.05/27]), although staying after losses also significantly increased between sessions 1 and 2. A mixed-effects logistic regression revealed a main effect of previous feedback on staying behaviour, with subjects switching more after losses than wins ($\beta =3.665, z=31.756, p<0.001$), while a mixed-effects linear regression revealed a main effect of previous feedback on reaction time, with subjects responding more quickly after wins than losses ($\beta =-23.748, t\left(149.4\right)=-6.84, p<0.001$).

**Table 2:** Summary statistics for behavioural measures of reversal learning task performance in sessions 1 and 2

Behavioural measure	Estimation method	Session	Mean	*SD*	Min	Max
Accuracy	Mean	1	0.69	0.07	0.57	0.85
		2	0.73	0.06	0.6	0.87
	Joint	1	0.7	0.05	0.61	0.81
		2	0.72	0.05	0.62	0.82
	Separate	1	0.7	0.05	0.61	0.8
		2	0.73	0.04	0.64	0.82
Perseveration	Mean	1	0.13	0.06	0.01	0.28
		2	0.11	0.06	0.01	0.27
	Joint	1	0.13	0.05	0.03	0.26
		2	0.11	0.05	0.03	0.25
	Separate	1	0.13	0.05	0.04	0.26
		2	0.1	0.05	0.03	0.25
Reaction time	Mean	1	546.2	111.4	319.9	917.1
		2	509.6	99.89	349.5	915.1
	Joint	1	540.5	108.8	316.6	894.8
		2	506.7	98.48	349	908.5
	Separate	1	544.8	109.8	317.9	911.7
		2	507.1	98.56	351.3	906.4
Reaction time wins	Mean	1	528.8	109.3	312.2	891
		2	499.4	99.75	334.8	933.4
	Joint	1	528.7	105.7	316.2	875
		2	499.4	97.19	338.4	910.2
	Separate	1	528.8	105.8	315.1	876.4
		2	499.4	96.86	340.5	913
Reaction time loss	Mean	1	560.7	120.9	313.7	1028
		2	514.6	106.3	354.4	892.8
	Joint	1	558	117.1	317.1	986.9
		2	517.6	103.4	359.4	905.9
	Separate	1	560.8	116.6	320.8	994.8
		2	514.7	102.5	360.2	899.8
Reaction time win − loss	Mean	1	− 31.85	54.39	− 230.9	86.51
		2	− 15.22	46.63	− 181	123.3
	Joint	1	− 29.3	37.58	− 180.6	64.52
		2	− 18.2	31.94	− 148.1	69.05
	Separate	1	− 32.03	36.53	− 171.4	44.83
		2	− 15.38	30.51	− 124.6	71.86
Stay	Mean	1	0.73	0.12	0.45	0.93
		2	0.77	0.11	0.51	0.93
	Joint	1	0.83	0.11	0.46	0.97
		2	0.86	0.09	0.56	0.98
	Separate	1	0.77	0.12	0.42	0.95
		2	0.82	0.12	0.48	0.96
Stay wins	Mean	1	0.93	0.07	0.66	1
		2	0.96	0.05	0.68	1
	Joint	1	0.94	0.06	0.69	0.99
		2	0.96	0.05	0.69	1
	Separate	1	0.93	0.06	0.68	0.99
		2	0.96	0.05	0.7	0.99
Stay loss	Mean	1	0.44	0.23	0.03	0.83
		2	0.49	0.23	0.05	0.85
	Joint	1	0.44	0.21	0.07	0.82
		2	0.49	0.22	0.07	0.83
	Separate	1	0.44	0.21	0.07	0.81
		2	0.49	0.21	0.08	0.83

Measures were estimated either using simple means (mean), or from mixed effects regression models calculated using separate regression models (separate) or a single model which explicitly modelled the effect of session (joint). Accuracy, perseveration, and stay behaviour are presented as proportions, and reaction times are presented in milliseconds.

Test–retest reliability of task performance was assessed by replicating the statistical methods of Waltmann et al. (2022). These results are briefly summarised here, but see supplementary materials for a detailed overview. Accuracy reliability estimates ranged from good when jointly estimating the effect of session to poor when using mean values. Staying behaviour showed good reliability across all estimation methods, and showed good to excellent reliability for staying after losses, but only fair (mean and separate estimation) to good (joint estimation) reliability for staying after wins. Perseveration reliability was poor when using mean and separate estimation methods, and fair when using joint estimation. Reaction time showed good reliability when estimated using mean and separate estimation methods, and excellent reliability when using joint estimation, and the same pattern was observed for reaction time after a win. Reaction time after a loss had good reliability across all estimation methods; poor reliability was found for the difference in reaction between wins and losses when estimated using separate and separate estimation measures, but was good when joint estimation was used.

Computational measures of task performance were derived by fitting reinforcement learning models to choice behaviour during the task. We fit two families of models that either altered the softmax choice function to define choice stochasticity (by varying the inverse temperature parameter β), or used a reinforcement sensitivity parameter (ρ) to determine the maximum difference in expected values between choices, placing a lower bound on choice stochasticity.

Comparisons of model fit were performed using each model’s integrated Bayesian information criterion (iBIC) from the EM fitting approach (supplementary Fig. 3), which provided the best model fit scores and reliability of parameter estimates. The best-fitting model for the softmax family was the dual update model with a discount weight for the unchosen option with separate learning rates plus softmax temperatures for wins and losses (DU-2β2ακ). The best-fitting model for the reinforcement sensitivity family was the dual update model with separate reinforcement sensitivities for wins and losses plus a single learning rate parameter (DU-2ρα), which was also the best-fitting model overall (Table 3). This model had good to excellent estimates of reliability for all parameters (learning rate, reinforcement sensitivity for win, reinforcement sensitivity for loss) when model parameters were fitted using the EM approach. The parameters from the best-fitting model from the softmax family had reliability that ranged from poor to good. Model parameters had poor reliability when estimated using maximum likelihood methods (Supplementary Figs. 4 and 5).

**Table 3:** Summary statistics for parameter estimates from the best-fitting computational model in sessions 1 and 2

	Mean	*SD*	Min	Max
Reinforcement sensitivity win (session 1)	2.05	0.92	0.36	4.43
Reinforcement sensitivity win (session 2)	2.51	1.14	0.42	5.5
Reinforcement sensitivity loss (session 1)	− 0.7	0.43	− 1.56	0.56
Reinforcement sensitivity loss (session 2)	− 0.71	0.41	− 1.62	0.58
Learning rate (session 1)	0.71	0.18	0.35	0.96
Learning rate (session 2)	0.71	0.15	0.34	0.95

Parameters were estimated from a dual update model from the reinforcement sensitivity family, with separate reinforcement sensitivities for wins and losses, and a single learning rate. Parameters were fitted using expectation–maximisation.

Overall, the reliability of both our behavioural and computational modelling measures of reversal learning performance were in line with those presented by Waltmann et al. (2022). Moreover, we found that the best-fitting models from both the softmax and reinforcement sensitivity families were the same reported by Waltmann et al. (2022). Next, we tested how robust these reliability effects were across varying sample sizes by investigating the effect of sample size on the individual components of variance used when calculating ICCs.

### Effects of sample size on behavioural measures of task performance

We generated synthetic two-session data using our regression-based approach to investigate the effect of sample size on estimates of variance components for behavioural performance measures. These behavioural performance measures were derived from our three estimation methods. Figure 4 show the variance component estimates for data simulated using the distributions of behavioural measures, themselves estimated using a regression model that explicitly modelled the effect of session on behavioural performance (for figures using simple mean estimates and estimates using separate regression models for each session, see supplementary Figs. 8–13). Each point represents a given sample size between 10 and 300 for each iteration from the simulation, and overlaid are mean estimates for each sample size (purple), interquartile range (light pink), and the 90th inter-percentile range (dark pink).

**Fig. 7:**
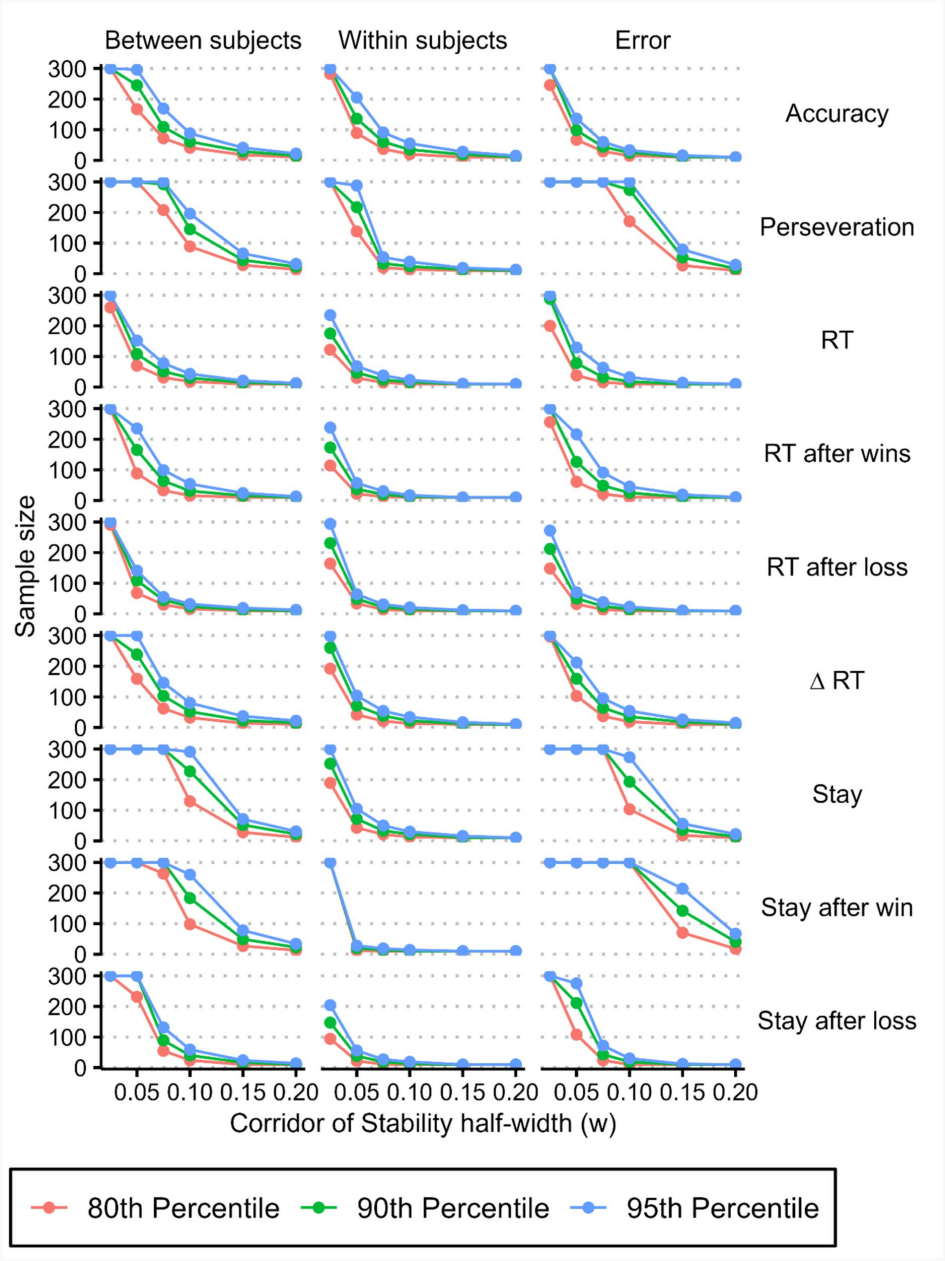
Critical *point of stability* of variance component estimates for synthetic behavioural measures across a range of *corridor of stability* half-width values, calculated based on the distribution of data from the “joint” regression model. Critical *point of stability* calculations using behavioural measures from the “separate” regression model and using simple means can be found in the supplementary figures

We then calculated at what sample size variance component estimates stabilised. Using variance component proportions from our collected data as the ground truth, we determined the *point of stability* for variance component estimates from each iteration of our simulation, using a range of half-widths (0.025, 0.05, 0.75, 0.1, 0.15, 0.2) for the *corridor of stability* (Fig. 3). For each half-width we determined the critical *point of stability* by calculating the 80th, 90th, and 95th percentiles of the calculated *points of stability* (Fig. 7, “joint” estimation method; see supplementary Figs. 14 and 15 for *points of stability* using data from the “separate” and “mean” estimation methods). As the half-width of the *corridor of stability* increased, the sample size required for variance component estimates decreased (Table 4). At narrower *corridor of stability* half-widths (i.e., at more precise estimates of variance component proportions), the sample size where variance component estimates stabilised were, on average, between sample sizes of 126.84 and 193.99 for half-widths of 0.05 (ground truth variance proportion ± 0.05) and were between 250.15 and 290.41 for half-widths of 0.025 (ground truth variance proportion ± 0.025) (4).

**Table 4:** Summary statistics for critical *points of stability* for between-subject, within-subject, and error variance components for synthetic behavioural measures across a range of *corridor of stability* half-width values and *point of stability* percentiles

Measure	Variance component	80th Percentile						90th Percentile						95th Percentile					
		w = 0.025	w = 0.05	w = 0.075	w = 0.1	w = 0.15	w = 0.2	w = 0.025	w = 0.05	w = 0.075	w = 0.1	w = 0.15	w = 0.2	w = 0.025	w = 0.05	w = 0.075	w = 0.1	w = 0.15	w = 0.2
Median	Between subjects	300	167	62	32.2	14	10	300	245	103.1	52	23	15	300	300	146.1	80	37	22
	Within subjects	189.2	34	15	10	10	10	252.3	49	24	16	10	10	298	68.1	38	23	12	10
	Error	296.2	103	29.2	15	10	10	300	159.2	48.3	25	11	10	300	216.05	91.05	45	19	11
Mean	Between subjects	294.44	187.04	117.18	51.51	17.22	11	300	229.47	150.37	88.19	28.7	15.34	300	258.26	175.36	122.58	42.35	21.58
	Within subjects	195.33	48.02	17.89	12.22	10.02	10	237.62	76.38	29.24	18.67	11.78	10.11	274.36	108.36	43.79	28.01	14.78	11.01
	Error	260.67	145.44	115.69	72.13	19.47	10.89	288.91	180.27	128.51	100.42	33.44	14.67	296.89	215.37	146.58	121.12	49.68	20.46
*SD*	Between subjects	12.49	93.72	102.24	40.12	7.8	1.49	0	77.18	105.94	72.06	14.78	5.54	0	62.98	93.84	93.64	22	8.37
	Within subjects	76.49	38.01	7.87	3.15	0.06	0	56.47	58.89	13.17	7.47	2.86	0.31	35.56	79.64	20.44	12.3	5.67	1.7
	Error	51.67	111.84	130.48	96.65	18.77	2.51	27.41	95.17	121.68	112.93	40.92	9.6	8.8	81.61	109.63	120.65	62.15	17.65

### Effects of sample size on computational modelling parameter measures of task performance

**Fig. 8:**
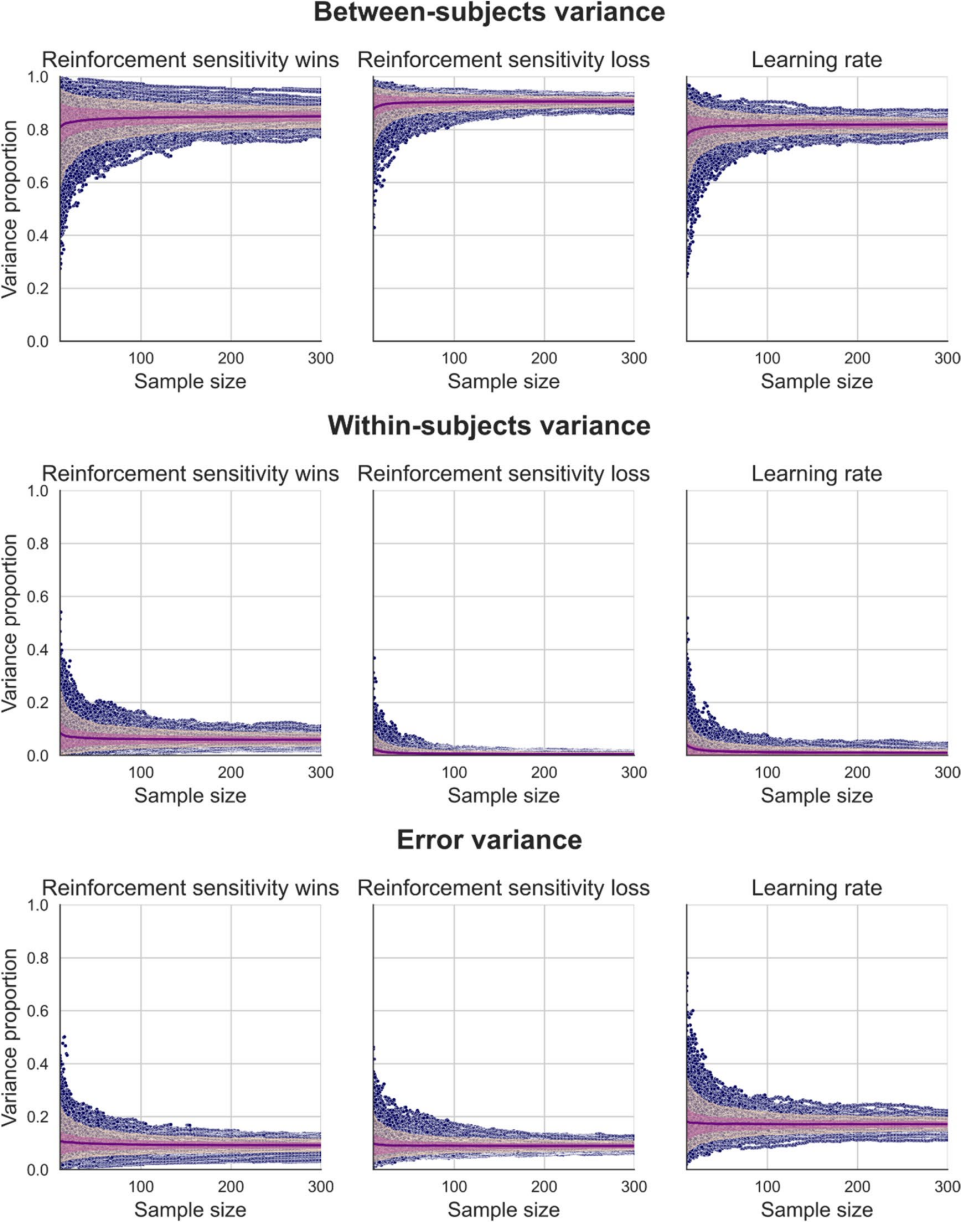
Distributions of within-subject, between-subject, and error variance proportions for parameter estimates from our best-fitting computational model estimated for different sample sizes generated using our regression-based approach. The mean proportion of variance for each sample size (purple), 90th inter-percentile range (dark pink), and interquartile range (light pink) are overlaid on individual data points (blue)

**Fig. 9:**
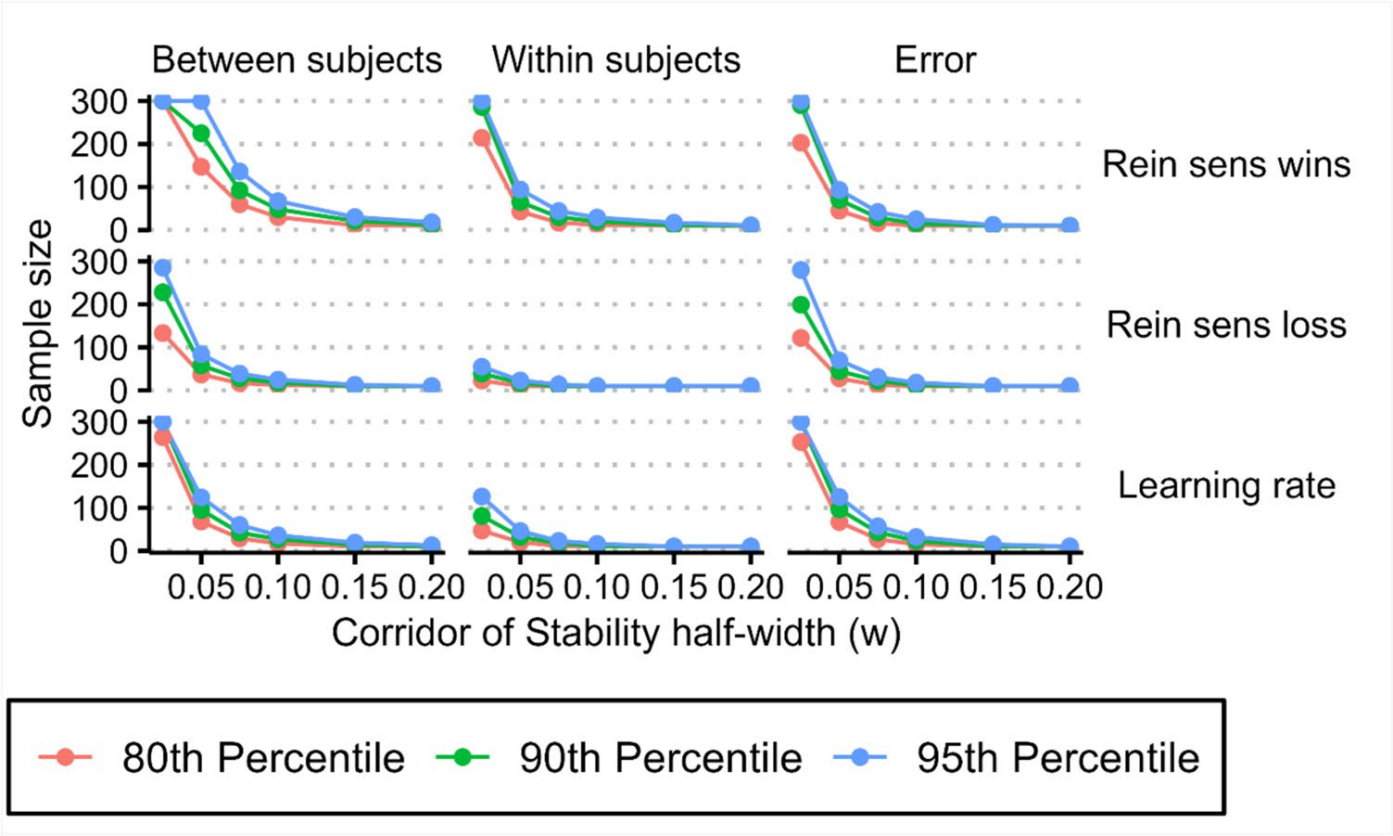
Critical *point of stability* of variance component estimates for synthetic computational modelling parameters across a range of *corridor of stability* half-width values

To test for the effects sample size on estimates of variance components for computational modelling parameter estimates (for our best-fitting model), we generated synthetic two-session data using our regression-based approach (as above for our behavioural data, Fig. 8). We then calculated the critical *point of stability* for variance components of our simulated computational modelling parameters in the same way as we did for our behavioural measures (Fig. 9). As observed for our behavioural data, as the half-width of the *corridor of stability* increased, the sample size required for variance component estimates decreased. At narrower *corridor of stability* half-widths (i.e., at more precise estimates of variance component proportions), the sample size where variance component estimates stabilised were, on average, between sample sizes of 51.67 and 106.59 for half-widths of 0.05 (ground truth variance proportion ± 0.05), and were between 173.31 and 249.58 for half-widths of 0.025 (ground truth variance proportion ± 0.025) (Table 5).

**Table 5:** Mean critical *points of stability* for between-subject, within-subject, and error variance components for synthetic computational modelling parameters across a range of *corridor of stability* half-width values and *point of stability* percentiles

Measure	Variance component	80th Percentile						90th Percentile						95th Percentile					
		w = 0.025	w = 0.05	w = 0.075	w = 0.1	w = 0.15	w = 0.2	w = 0.025	w = 0.05	w = 0.075	w = 0.1	w = 0.15	w = 0.2	w = 0.025	w = 0.05	w = 0.075	w = 0.1	w = 0.15	w = 0.2
Median	Between subjects	264.2	68	29	17	10	10	300	95	42.1	27	14	10	300	124.05	60.05	36	19	13
	Within subjects	47	20	11	10	10	10	81.1	32.1	18	11.1	10	10	126.05	46	23	16	10	10
	Error	203	45	16	10	10	10	290	70	29	15	10	10	300	93	42	25	12	10
Mean	Between subjects	232.53	84	35.33	19.67	10.33	10	276	126	54.03	31.03	15	10.67	295.05	169.38	78.37	42.7	20.7	13.68
	Within subjects	94.73	24.33	12.67	10.67	10	10	135.37	38.07	19.33	13.7	10.33	10	160.35	54.38	27.02	18.33	12.33	10.33
	Error	192.67	46.67	18.33	11.67	10	10	263.1	70.37	31.33	16.67	10	10	293.33	96.02	43.33	25	12.33	10
*SD*	Between subjects	71.61	46.31	18.12	7.59	0.47	0	33.94	71.61	27.46	12.61	4.55	0.94	7	93.79	41.68	17.83	7.04	3.32
	Within subjects	85.04	13.82	3.09	0.94	0	0	107.89	20.08	8.22	4.48	0.47	0	102.92	29.61	12.59	7.93	3.3	0.47
	Error	53.98	15.97	6.34	2.36	0	0	45.3	20.86	8.73	4.64	0	0	9.43	22.58	10.66	5.72	2.05	0

### Effects of sample size on ICC(A,1) and variance component associations

**Fig. 10:**
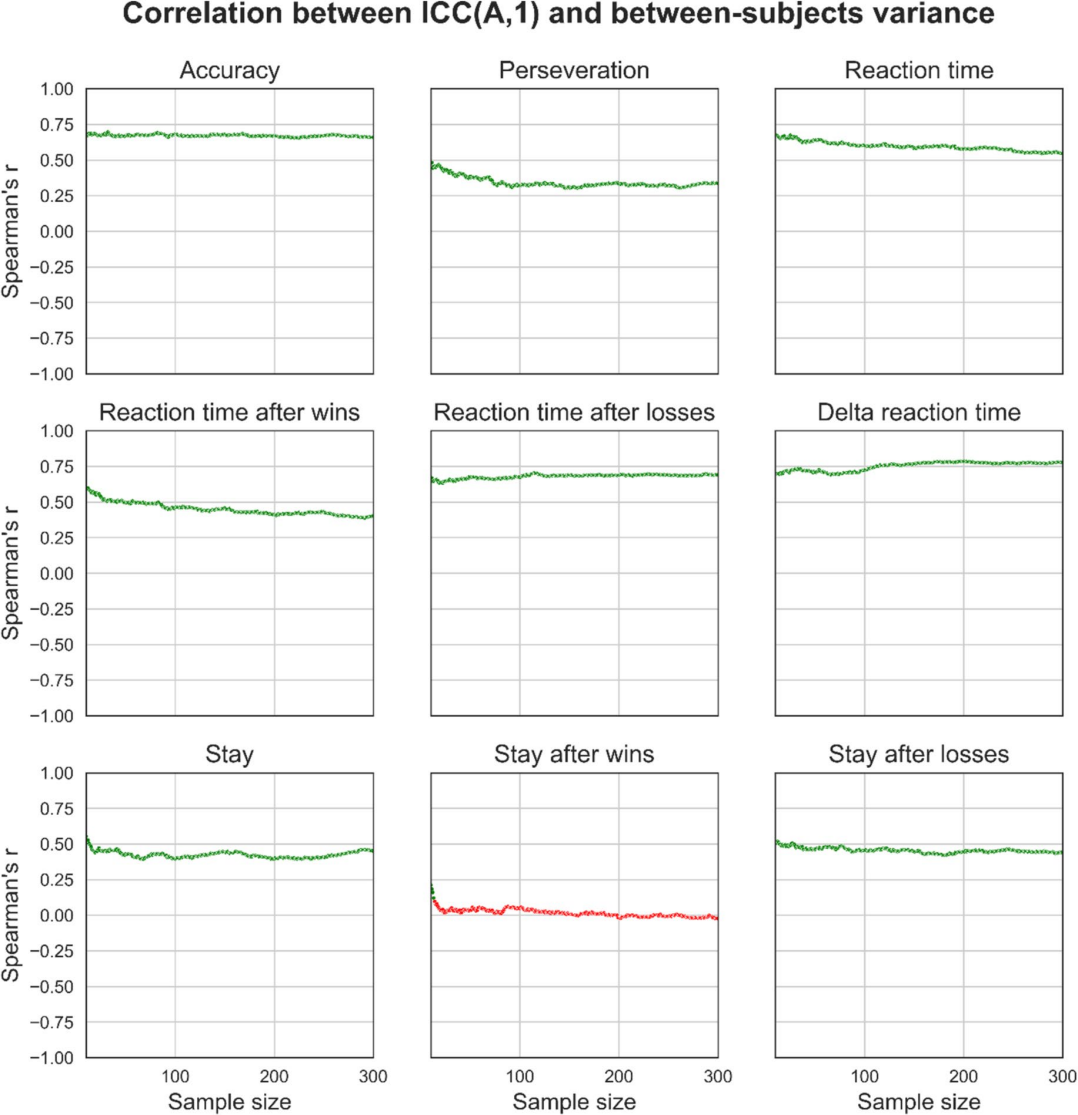
Effects of sample size on the association between ICC coefficients and variance component estimates for behavioural measures. For each behavioural measure and at each sample size, we took the set of 1000 simulated datasets and calculated correlation coefficients to measure the strength of the association between each dataset’s respective ICC(A,1) and variance component estimates. The point estimate for the correlation coefficient and its statistical significance (coloured green for significant, red for non-significant; Bonferroni-corrected) were then plotted. Overall, between-subject variance was strongly positively correlated with ICC(A,1). These plots were generated from synthetic data generated using the distribution of behavioural measures from the “joint” regression approach, which explicitly modelled the effect of session. See supplementary figures for plots generated using data from the “separate” regression approach and using simple means

**Fig. 11:**
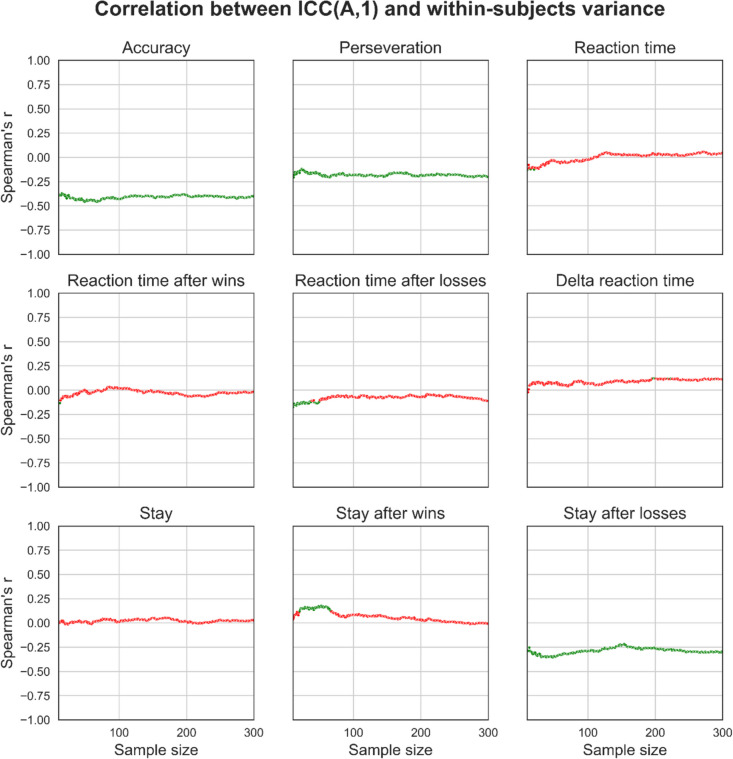
Effects of sample size on the association between ICC coefficients and variance component estimates for behavioural measures. For each behavioural measure and at each sample size, we took the set of 1000 simulated datasets and calculated correlation coefficients to measure the strength of the association between each dataset’s respective ICC(A,1) and variance component estimates. The point estimate for the correlation coefficient and its statistical significance (coloured green for significant, red for non-significant; Bonferroni-corrected) were then plotted. Overall, within-subject variance was weakly or not correlated with ICC(A,1). These plots were generated from synthetic data generated using the distribution of behavioural measures from the “joint” regression approach, which explicitly modelled the effect of session. See supplementary figures for plots generated using data from the “separate” regression approach and using simple means

**Fig. 12:**
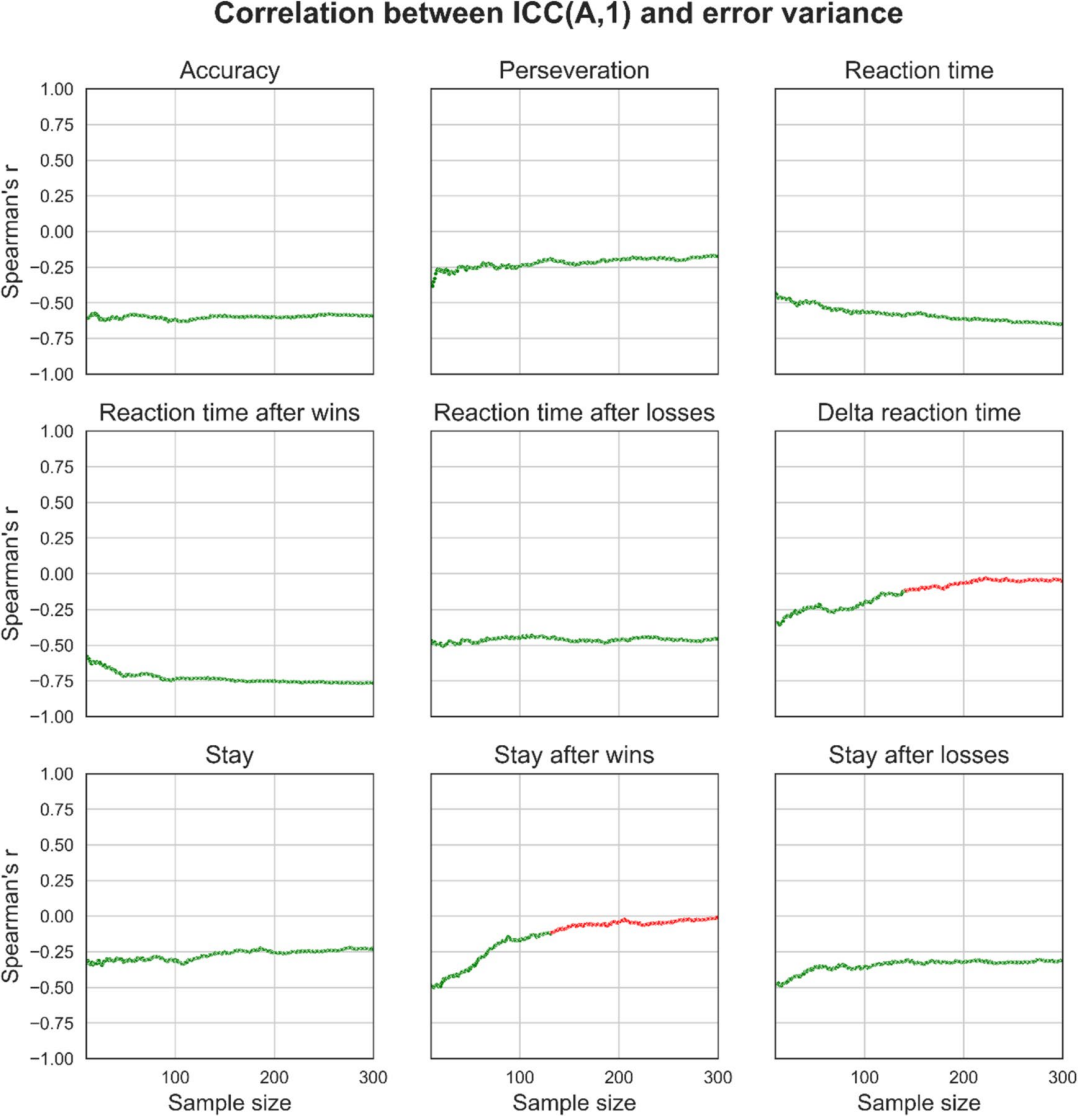
Effects of sample size on the association between ICC coefficients and variance component estimates for behavioural measures. For each behavioural measure and at each sample size, we took the set of 1000 simulated datasets and calculated correlation coefficients to measure the strength of the association between each dataset’s respective ICC(A,1) and variance component estimates. The point estimate for the correlation coefficient and its statistical significance (coloured green for significant, red for non-significant; Bonferroni-corrected) were then plotted. Overall, error variance was strongly negatively correlated with ICC(A,1). These plots were generated from synthetic data generated using the distribution of behavioural measures from the “joint” regression approach, which explicitly modelled the effect of session. See supplementary figures for plots generated using data from the “separate” regression approach and using simple means

**Fig. 13:**
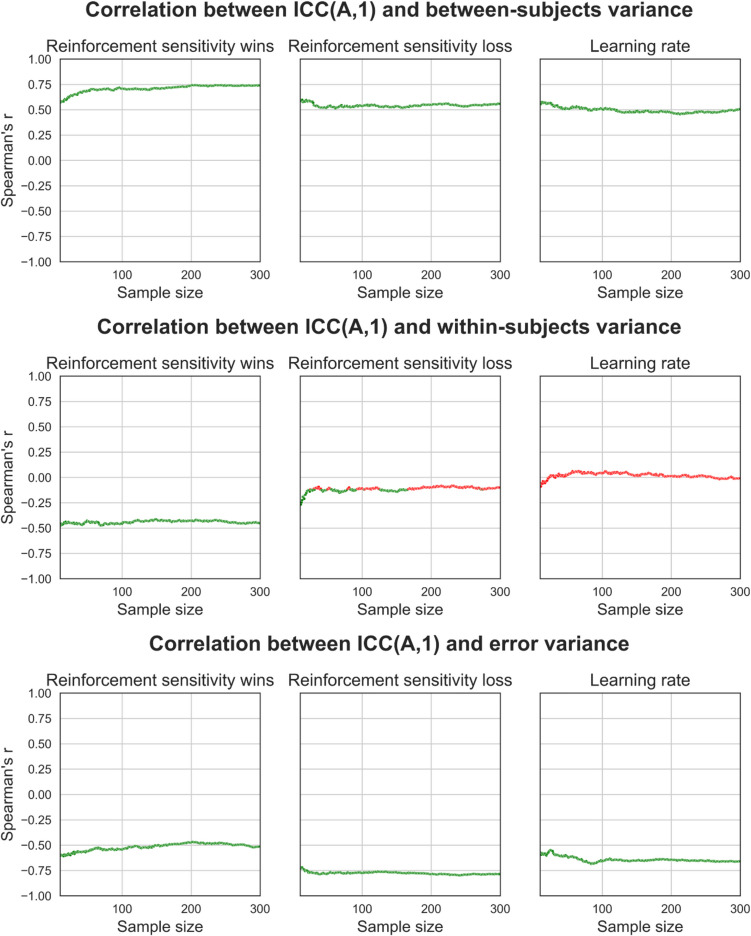
Effects of sample size on the association between ICC coefficients and variance component estimates for computational modelling parameters. For each parameter and at each sample size, we took the set of 1000 simulated datasets and calculated correlation coefficients to measure the strength of the association between each dataset’s respective ICC(A,1) and variance component estimates. The point estimate for the correlation coefficient, and its statistical significance (coloured green for significant, red for non-significant; Bonferroni-corrected) were then plotted. Overall, between-subjects variance was strongly positively correlated with ICC(A,1), error variance was strongly negatively correlated with ICC(A,1), and within-subjects variance was weakly or not correlated with ICC(A,1)

We tested for associations between individual variance components and ICC(A,1) measures of reliability. To do this, we calculated Spearman’s correlation coefficient and *p* values (Bonferroni-corrected) between each variance component measure and ICC(A,1) across the 1000 synthetic datasets generated at each sample size *n*, for each behavioural (Fig. 10, Fig. 11, Fig. 12) and computational modelling parameter (Fig. 13) measure of task performance. Overall, we observe that between-subjects variance had a strong positive correlation with ICC(A,1), error variance had a strong negative correlation with ICC(A,1), and within-subject variance had a weak/no correlation with ICC(A,1). Secondly, these associations are true for most behavioural and computational measures of task performance. Yet, where there are inconsistencies (e.g., staying after wins for between-subject variance and accuracy for within-subject variance), we find that the strength of these associations is consistent even when the behavioural measures were estimated using different methods (see supplementary Figs. 16–21). We also find that the strength of these associations appears relatively stable across different sample sizes, as indexed by the consistency of Spearman correlation coefficients.

### Effects of noise on variance estimates

**Fig. 14:**
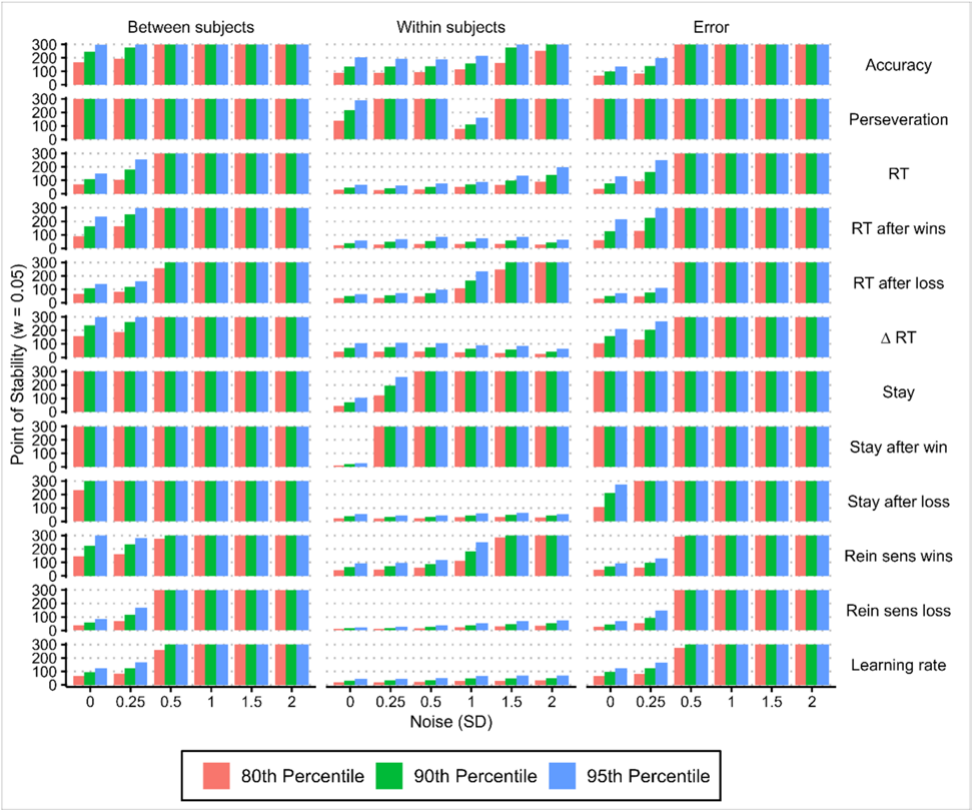
Effects of noise on *point of stability* calculations for synthetically generated data. As the amount of noise added to synthesised behavioural and computational measures of task performance is varied, a monotonic change in the *point of stability* for variance components is observed. For the majority of variance components, increasing amounts of simulated noise cause variance components to fail to reach a *point of stability* before the largest sample size is reached, meaning that variance component estimates remain unstable. These plots were generated from synthetic data generated using the distribution of behavioural measures from the “joint” regression approach, which explicitly modelled the effect of session

Lastly, we tested how adding noise to our simulated data influenced the calculation of *points of stability*. We generated synthetic two-session data using our regression-based synthesis approach as described above, with an additional step of adding Gaussian noise to the calculated value for session 2. We generated multiple datasets with varying levels of noise; by sampling noise from normal distributions with mean = 0 and *SD* = [0.25, 0.5, 1, 1.5, 2], we were able to investigate the effects of increasing levels of noise on *point of stability* calculations, in comparison to the originally simulated data (*SD* = 0). Adding noise to simulated data caused a monotonic change in the *point of stability* of all variance components with increasing levels of noise (Fig. 14). Across a range of percentile values (80th, 90th, 95th) the *point of stability* is equal to the largest sample size (300), indicating that a *point of stability* was not reached for a majority of variance component estimates, meaning that variance component estimates did not stabilise as simulated noise increased. These results were consistent across *point of stability* percentiles, *corridor of stability* half-widths (w), and behavioural estimation approaches (“joint”, “separate”, and “mean” modelling approaches; see supplementary Figs. 22 and 23; included code can be used to recreate figures with different corridor widths).

## Discussion

In this paper we investigated the test–retest reliability of behavioural and computational measures of performance on a reversal learning task, and the effects of sample size on such estimates of reliability. We calculated the reliability of these measures using several approaches, including ICCs and variance decomposition, replicating a previous study on this topic (Waltman et al., 2022). The retest–reliability of ICCs for our behavioural measures were good to excellent for staying and reaction time behaviour, while accuracy and perseveration were less reliable between sessions. ICCs for parameter estimates from our best-fitting computational model (single learning rate and separate reinforcement sensitivity parameters for wins and losses) showed good reliability when estimated using an expectation–maximisation (EM) model fitting approach. These results were broadly in line with the previous findings of Waltmann et al. (2022). Using our behavioural and computational measures of task performance, we then investigated the effects of sample size on individual components of variance. We used a regression-based approach to generate statistically related and plausible synthetic two-session datasets based on the underlying statistical properties of our collected data. Sample size influenced estimates of within-subject, between-subject, and error variance for all behavioural measures of task performance and all computational modelling parameter estimates. Importantly, we demonstrate that variance component estimates do not stabilise until sample sizes much greater than those often used in test–retest research are achieved.

We also tested the effects of sample size on the association between measures of reliability, as assessed using ICC(A,1) and individual variance components. This is important to understand because individual variance component calculation decomposes an ICC coefficient into its constituents and enables the investigation of how sources of variance contribute to the summary reliability statistic represented by ICCs (the ratio of between-subject variance over the total amount of variance). ICCs had significant and large positive correlations with between-subject variance and negative correlations with error variance across a broad range of performance measures, and these correlations remained relatively stable across sample sizes. However, ICCs were either weakly or non-significantly correlated with within-subject variance, a finding that also remained relatively stable across sample sizes. These results suggest that ICCs may be insufficient for discriminating reliability, particularly for within-subject variance. Lastly, we demonstrate that increasing noise between data from sessions 1 and 2 increases the critical *point of stability*, meaning that data from more participants are required before stable estimates of variance components are reached. Therefore, studies of reliability should ensure they use variance decomposition methods alongside larger sample sizes to more informatively measure the reliability of task performance.

The results presented here highlight the importance of sample size considerations for test–retest reliability work, and support existing work indicating that greater sample sizes than are often used are required for reliable (individual differences) research (Button et al., 2013; de Winter et al., 2016; Hedge et al., 2018; Hirschfeld et al., 2014; Kretzschmar & Gignac, 2019; Marek et al., 2022; Paccagnella, 2011; Schönbrodt & Perugini, 2013). For instance, Schönbrodt and Perugini (2013) suggest there are few scenarios where personality psychology using correlational methods should justify sample sizes smaller than 150, while Marek et al. (2022) and Gell et al. (2023) suggest that thousands of participants may be required for reliable brain–behaviour associations, although this depends on the reliability and validity of the measures used (see also Spisak et al., 2023, for discussion).

A viable alternative to collecting large samples is to instead improve reliability by increasing the density of data collected within a set of individuals (Kraus et al., 2023; Smith & Little, 2018; Tiego et al., 2023). In precision research, each subject acts as their own replication unit, with a large amount of data collected within small/single-subject units. This may be particularly useful in situations where practical impediments, such as time and funding restrictions or specialist populations, would prevent collection of data from hundreds or thousands of individuals. This approach may also enhance the prediction of momentary factors that influence the rank order of a given data point. For instance, intensive longitudinal designs (Lydon-Staley et al., 2019) could be used to enhance estimates of both within- and between-subject effects. This would have the added benefit of providing insight into how momentary changes in cognitive and affective state influence behaviour and model parameter estimates, which are missed in large-*N* studies with a single time point, since temporal dynamics cannot be modelled. One relevant example of the utility of this approach comes from Schaaf et al. (2023), who found that the current state of an individual significantly influences their reward learning (using data from two time points). Yet, this is a nascent field of research, and few insights into temporal aspects and predictors of reward learning behaviour exist.

One important similarity between the work presented here and previous work looking at the reliability of reversal learning task performance (Schaaf et al., 2023; Waltmann et al., 2022) is the consistency of the best-fitting models despite differences in task structure. For instance, the best-fitting model of Waltmann et al. (2022) was the same as our best-fitting model here (dual update, single learning rate, and separate reinforcement sensitivity parameters for wins and losses). Similarly, although Schaaf et al. (2023) only fit models from the softmax family, our best-fitting model in the softmax family (dual update, separate learning rates for wins and losses, and an update discount weight for the unchosen option) matched the best-fitting model of both Schaaf et al. (2023) and Waltmann et al. (2022). This consistency suggests these models are useful for approximating latent processes underlying reversal learning. The commonality of counterfactual updates across all these models makes sense in the context of two-choice reversal learning, where only one of the two outcomes can be optimal at any given moment. Therefore, when an agent updates the expected value of an action based on an outcome, simultaneously updating the expected value of the unchosen action using a counterfactual outcome makes behaviour more responsive and able to rapidly adjust in response to a change, in line with Bayesian state inference approaches to reversal learning (Bartolo & Averbeck, 2020; Costa et al., 2015). With experience, this dual updating should reduce perseverative responding as the agent learns with greater fidelity when transitions in the optimal choice assignment occur. This assumption is supported by our finding of lower levels of reliability for both accuracy and perseveration between sessions (which typically increase and decrease, respectively, for subjects) versus other behavioural measures, and suggests that subjects get “better” at the task. It may also explain why we observed lower reliability for our learning rate parameter estimates relative to other model parameters, as the rate at which expected values are updated is refined through experience, while other parameters (e.g., reinforcement sensitivity) may be less experience-dependent.

Another similarity is that our work here shows that reliability, as assessed using ICCs, is broadly in line with previous work (Schaaf et al., 2023; Waltmann et al., 2022). For instance, our confidence intervals around ICC measures for our collected behavioural data overlapped with those presented by Waltmann et al. (2022) for all behavioural measures (except for lose–stay behaviour estimated from the joint session regression model) and parameter estimates from our best-fitting model using the EM approach (dual update, single learning rate, and separate reinforcement sensitivity parameters for wins and losses). Given our larger sample size and narrower confidence intervals relative to Waltmann et al. (2022), we suggest that our estimates of reliability presented here may be more representative of the true underlying reliability of reversal learning performance measures.

One important sample size-related consideration for test–retest reliability studies is ensuring they are sufficiently powered, and this could be achieved by using expected ICC values in power calculations. This approach is similar to the use of effect sizes for a priori sample size calculations. In the latter case, effect sizes from previously published work are used to identify the sample size required to achieve the given effect size at a particular level of statistical power. For ICCs, Doros and Lew (2010) present a method where the width of confidence intervals is used to estimate appropriate sample sizes for reliability studies.

While the work presented here provides valuable insight into the reliability of reversal learning task performance measures, there are several limitations worth mentioning. Firstly, it is important to highlight that these behavioural data were collected from an online sample. Although online data collection enables large amounts of data to be more readily collected, as researchers we are unable to control the environment in which each subject completed the reversal learning task. To mitigate this we included response checks in our task and questionnaire measures to identify and exclude subjects that were clearly inattentive. However, there may be nuanced and non-systematic differences in the behaviour of our subjects that influenced how reliable their performance was over the two testing sessions. To account for some of these challenges, future work could, as previously mentioned, collect data from the same subjects over a greater number of testing sessions or could use tools such as WebGazer (Papoutsaki et al., 2016) to track gaze directions for compliance monitoring.

A second limitation of the work presented here is that point estimates of model parameters across subjects were taken using the best-fitting model at the group level. However, the best-fitting model for a given subject will not necessarily be the same as that for the group, and alternative approaches to model fitting can be used to infer the best-fitting model at the level of both the subject and the group (Piray & Daw, 2020; Piray et al., 2019; Williams & Christakou, 2022). Coupling individualised model fits with momentary measures of individual state could, again, improve the explanatory power of model parameters.

Finally, it may be the case that task performance or within-subject variance changes as subjects gain further experience on the task. Although we provide subjects with explicit instruction about the task’s general structure, such as one choice is better than the other and that the better choice will change throughout the task, subjects may still be refining their understanding of aspects of the task structure (the statistical relationships between actions and outcomes, their time estimate since last reversal, etc.), even after two sessions. Therefore, measuring reliability while subjects are still learning these representations will place an upper bound on reliability, and will be dependent on how quickly a stable representation of the task environment is generated, which in turn will vary between individuals. In the future, it may be worth considering how reliable performance is after sufficient overtraining on a given task.

In summary, we assessed the reliability of reversal learning behaviour using data collected from a large online sample and found good reliability of behavioural and computational model parameters at the group level, in line with findings from previous literature. However, our results also suggest that while behaviour may appear stable at the group level based on ICC values, sample size contributes significantly to variability in the estimates of variance components that underlie ICCs. Moreover, associations between estimates of variance components and calculated ICC values appear to remain relatively stable across sample sizes, with between-subject variance being highly positively and error variance being highly negatively correlated, but within-subject variance being weakly or non-significantly correlated with ICC values. This effect for within-subject variance challenges traditional practices in assessing test–retest reliability, and demonstrates the importance of understanding individual factors that contribute to (un)reliability. For instance, within-subject variance could be due to momentary differences in cognition and/or affect, and future work should aim to address how momentary state influences behaviour. These results also hold for the effects of sample size on estimates of computational modelling parameters, suggesting further characterisation of the stability of model parameters within and across tasks over time is needed before point estimates of parameter values can be considered stable trait-like measures.

## Supplementary Information

Below is the link to the electronic supplementary material.

Supplementary file1 (DOCX 9440 KB)

## Data Availability

Data and associated analysis code for the current study are available at the University of Reading Research Data Archive 10.17864/1947.001377.

## Code availability

Data and associated analysis code for the current study are available at the University of Reading Research Data Archive 10.17864/1947.001377.

## Declarations

### Open practices statement

Data and associated analysis code for the current study are available at the University of Reading Research Data Archive 10.17864/1947.001377. None of the experiments were preregistered.
