# Supplementary material for: Sample size matters when estimating test–retest reliability of behaviour
Source: Behav Res Methods. 2025 Mar 21;57(4):123. doi: 10.3758/s13428-025-02599-1 (PMC11928395; doi:10.3758/s13428-025-02599-1)
Supplement: Supplementary file 1 — Supplementary file1 (DOCX 9440 KB) [file 13428_2025_2599_MOESM1_ESM.docx]

# Supplementary Methods

## Participants

Participants were enrolled in this study using the online recruitment platform Prolific (https://www.prolific.co/) in two waves (wave 1: August – September 2021; wave 2: March – April 2022). Ineligible participants were filtered out from having our study advertised to them using Prolific’s participant filters. The following filters were applied: Medication use – No; NHS Mental Health Support – No; Autism Spectrum Disorder – No; Mild cognitive impairment/Dementia – No; Mental illness daily impact – No; Mental health/illness/condition – ongoing – No; Approval Rate – 100 minimum, 100 maximum; Deception – Yes; Other crowdsourcing platforms – No; Smoking status – I have never smoked (smoked fewer than 100 cigarettes in my lifetime); smoking: Tobacco or e-cigarettes – Do not use e-cigarettes or tobacco products; Vaping status – I am a non-vaper (vaped fewer than 20 times in my lifetime). Using these filters identified 2,997 eligible participants who had been active over the past 90 days. During consent, participants confirmed whether they had any clinically diagnosed mental health disorders, whether they smoked, and if they took any psychoactive medication or illicit drugs. Participants were required to complete this study using either a laptop or desktop personal computer, due to the need of a keyboard to record keypresses during the reversal learning task. 257 participants recruited through Prolific consented to take part in the study. 251 participants completed the experiment in the first part of the study. 2 participants were excluded after the first phase because they failed instructional attention check questions, and 14 were excluded because they failed nonsensical attention check questions or careless / insufficient effort (C/IE) responding checks (described further below) in the reversal learning task. 222 participants who were eligible for the second part of the study completed the experiment. 1 participant was excluded after the second phase because they failed instructional attention check questions, and 8 were excluded because they failed nonsensical attention check questions or the C/IE responding checks.

Participants who successfully completed the first phase of the study were reimbursed £1.75 for their time. This payment was framed as a basic pay rate of £1.25, plus an additional 50p that would be awarded based on their performance in the reversal learning task. This was done to maximise the likelihood that participants would remain focused while completing the task. Participants who completed the first phase of the study but failed the nonsensical attention check or the C/IE responding checks were reimbursed £1.25 for their time. Participants who failed the instructional attention check, or who did not complete their submission were not reimbursed, per participation rules on Prolific.

Participants who successfully completed the second phase of the study were reimbursed £2.50 for their time. As in the first phase, this payment was framed as a basic rate of £1.25, plus a performance bonus of £1.25. This payment bonus was larger during the second phase of the study than the first to encourage participants to complete both parts of the study. Participants who completed the second phase of the study but failed the nonsensical attention check or the C/IE responding checks were reimbursed £1.25 for their time. Participants who failed instructional attention checks, or who did not complete their submission for the second phase were not reimbursed, per participation rules on Prolific.

We used a binomial test to identify participants performing at or below chance level, and excluded them from further analyses (Zorowitz et al., 2023). Our sample used for statistical analysis includes 150 participants (mean age = 35.450, SD = 13.295, range = 19-73, female = 97). This study was approved by the ethics committee of the School of Psychology and Clinical Language Sciences, University of Reading (2021-50-AC).

## Reversal learning task

### Careless / Insufficient Effort Responding Checks

The reversal learning task included automated careless / insufficient effort (C/IE) responding checks that terminated the task prematurely if met. These conditions were 1. Not making a valid choice over five consecutive trials or 2. Not making a valid choice for over 5% of the total number of trials. As part of the study, participants completed the 12 item version of the Intolerance of Uncertainty Scale (Carleton et al., 2007); to check for C/IE, we added two instructional attention check questions (“*I am certain that I have read these questions carefully. Press two as your response.*”, and “*Worrying about good quality data is something that we as experimenters do. Therefore, we ask that you please select five as your response for this question.*”) and one infrequency attention check question (“*I have spent lots of time over the last two weeks worrying about the 1977 Olympics*” – this question is nonsensical since no Olympics were held in 1977) (Huang et al., 2015; Zorowitz et al., 2023). The latter infrequency question was included since this follows best practices for using nonsensical attentional checks in online research (Huang et al., 2015; Zorowitz et al., 2023). Participants were made explicitly aware of the use of attentional checks, and that their responses to the Intolerance of Uncertainty Scale questions would not influence their bonus payment. The intolerance of uncertainty scale was hosted on the online platform Gorilla (<https://gorilla.sc>).

Participants who passed our built-in C/IE responding checks in the reversal learning task and the intolerance of uncertainty questionnaire had their behavioural data screened again. We identified potential C/IE participants using the following performance metrics: responding using a single key for > 2/3 of all trials; responding to one stimulus for > 2/3 of all trials; reaction time < 250m/s on 10% or more trials; and accuracy (choice of correct option regardless of outcome) significantly worse than chance, based on a binomial test. These criteria are based on previously described C/IE criteria for an online reversal learning task (Zorowitz et al., 2023). Behavioural data for the remaining participants were screened manually by BW and LF, but no participants were removed because there was no conclusive evidence to determine whether they were C/IE responders or poor learners.

## Computational Modelling

### Models

The first model in the softmax family is a model-free reinforcement learning model with a single learning rate parameter ($\alpha$) and inverse temperature parameter ($\beta$). In this model the expected value ($V$) of choice $k$ on trial $t$ ($V_{t}^{k}$) is updated for the next trial ($t+1$) by adding the product of the learning rate and the prediction error $(\lambda_{t}-V_{t}^{k})$, which is the difference between the actual ($\lambda$) and expected value (eq. 1).

$$\begin{aligned} V_{t+1}^{k}=V_{t}^{k}+\alpha\left( \lambda_{t}-V_{t}^{k} \right) \#\left( 1 \right) \end{aligned}$$

The probability of making choice $k$ on trial $t$ is determined by the softmax choice rule (eq. 2), and the inverse temperature parameter ($\beta$) determines the extent to which choices are based on expected value estimates. When $\beta=0$, choices would be made completely at random; when $\beta=\infty$ the choice with the largest expected value would be deterministically chosen.

$$\begin{aligned} p_{t}^{k}=\frac{e^{\beta V_{t}^{k}}}{\sum_{i=1}^{K} e^{\beta V_{t}^{i}}} \#\left( 2 \right) \end{aligned}$$

In model one, expected values are updated at the same rate for positive and negative prediction errors. However, there is evidence that suggests that positive and negative prediction errors have asymmetric update rates with different sensitivities to wins and losses (Niv et al., 2012). Therefore, in model two included separate learning rates for wins and losses (eq. 3).

$$\begin{aligned} V_{t+1}^{k}=V_{t}^{k}+\alpha^{win/loss}\left( \lambda_{t}-V_{t}^{k} \right)\#\left( 3 \right) \end{aligned}$$

In models three and four, because choices may have dissociable sensitivities for previous wins and losses, separate inverse temperature parameters are used based on whether a win or loss was experienced on the previous trial (eq. 4); model three used a single learning rate for updating expected value (eq. 1) and model three used dual learning rates (eq. 3).

$$\begin{aligned} p_{t}^{k}=\frac{e^{\beta_{win/loss}V_{t}^{k}}}{\sum_{i=1}^{K} e^{\beta_{win/loss}V_{t}^{i}}} \#\left( 4 \right) \end{aligned}$$

Models five to eight updated the expected value choice $k$ on trial $t$ with single (models five and seven) or dual learning rates (models six and eight) and had single (models five and six) or dual (models seven and eight) inverse temperature parameters. However, expected values for the unchosen options ($k_{unchosen}$) on trial $t$ were also updated using the inverse of the actual ($\lambda$) outcome on trial $t$ (eq. 5 for models five and seven; eq. 6 for models six and eight).

$$\begin{aligned} V_{t+1}^{k_{unchosen}}=V_{t}^{k_{unchosen}}+\alpha\left( \left( -\lambda_{t} \right)-V_{t}^{k_{unchosen}} \right)\#\left( 5 \right) \end{aligned}$$

$$\begin{aligned} V_{t+1}^{k_{unchosen}}=V_{t}^{k_{unchosen}}+\alpha^{+/-}\left( \left( -\lambda_{t} \right)-V_{t}^{k_{unchosen}} \right)\#\left( 6 \right) \end{aligned}$$

Lastly, models nine to twelve updated chosen ($k$) choices on trial $t$ as in models five to eight (respectively), however the update of unchosen ($k_{unchosen}$) options was weighted by a discount parameter ($\kappa$) for models nine and eleven (eq. 7) and ten and twelve (eq. 8) respectively.

$$\begin{aligned} V_{t+1}^{k_{unchosen}}=V_{t}^{k_{unchosen}}+\kappa\alpha\left( \left( -\lambda_{t} \right)-V_{t}^{k_{unchosen}} \right)\#\left( 7 \right) \end{aligned}$$

$$\begin{aligned} V_{t+1}^{k_{unchosen}}=V_{t}^{k_{unchosen}}+\kappa\alpha^{win/loss}\left( \left( -\lambda_{t} \right)-V_{t}^{k_{unchosen}} \right)\#\left( 8 \right) \end{aligned}$$

For the reinforcement sensitivity family of models there is no inverse temperature parameter included in the softmax choice rule (eq. 9). Instead, a reinforcement sensitivity ($\rho$) parameter is used. In the softmax family of models the inverse temperature parameter determines choice stochasticity by determining the extent to which choices are driven by expected values, while the reinforcement sensitivity parameter does this by determining the maximum difference between expected values, which places a lower bound on choice stochasticity (Waltmann et al., 2022).

$$\begin{aligned} p_{t}^{k}=\frac{e^{V_{t}^{k}}}{\sum_{i=1}^{K} e^{V_{t}^{i}}} \#\left( 9 \right) \end{aligned}$$

The reinforcement sensitivity family of models broadly follow the softmax family of models with respect to how expected values are updated. The first model in the reinforcement sensitivity family is a model-free reinforcement learning model with a single learning rate parameter ($\alpha$) and reinforcement sensitivity parameter ($\rho$). When calculating a prediction error on trial $t$ in the reinforcement sensitivity family of models the actual outcome is scaled by the reinforcement sensitivity parameter ($\rho\lambda_{t}$) before subtracting the expected value ($V$) of choice $k$ on trial $t$ ($V_{t}^{k}$). Expected values are updated for the next trial ($t+1$) by adding the product of the learning rate and the prediction error $(\rho\lambda_{t}-V_{t}^{k})$ (eq. 10).

$$\begin{aligned} V_{t+1}^{k}=V_{t}^{k}+\alpha\left( \rho\lambda_{t}-V_{t}^{k} \right) \#\left( 10 \right) \end{aligned}$$

The second model in the reinforcement sensitivity family had separate learning rates for wins and losses (eq. 11). The third and fourth models included separate reinforcement sensitivities for wins and losses, coupled with symmetric (eq. 12) and asymmetric (eq. 13) learning rates for wins and losses, respectively.

$$\begin{aligned} V_{t+1}^{k}=V_{t}^{k}+\alpha^{win/loss}\left( \rho\lambda_{t}-V_{t}^{k} \right)\#\left( 11 \right) \end{aligned}$$

$$\begin{aligned} V_{t+1}^{k}=V_{t}^{k}+\alpha\left( \rho_{win/loss}\lambda_{t}-V_{t}^{k} \right)\#\left( 12 \right) \end{aligned}$$

$$\begin{aligned} V_{t+1}^{k}=V_{t}^{k}+\alpha^{win/loss}\left( \rho_{win/loss}\lambda_{t}-V_{t}^{k} \right)\#\left( 13 \right) \end{aligned}$$

Models five to eight updated expected values for both chosen and unchosen choices on trial $t$. The expected value of choice $k$ on trial $t$ was updated using a single (models five and seven) or separate learning rates (models six and eight). Models five and six included a single reinforcement sensitivity parameter, while models seven and eight included separate reinforcement sensitivities for wins and losses. Expected values for unchosen options ($k_{unchosen}$) on trial $t$ were also updated using the inverse of the actual ($\lambda$) outcome on trial $t$ (eqs. 14 and 16 for models five and seven; eqs. 15 and 17 for models six and eight).

$$\begin{aligned} V_{t+1}^{k_{unchosen}}=V_{t}^{k_{unchosen}}+\alpha\left( \rho\left( {-\lambda}_{t} \right)-V_{t}^{k_{unchosen}} \right)\#\left( 14 \right) \end{aligned}$$

$$\begin{aligned} V_{t+1}^{k_{unchosen}}=V_{t}^{k_{unchosen}}+\alpha^{win/loss}\left( \rho\left( {-\lambda}_{t} \right)-V_{t}^{k_{unchosen}} \right)\# \left( 15 \right) \end{aligned}$$

$$\begin{aligned} V_{t+1}^{k_{unchosen}}=V_{t}^{k_{unchosen}}+\alpha\left( \rho_{win/loss}\left( {-\lambda}_{t} \right)-V_{t}^{k_{unchosen}} \right)\#\left( 16 \right) \end{aligned}$$

$$\begin{aligned} V_{t+1}^{k_{unchosen}}=V_{t}^{k_{unchosen}}+\alpha^{win/loss}\left( \rho_{win/loss}\left( {-\lambda}_{t} \right)-V_{t}^{k_{unchosen}} \right)\#\left( 17 \right) \end{aligned}$$

Lastly, models nine to twelve updated chosen ($k$) choices on trial $t$ as in models five to eight (respectively), however the update of unchosen ($k_{unchosen}$) options was weighted by a discount parameter ($\kappa$) for models nine and eleven (eqs. 18 and 20) and ten and twelve (eqs. 19 and 21) respectively.

$$\begin{aligned} V_{t+1}^{k_{unchosen}}=V_{t}^{k_{unchosen}}+\kappa\alpha\left( \rho\left( {-\lambda}_{t} \right)-V_{t}^{k_{unchosen}} \right)\#\left( 18 \right) \end{aligned}$$

$$\begin{aligned} V_{t+1}^{k_{unchosen}}=V_{t}^{k_{unchosen}}+{\kappa\alpha}^{win/loss}\left( \rho\left( {-\lambda}_{t} \right)-V_{t}^{k_{unchosen}} \right)\#\left( 19 \right) \end{aligned}$$

$$\begin{aligned} V_{t+1}^{k_{unchosen}}=V_{t}^{k_{unchosen}}+\kappa\alpha\left( \rho_{win/loss}\left( {-\lambda}_{t} \right)-V_{t}^{k_{unchosen}} \right)\#\left( 20 \right) \end{aligned}$$

$$\begin{aligned} V_{t+1}^{k_{unchosen}}=V_{t}^{k_{unchosen}}+\kappa\alpha^{win/loss}\left( \rho_{win/loss}\left( {-\lambda}_{t} \right)-V_{t}^{k_{unchosen}} \right)\#\left( 21 \right) \end{aligned}$$

## Reliability assessment – replication of Waltmann et al. (2022)

We used four estimation methods for calculating the reliability of behavioural measures: (1) Intra-class correlation coefficients (ICCs) of mean measures of raw scores between the two sessions; (2) ICCs and Pearson’s correlations of marginal means from separate regression models for each session; (3) ICCs and Pearson’s correlations of marginal means for each session from a joint regression model, pooling the data from two sessions, explicitly modelling the effect of session; and (4) model-derived ICCs of variance metrics from the joint regression model explicitly modelling the effect of sessions. For the best fitting model from each model family, we used two methods of calculating the reliability of parameter estimates across the three model fitting approaches: (1) ICCs and Pearson’s correlation coefficients of model parameters estimated separately for each session; (2) ICCs and Pearson’s correlation coefficients of model parameters estimated for each session, but by fitting the model jointly on data pooled from the two sessions (to account for within subject covariance across sessions).

The ICCs used to assess the reliability of behavioural measures and computational modelling parameters were ICC(A,1), and ICC(1). ICC(A,1) is used in reliability studies where time is a factor, and is a two-way mixed, single-measure, absolute-agreement metric. This means that for a given measure the ICC represents the absolute difference between timepoints, and does not aim to generalise this difference between timepoints. ICC(1) is a one-way random, single-measure metric, where time is considered a random effect. ICC and correlation coefficients were interpreted following guidelines from Cicchetti (1994; but also see Gell et al., (2023) for an overview of how even a small change in reliability can affect accuracy), with poor: ICC < 0.4; fair: 0.4 ≤ ICC < 0.6; good: 0.6 ≤ ICC < 0.75; excellent: 0.75 ≤ ICC.

We calculated within-subject, between-subject, and error variance components for behavioural measures of performance based on: (1) mean values, (2) marginal means estimated using regression analysis with sessions modelled separately, (3) marginal means estimated using regression analysis with sessions modelled together, (4) between-subject and error variance components calculated directly from the regression model with sessions modelled together (Bartko, 1966; McGraw & Wong, 1996). Variance components were then normalised so they summed to one, in line with the approach used by Waltmann et al. (2022).

# Supplementary results

## Behavioural measures of performance – reliability

As in Waltmann et al. (2022), we used intra-class correlation coefficients, and correlation coefficients to quantify the reliability of behavioural metrics of performance, using mean values, estimated using marginal means from regression analyses modelling sessions separately and jointly, and using direct estimations from regression model (Figure 1). We also used the confidence intervals reported by Waltmann et al. (2022) to confirm whether our estimates fell within their confidence intervals and vice versa (see supplementary Table 1).

Reliability estimates for accuracy were poor when using mean values (ICC(A,1)=0.37, ICC(1)=0.33), and poor to fair (ICC(A,1)=0.33, ICC(1)=0.25, r=0.42) when estimated separately for sessions, but were fair to good when jointly estimated for sessions (ICC(A,1)=0.61, ICC(1)=0.59, r=0.65). Model calculated estimates for accuracy were fair (ICC(1)=0.47).

Staying behaviour overall showed good reliability, both when using mean values (ICC(A,1)=0.69, ICC(1)=0.68), when sessions were modelled separately (ICC(A,1)=0.62, ICC(1)=0.6, r=0.67), jointly, (ICC(A,1)=0.69, ICC(1)=0.69, r=0.74), and when calculated directly from the model (ICC(1)=0.68). Similar or better reliability was found for staying behaviour specifically after losses, where estimates ranged from good to excellent when using mean values (ICC(A,1)=0.73, ICC(1)=0.73), modelling sessions separately (ICC(A,1)=0.73, ICC(1)=0.73, r=0.75), jointly (ICC(A,1)=0.82, ICC(1)=0.82, r=0.83), and calculated directly from the model (ICC(1)=0.78), but not for staying behaviour after wins where reliability was fair when using mean values (ICC(A,1)=0.49, ICC(1)=0.47), and when sessions were modelled separately (ICC(A,1)=0.47, ICC(1)=0.45, r=0.55), and fair to good for sessions modelled jointly (ICC(A,1)=0.6, ICC(1)=0.58, r=0.66), and calculated directly from the model (ICC(1)=0.61). Perseverative behaviour had poor reliability when calculated for mean values (ICC(A,1)=0.38, ICC(1)=0.35), and poor to fair reliability when estimated separately (ICC(A,1)=0.36, ICC(1)=0.32, r=0.41), jointly, (ICC(A,1)=0.51, ICC(1)=0.5, r=0.55), and when calculated directly from the model (ICC(1)=0.46).

Reaction times overall showed good to excellent reliability, both when using mean values (ICC(A,1)=0.75, ICC(1)=0.74) and when sessions were modelled separately (ICC(A,1)=0.75, ICC(1)=0.74, r=0.8), and excellent reliability when sessions were modelled jointly, (ICC(A,1)=0.78, ICC(1)=0.77, r=0.82), and when calculated directly from the model (ICC(1)=0.76). The same pattern of results were found for reaction times after wins for mean values (ICC(A,1)=0.76, ICC(1)=0.75), when sessions were modelled separately (ICC(A,1)=0.7, ICC(1)=0.76, r=0.8), jointly, (ICC(A,1)=0.8, ICC(1)=0.8, r=0.83), and when calculated directly from the model (ICC(1)=0.78). A similar pattern was found for reaction times after losses, with reliabilities ranging from good to excellent for mean values (ICC(A,1)=0.71, ICC(1)=0.7), when sessions were modelled separately (ICC(A,1)=0.77, ICC(1)=0.7, r=0.79), jointly, (ICC(A,1)=0.75, ICC(1)=0.74, r=0.81), and when calculated directly from the model (ICC(1)=0.72). Reliability for the difference in reaction times between wins and losses was poor for mean values (ICC(A,1)=0.38, ICC(1)=0.37), poor to fair when sessions were modelled separately (ICC(A,1)=0.37, ICC(1)=0.33, r=0.42), and when calculated directly from the model (ICC(1)=0.55), but were good when sessions were modelled jointly (ICC(A,1)=0.68, ICC(1)=0.67, r=0.72).

We used split-half reliability to calcualte the reliability of behaviour within sessions, by comparing correlation coefficeints (with and without Spearman-Brown correction) for odd and even numbered trials (Figure 2). Reliabilities ranged from good to excellent for all behavioural metrics in session one, except for the difference in reaction times for wins and losses (accuracy: r=0.72, r(SB)=0.83; stay overall : r=0.8, r(SB)=0.89; staying after wins: r=0.79, r(SB)=0.88; staying after losses: r=0.86, r(SB)=0.92; perseveration: r=0.8, r(SB)=0.89; reaction time: r=0.97, r(SB)=0.98; reaction time for wins: r=0.97, r(SB)=0.99; reaction time for losses: r=0.96, r(SB)=0.98; difference in reaction times: r=0.57, r(SB)=0.73). The same patter of reliabilties was also present for behavioural metrics in session two (accuracy: r=0.76, r(SB)=0.87; stay overall : r=0.82, r(SB)=0.9; staying after wins: r=0.74, r(SB)=0.85; staying after losses: r=0.87, r(SB)=0.93; perseveration: r=0.83, r(SB)=0.91; reaction time: r=0.97, r(SB)=0.99; reaction time for wins: r=0.97, r(SB)=0.99; reaction time for losses: r=0.95, r(SB)=0.98; difference in reaction times: r=0.43, r(SB)=0.6).

## Computational modelling of behaviour – reliability

### Model comparison

Comparisons of model fit were performed using each model’s integrated Bayesian information criterion (iBIC) from the EM fitting approach (Figure 3). The best fitting model for the softmax family was the dual update model with a discount weight for the unchosen option with separate learning rates plus softmax temperatures for wins and losses (DU-2β2ακ). The best fitting model for the reinforcement sensitivity family was the dual update model with separate reinforcement sensitivities for wins and losses plus a single learning rate parameter (DU-2ρα), though the discount weighted variant (DU-2ρακ), plus the unweighted and weighted discount models with separate learning rates and reinforcement sensitivities (DU-2ρ2α, DU-2ρ2ακ) had only marginally increased iBICs (ΔiBIC = 3, 8 and 27 for each model, respectively). The best fitting models for the softmax and reinforcement sensitivity families are in line with the findings from Waltmann et al. (2022).

### Parameter reliability

Overall, DU-2ρα the best fitting model was the best fitting model (Figure 3). This model had good to excellent reliability when the learning rate (ICC(A,1)=0.64, ICC(1)=0.63, r=0.68, r_model calculated_=0.65), and reinforcement sensitivity for win (ICC(A,1)=0.74, ICC(1)=0.73, r=0.81, r_model calculated_=0.75), loss (ICC(A,1)=0.82, ICC(1)=0.82, r=0.83, r_model calculated_=0.76) parameters were jointly modelled and fitted with EM. Modelling separately with EM, and both separately and jointly with MAP-0 produced parameters that showed poor to good reliability (Figure 4). For the best fitting model from the softmax family, fitting both separately and jointly with the EM and MAP-0 approach produced parameters where reliabilities ranged from poor to good (Figure 5). For the best fitting softmax and reinforcement sensitivity models, all parameters fitted using maximum likelihood showed poor reliability between sessions for both separate and joint modelling across all reliability measures.

## Measures of performance – variance components

As in Waltmann et al. (2022) we partitioned variance into within-subject (session), between-subject and error components for each of the behavioural measures of performance across each estimation approach for behavioural measures (Figure 6), and computational modelling parameters (Figure 7).

# Supplementary Tables

Table 1: Confidence interval details for behavioural performance data

| Metric | Modelling | ICC | Waltmann | Ours | Ours in Waltmann CI | Waltmann in our CI | Difference |
| --- | --- | --- | --- | --- | --- | --- | --- |
| Accuracy | Separate | ICC(A,1) | 0.42 | 0.33 | yes | yes | 0.09 |
|  |  | ICC(1) | 0.42 | 0.25 | yes | no | 0.17 |
|  |  | r | 0.41 | 0.42 | yes | yes | -0.01 |
|  | Joint | ICC(A,1) | 0.66 | 0.61 | yes | yes | 0.05 |
|  |  | ICC(1) | 0.66 | 0.59 | yes | yes | 0.07 |
|  |  | r | 0.65 | 0.65 | yes | yes | 0 |
|  | Model calculated | ICC(1) | 0.52 | 0.47 |  |  | 0.05 |
| Stay | Separate | ICC(A,1) | 0.72 | 0.62 | yes | yes | 0.1 |
|  |  | ICC(1) | 0.71 | 0.6 | yes | no | 0.11 |
|  |  | r | 0.77 | 0.67 | yes | no | 0.1 |
|  | Joint | ICC(A,1) | 0.83 | 0.69 | yes | no | 0.14 |
|  |  | ICC(1) | 0.83 | 0.69 | no | no | 0.14 |
|  |  | r | 0.87 | 0.74 | no | no | 0.13 |
|  | Model calculated | ICC(1) | 0.83 | 0.68 |  |  | 0.15 |
| Win:Stay | Separate | ICC(A,1) | 0.59 | 0.47 | yes | yes | 0.12 |
|  |  | ICC(1) | 0.57 | 0.45 | yes | yes | 0.12 |
|  |  | r | 0.72 | 0.55 | yes | no | 0.17 |
|  | Joint | ICC(A,1) | 0.77 | 0.6 | yes | no | 0.17 |
|  |  | ICC(1) | 0.76 | 0.58 | no | no | 0.18 |
|  |  | r | 0.84 | 0.66 | no | no | 0.18 |
|  | Model calculated | ICC(1) | 0.69 | 0.61 |  |  | 0.08 |
| Lose:Stay | Separate | ICC(A,1) | 0.79 | 0.73 | yes | yes | 0.06 |
|  |  | ICC(1) | 0.79 | 0.73 | yes | yes | 0.06 |
|  |  | r | 0.79 | 0.75 | yes | yes | 0.04 |
|  | Joint | ICC(A,1) | 0.97 | 0.82 | no | no | 0.15 |
|  |  | ICC(1) | 0.97 | 0.82 | no | no | 0.15 |
|  |  | r | 0.97 | 0.83 | no | no | 0.14 |
|  | Model calculated | ICC(1) | 0.92 | 0.78 |  |  | 0.14 |
| Perseveration | Separate | ICC(A,1) | 0.25 | 0.36 | yes | yes | -0.11 |
|  |  | ICC(1) | 0.2 | 0.32 | yes | yes | -0.12 |
|  |  | r | 0.28 | 0.41 | yes | yes | -0.13 |
|  | Joint | ICC(A,1) | 0.72 | 0.51 | no | no | 0.21 |
|  |  | ICC(1) | 0.72 | 0.5 | no | no | 0.22 |
|  |  | r | 0.72 | 0.55 | yes | no | 0.17 |
|  | Model calculated | ICC(1) | 0.71 | 0.46 |  |  | 0.25 |
| RT | Separate | ICC(A,1) | 0.74 | 0.75 | yes | yes | -0.01 |
|  |  | ICC(1) | 0.72 | 0.74 | yes | yes | -0.02 |
|  |  | r | 0.84 | 0.8 | yes | yes | 0.04 |
|  | Joint | ICC(A,1) | 0.78 | 0.78 | yes | yes | 0 |
|  |  | ICC(1) | 0.76 | 0.77 | yes | yes | -0.01 |
|  |  | r | 0.86 | 0.82 | yes | yes | 0.04 |
|  | Model calculated | ICC(1) | 0.74 | 0.76 |  |  | -0.02 |
| Win:RT | Separate | ICC(A,1) | 0.66 | 0.7 | yes | yes | -0.04 |
|  |  | ICC(1) | 0.74 | 0.76 | yes | yes | -0.02 |
|  |  | r | 0.83 | 0.8 | yes | yes | 0.03 |
|  | Joint | ICC(A,1) | 0.79 | 0.8 | yes | yes | -0.01 |
|  |  | ICC(1) | 0.78 | 0.8 | yes | yes | -0.02 |
|  |  | r | 0.86 | 0.83 | yes | yes | 0.03 |
|  | Model calculated | ICC(1) | 0.76 | 0.78 |  |  | -0.02 |
| Lose:RT | Separate | ICC(A,1) | 0.77 | 0.77 | yes | yes | 0 |
|  |  | ICC(1) | 0.68 | 0.7 | yes | yes | -0.02 |
|  |  | r | 0.82 | 0.79 | yes | yes | 0.03 |
|  | Joint | ICC(A,1) | 0.75 | 0.75 | yes | yes | 0 |
|  |  | ICC(1) | 0.74 | 0.74 | yes | yes | 0 |
|  |  | r | 0.85 | 0.81 | yes | yes | 0.04 |
|  | Model calculated | ICC(1) | 0.71 | 0.72 |  |  | -0.01 |
| Win:RT-Loss:RT | Separate | ICC(A,1) | 0.45 | 0.37 | yes | yes | 0.08 |
|  |  | ICC(1) | 0.44 | 0.33 | yes | yes | 0.11 |
|  |  | r | 0.46 | 0.42 | yes | yes | 0.04 |
|  | Joint | ICC(A,1) | 0.75 | 0.68 | yes | yes | 0.07 |
|  |  | ICC(1) | 0.74 | 0.67 | yes | yes | 0.07 |
|  |  | r | 0.76 | 0.72 | yes | yes | 0.04 |
|  | Model calculated | ICC(1) | 0.76 | 0.55 |  |  | 0.21 |

Table 2: Confidence interval details for model parameter data

| Parameter | Fitting | Modelling | ICC | Waltmann | Ours | Ours in Waltmann CI | Waltmann in our CI | Difference |
| --- | --- | --- | --- | --- | --- | --- | --- | --- |
| Learning rate | ML | Separate | ICC(A,1) | 0.16 | 0.29 | yes | yes | -0.13 |
|  |  |  | ICC(1) | 0.17 | 0.29 | yes | yes | -0.12 |
|  |  |  | r | 0.16 | 0.3 | yes | no | -0.14 |
|  |  | Joint | ICC(A,1) | 0.17 | 0.1 | yes | yes | 0.07 |
|  |  |  | ICC(1) | 0.18 | 0.09 | yes | yes | 0.09 |
|  |  |  | r | 0.17 | 0.1 | yes | yes | 0.07 |
|  | MAP0 | Separate | ICC(A,1) | 0.2 | 0.25 | yes | yes | -0.05 |
|  |  |  | ICC(1) | 0.21 | 0.25 | yes | yes | -0.04 |
|  |  |  | r | 0.2 | 0.25 | yes | yes | -0.05 |
|  |  | Joint | ICC(A,1) | 0.2 | 0.25 | yes | yes | -0.05 |
|  |  |  | ICC(1) | 0.21 | 0.25 | yes | yes | -0.04 |
|  |  |  | r | 0.2 | 0.25 | yes | yes | -0.05 |
|  | EM | Separate | ICC(A,1) | 0.59 | 0.38 | yes | no | 0.21 |
|  |  |  | ICC(1) | 0.59 | 0.38 | yes | no | 0.21 |
|  |  |  | r | 0.6 | 0.4 | yes | no | 0.2 |
|  |  | Joint | ICC(A,1) | 0.83 | 0.64 | no | no | 0.19 |
|  |  |  | ICC(1) | 0.83 | 0.63 | no | no | 0.2 |
|  |  |  | r | 0.83 | 0.68 | no | no | 0.15 |
|  |  | Model calculated | ICC(1) | 0.74 | 0.65 |  |  | 0.09 |
| Reinforcement sensitivity for wins | ML | Separate | ICC(A,1) | -0.01 | -0.01 | yes | yes | 0 |
|  |  |  | ICC(1) | -0.01 | -0.02 | yes | yes | 0.01 |
|  |  |  | r | -0.03 | -0.01 | yes | yes | -0.02 |
|  |  | Joint | ICC(A,1) | -0.02 | 0 | yes | yes | -0.02 |
|  |  |  | ICC(1) | -0.01 | 0 | yes | yes | -0.01 |
|  |  |  | r | -0.02 | 0 | yes | yes | -0.02 |
|  | MAP0 | Separate | ICC(A,1) | 0.46 | 0.56 | yes | yes | -0.1 |
|  |  |  | ICC(1) | 0.45 | 0.54 | yes | yes | -0.09 |
|  |  |  | r | 0.48 | 0.6 | yes | no | -0.12 |
|  |  | Joint | ICC(A,1) | 0.46 | 0.56 | yes | yes | -0.1 |
|  |  |  | ICC(1) | 0.45 | 0.54 | yes | yes | -0.09 |
|  |  |  | r | 0.48 | 0.6 | yes | no | -0.12 |
|  | EM | Separate | ICC(A,1) | 0.64 | 0.56 | yes | yes | 0.08 |
|  |  |  | ICC(1) | 0.63 | 0.55 | yes | yes | 0.08 |
|  |  |  | r | 0.71 | 0.61 | yes | no | 0.1 |
|  |  | Joint | ICC(A,1) | 0.85 | 0.74 | yes | no | 0.11 |
|  |  |  | ICC(1) | 0.84 | 0.73 | yes | no | 0.11 |
|  |  |  | r | 0.93 | 0.81 | no | no | 0.12 |
|  |  | Model calculated | ICC(1) | 0.86 | 0.75 |  |  | 0.11 |
| Reinforcement sensitivity for losses | ML | Separate | ICC(A,1) | 0.2 | -0.01 | yes | no | 0.21 |
|  |  |  | ICC(1) | 0.2 | 0 | yes | no | 0.2 |
|  |  |  | r | 0.2 | -0.01 | yes | no | 0.21 |
|  |  | Joint | ICC(A,1) | 0.2 | 0 | yes | no | 0.2 |
|  |  |  | ICC(1) | 0.2 | 0 | yes | no | 0.2 |
|  |  |  | r | 0.2 | -0.01 | yes | no | 0.21 |
|  | MAP0 | Separate | ICC(A,1) | 0.52 | 0.48 | yes | yes | 0.04 |
|  |  |  | ICC(1) | 0.52 | 0.48 | yes | yes | 0.04 |
|  |  |  | r | 0.53 | 0.49 | yes | yes | 0.04 |
|  |  | Joint | ICC(A,1) | 0.52 | 0.48 | yes | yes | 0.04 |
|  |  |  | ICC(1) | 0.52 | 0.48 | yes | yes | 0.04 |
|  |  |  | r | 0.53 | 0.49 | yes | yes | 0.04 |
|  | EM | Separate | ICC(A,1) | 0.42 | 0.56 | yes | no | -0.14 |
|  |  |  | ICC(1) | 0.37 | 0.56 | yes | no | -0.19 |
|  |  |  | r | 0.53 | 0.56 | yes | yes | -0.03 |
|  |  | Joint | ICC(A,1) | 0.84 | 0.82 | yes | yes | 0.02 |
|  |  |  | ICC(1) | 0.84 | 0.82 | yes | yes | 0.02 |
|  |  |  | r | 0.94 | 0.83 | no | no | 0.11 |
|  |  | Model calculated | ICC(1) | 0.86 | 0.76 |  |  | 0.1 |

# Supplementary Figures


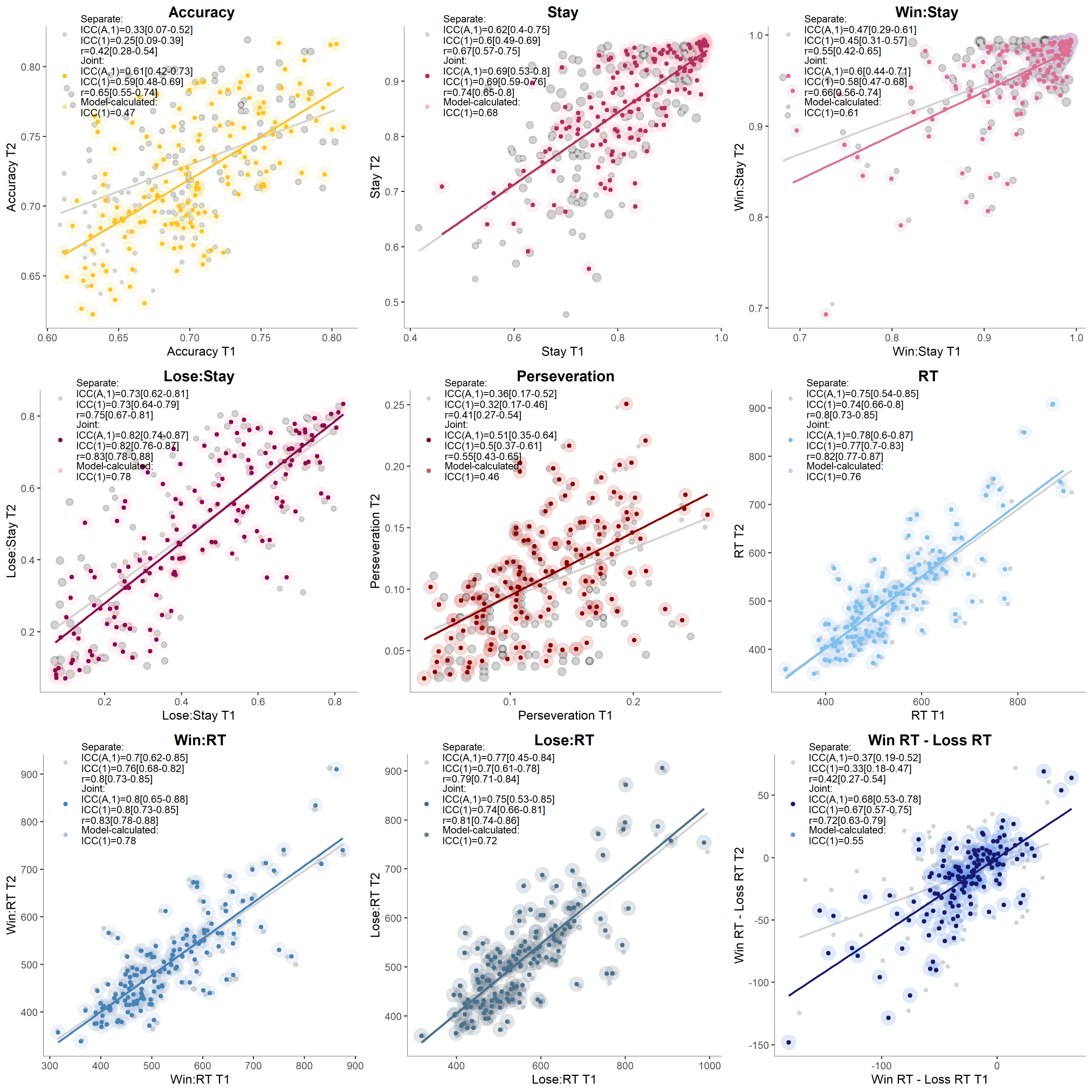


Figure 1: Reliability of behavioural task performance measures between sessions, as assessed using Intraclass Correlation Coefficients and correlations, with 95% confidence intervals given in square brackets. Coloured dots represent estimates of each behavioural measure from regression analyses that explicitly model the effect of session (joint models), and the shaded area around these coloured points is proportional to the standard error. Grey dots represent estimates of behavioural measures from different regression models for each session (separate models).


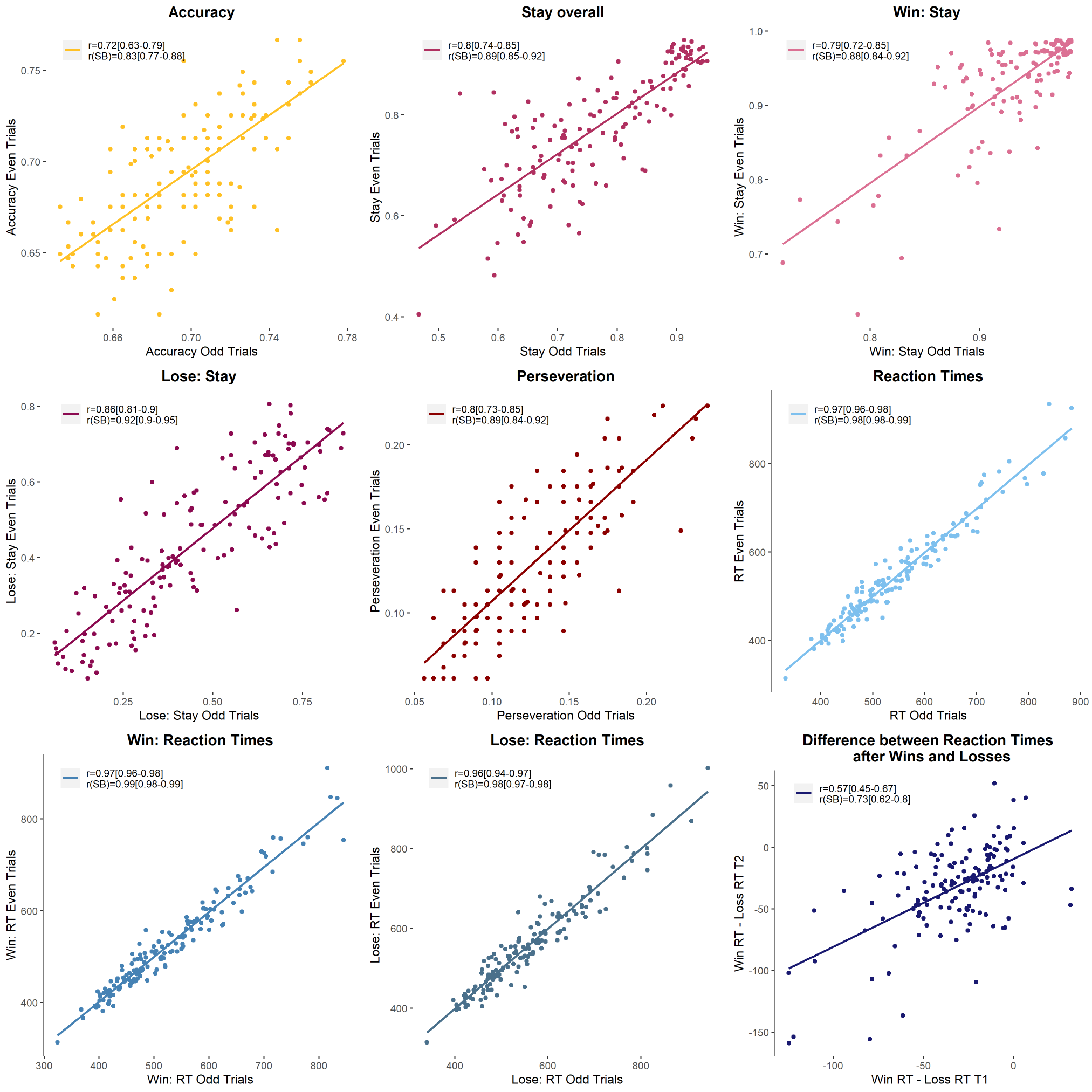


Figure 2: Split-half reliability of behavioural task performance measures in session one, as assessed using correlation coefficients (with and without Spearman-Brown correction), with 95% confidence intervals given in square brackets. Split-half reliability was calculated by comparing between even and odd trial numbers.


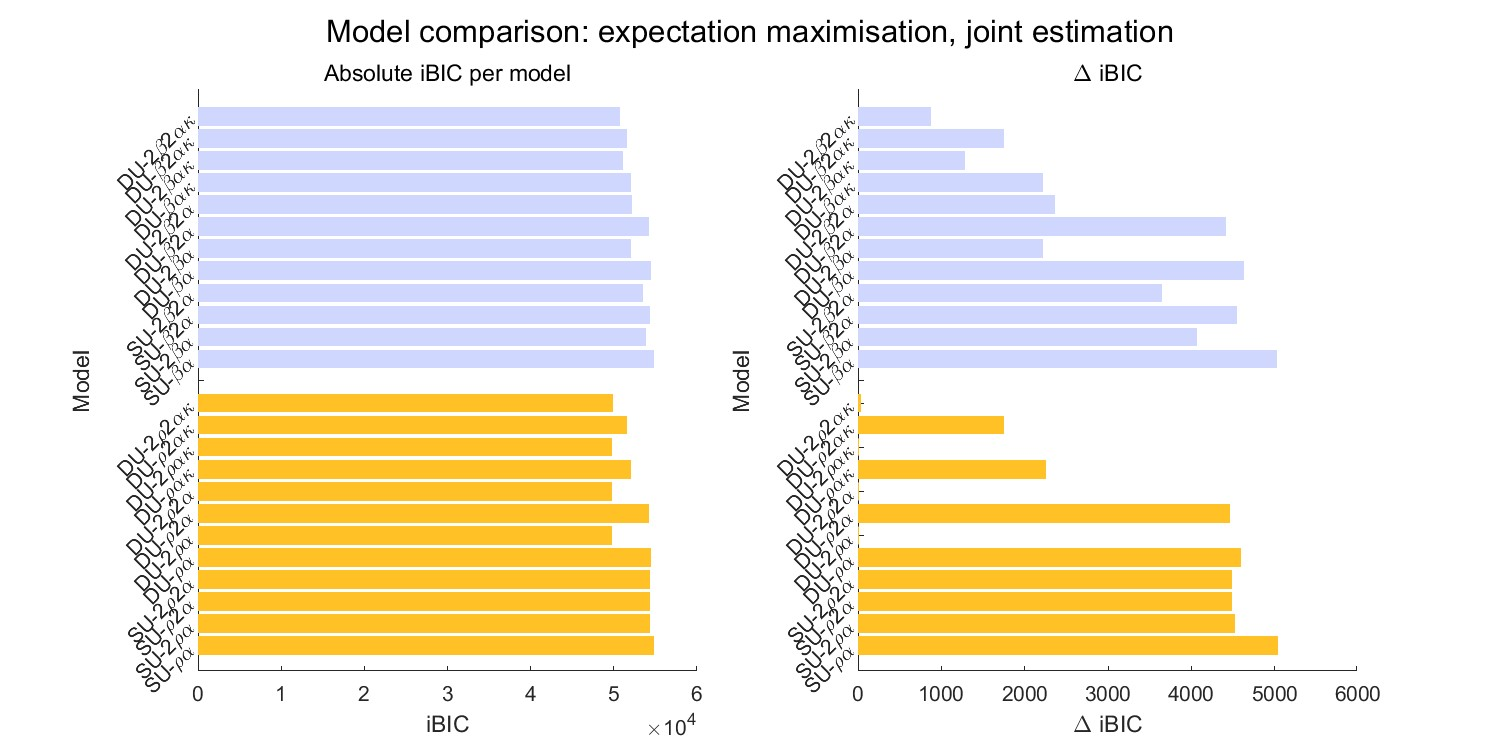


Figure 3: Model comparison was performed using the integrated Bayesian Information Criterion (iBIC), calculated for models fitted jointly for both sessions using maximum a posteriori estimation with (EM) informative priors. The best fitting model from the softmax family (blue bars) was a dual update model with dual learning rates dual inverse temperature parameters and a discount update weight for unchosen actions. The best fitting model for the reinforcement sensitivity family (yellow bars) was a dual update model with dual reinforcement sensitivity parameters and a single learning rate. SU single update, DU double update, 𝛽 softmax temperature, 𝛼 learning rate, 𝜅 double update weight, 𝜌 reinforcement sensitivity.


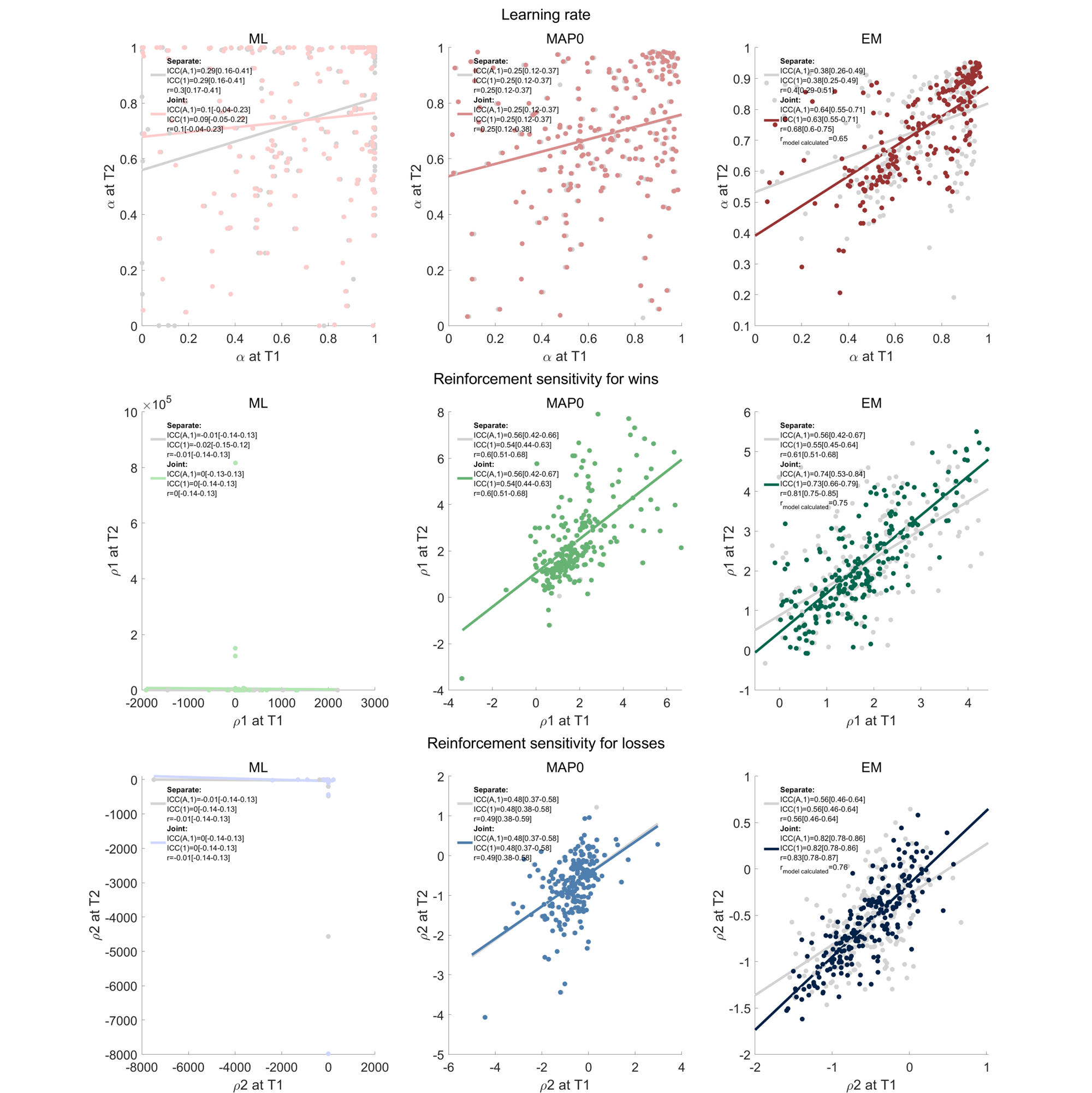


Figure 4: Reliability of computational modelling parameters between session for the best model in the reinforcement sensitivity family, as assessed using Intraclass correlation coefficients and correlations, with 95% confidence intervals given in square brackets. Reliability estimates were calculated for each of the three model fitting approaches used, namely maximum likelihood (ML), and maximum a posteriori estimation without (MAP0) and with (EM) informative priors. Further, models were fit separately or jointly for the two sessions. Coloured dots represent parameter estimates from joint modelling, and grey dots represent estimates from separate modelling.


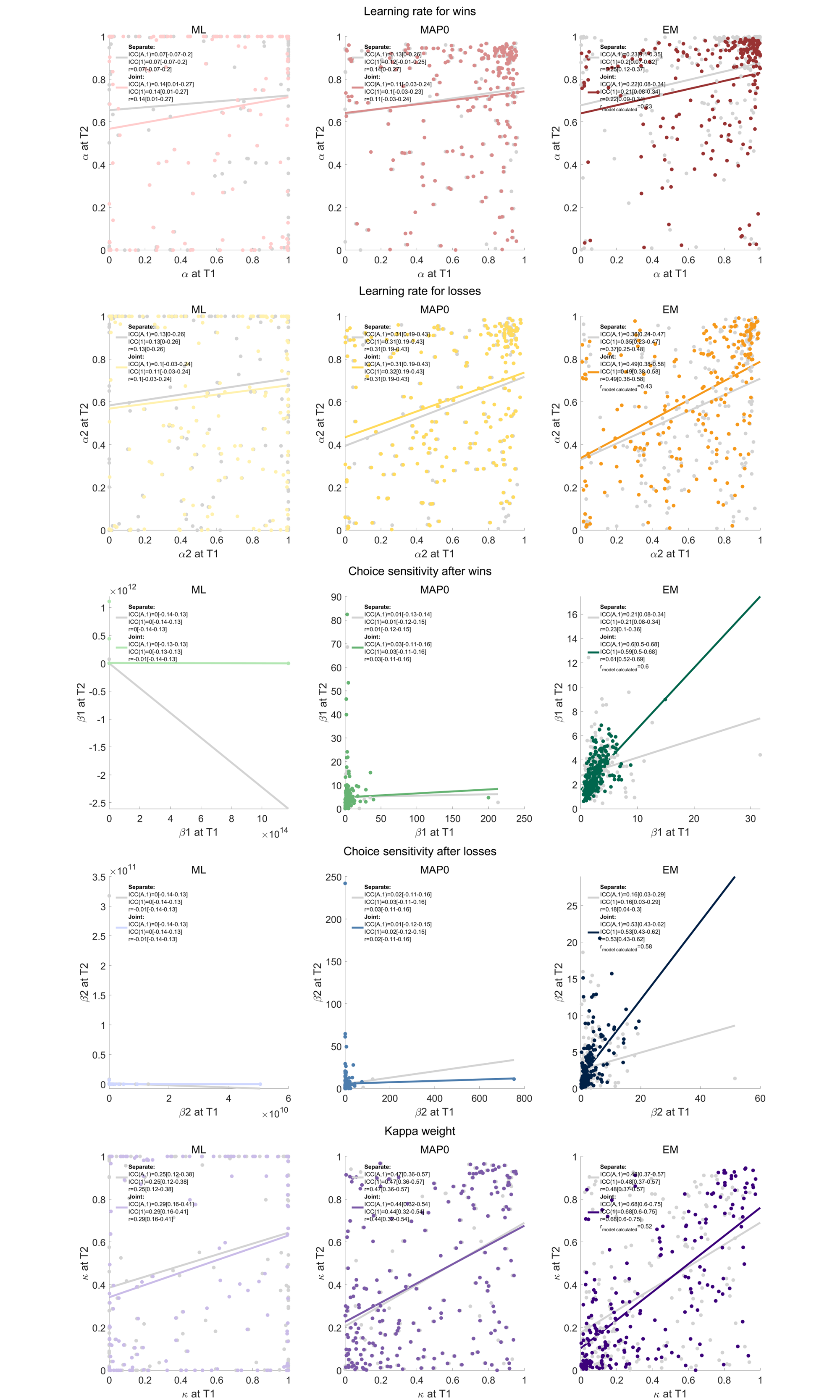


Figure 5: Reliability of computational modelling parameters between session for the best model in the softmax family, as assessed using Intraclass Correlation Coefficients and correlations, with 95% confidence intervals given in square brackets. Reliability estimates were calculated for each of the three model fitting approaches used, namely maximum likelihood (ML), and maximum a posteriori estimation without (MAP0) and with (EM) informative priors. Further, models were fit separately or jointly for the two sessions. Coloured dots represent parameter estimates from joint modelling, and grey dots represent estimates from separate modelling.


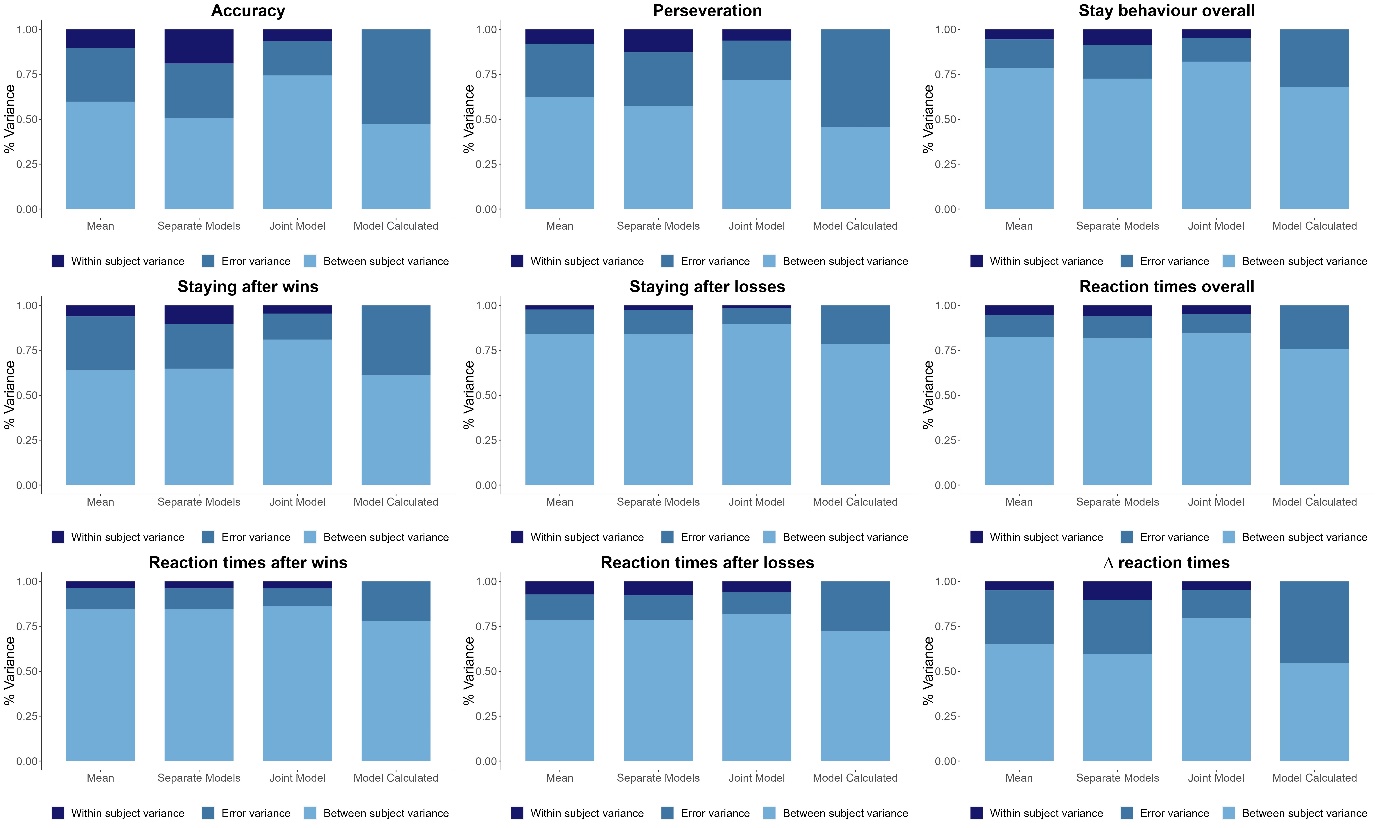


Figure 6: Within-subject, between-subject, and error variance proportions for each of our behavioural measures. Variance proportions were calculated based on mean measures, marginal means from separate regression models for each session, marginal means for each session from a joint regression model, and model-derived metrics from the joint regression model. For this last option, only variance proportions were calculated based on between-subject and error variance only.


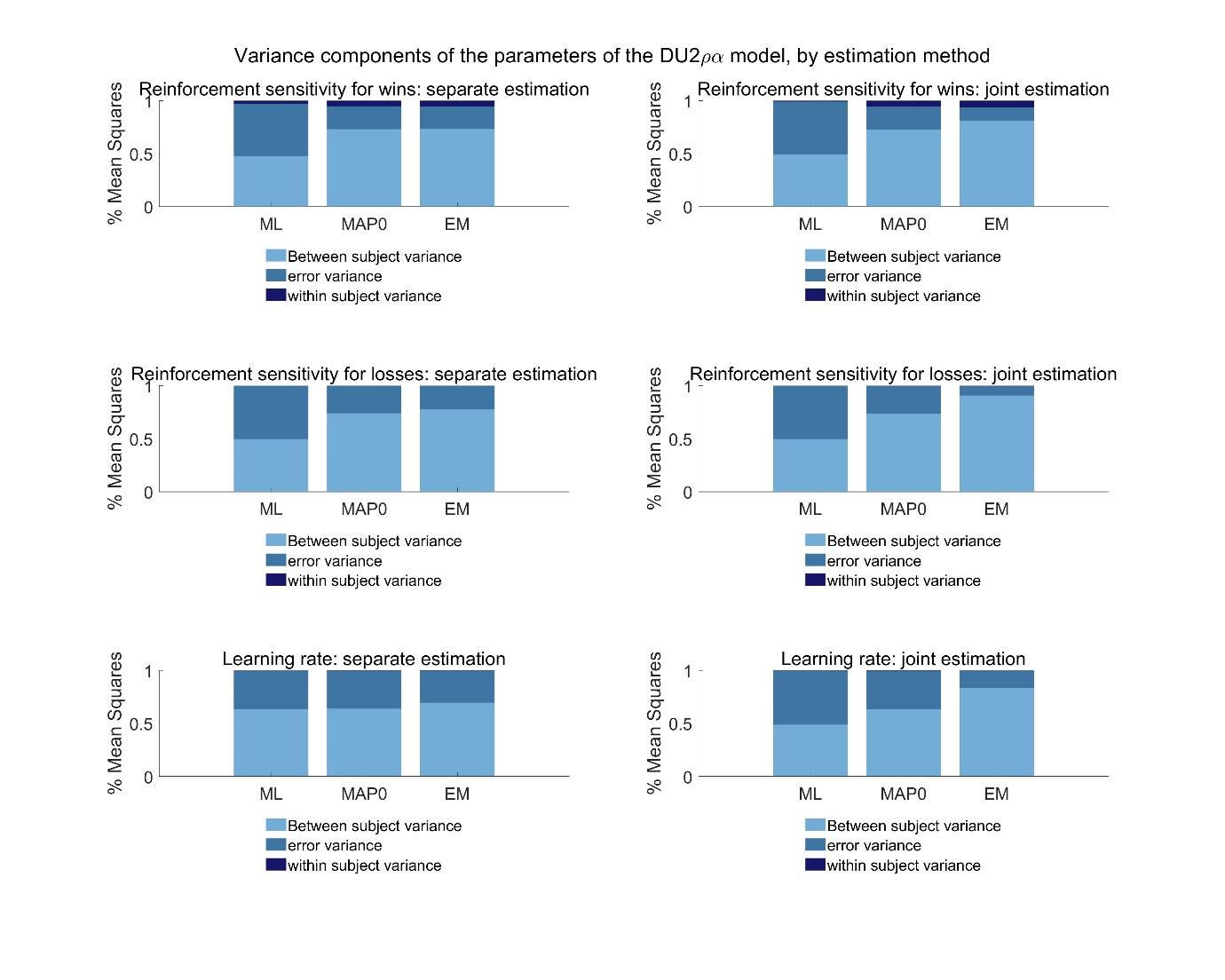


Figure 7: Within-subject, between-subject, and error variance proportions of parameter estimates from our best fitting computational model. Variance proportions for each parameter were calculated for each model fitting approach, namely maximum likelihood (ML), and maximum a posteriori estimation without (MAP0) and with (EM) informative priors for parameters, modelling sessions separately and jointly.


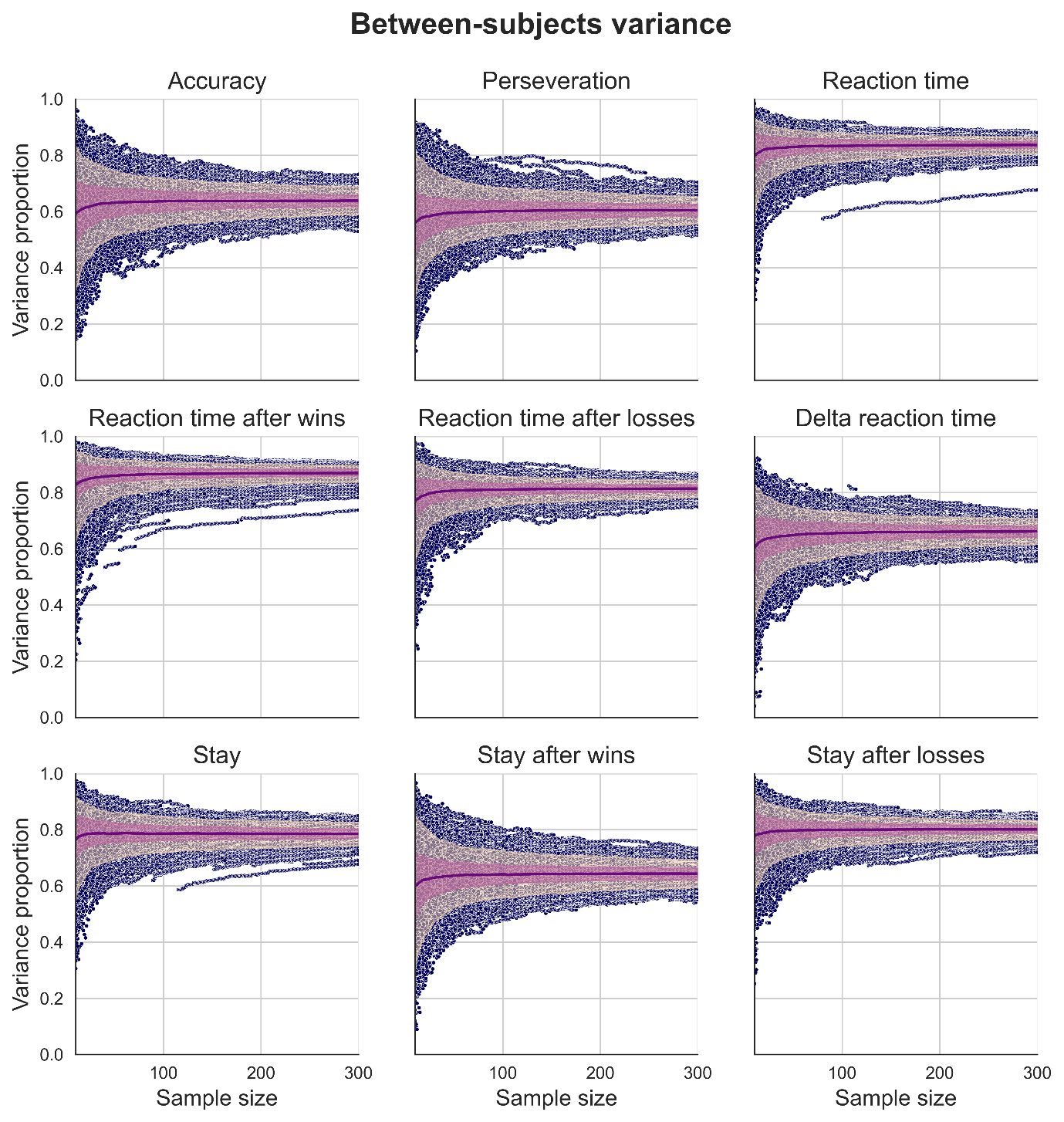


Figure 8: Distributions of between-subject variance proportions for simulated measures of behavioural performance generated using our regression-based approach. These data are generated using behavioural measures estimated using the simple means. Overlaid on individual datapoints (blue), is the mean proportion of variance for each sample size (purple), 90^th^ interpercentile range (dark pink) and interquartile range (light pink).


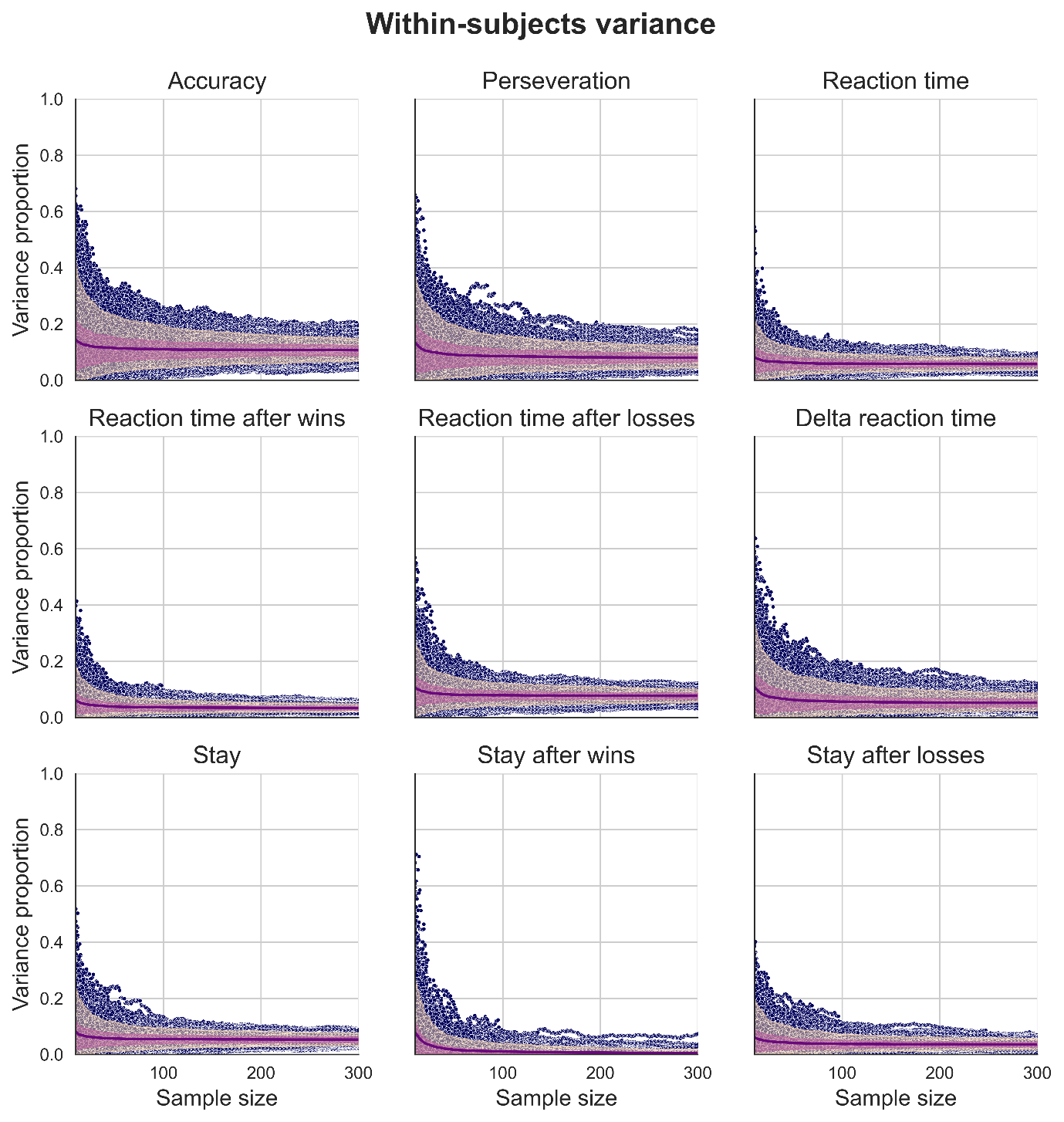


Figure 9: Distributions of within-subject variance proportions for simulated measures of behavioural performance generated using our regression-based approach. These data are generated using behavioural measures estimated using the simple means. Overlaid on individual datapoints (blue), is the mean proportion of variance for each sample size (purple), 90^th^ interpercentile range (dark pink) and interquartile range (light pink).


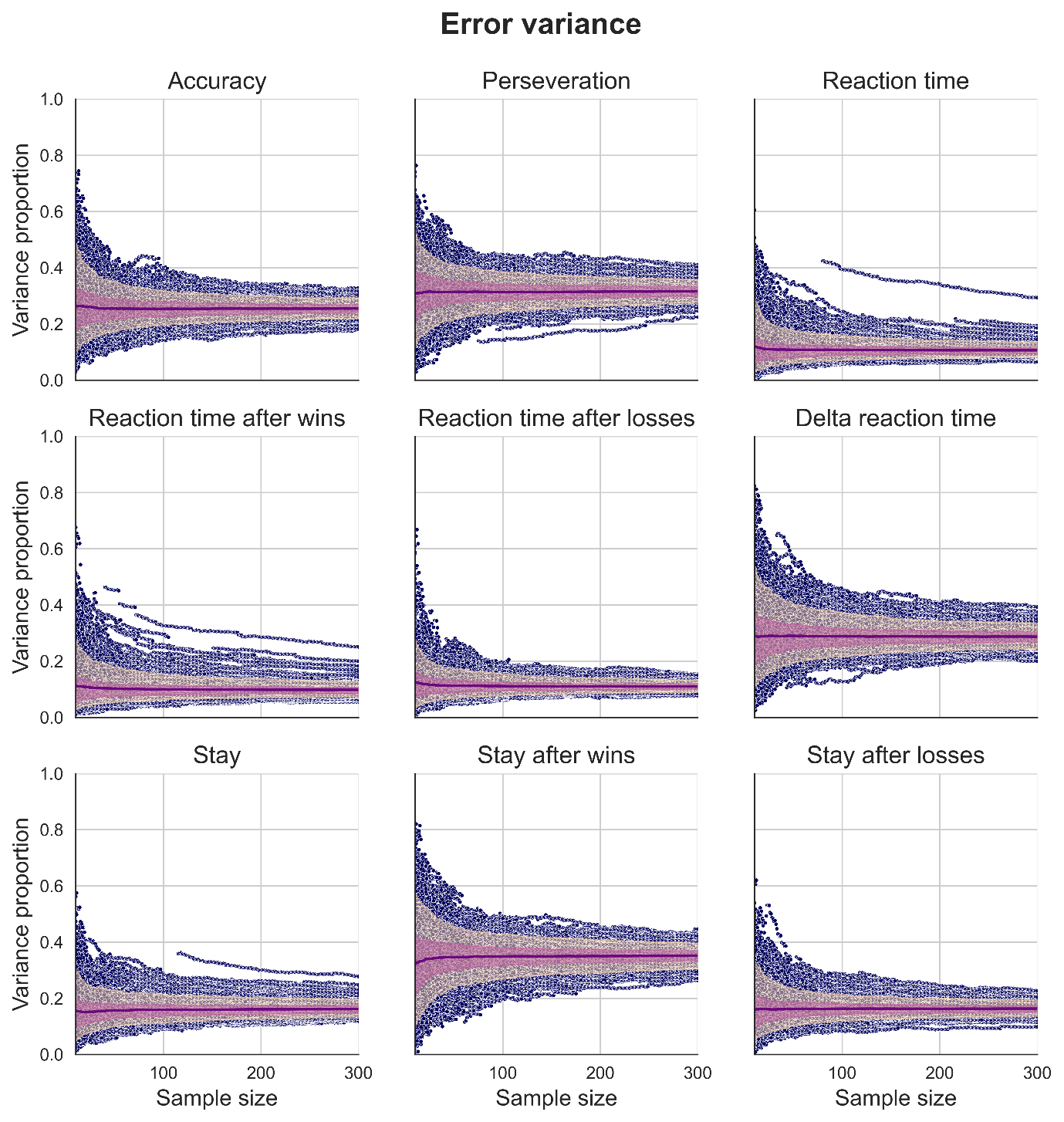


Figure 10: Distributions of error variance proportions for simulated measures of behavioural performance generated using our regression-based approach. These data are generated using behavioural measures estimated using the simple means. Overlaid on individual datapoints (blue), is the mean proportion of variance for each sample size (purple), 90^th^ interpercentile range (dark pink) and interquartile range (light pink).


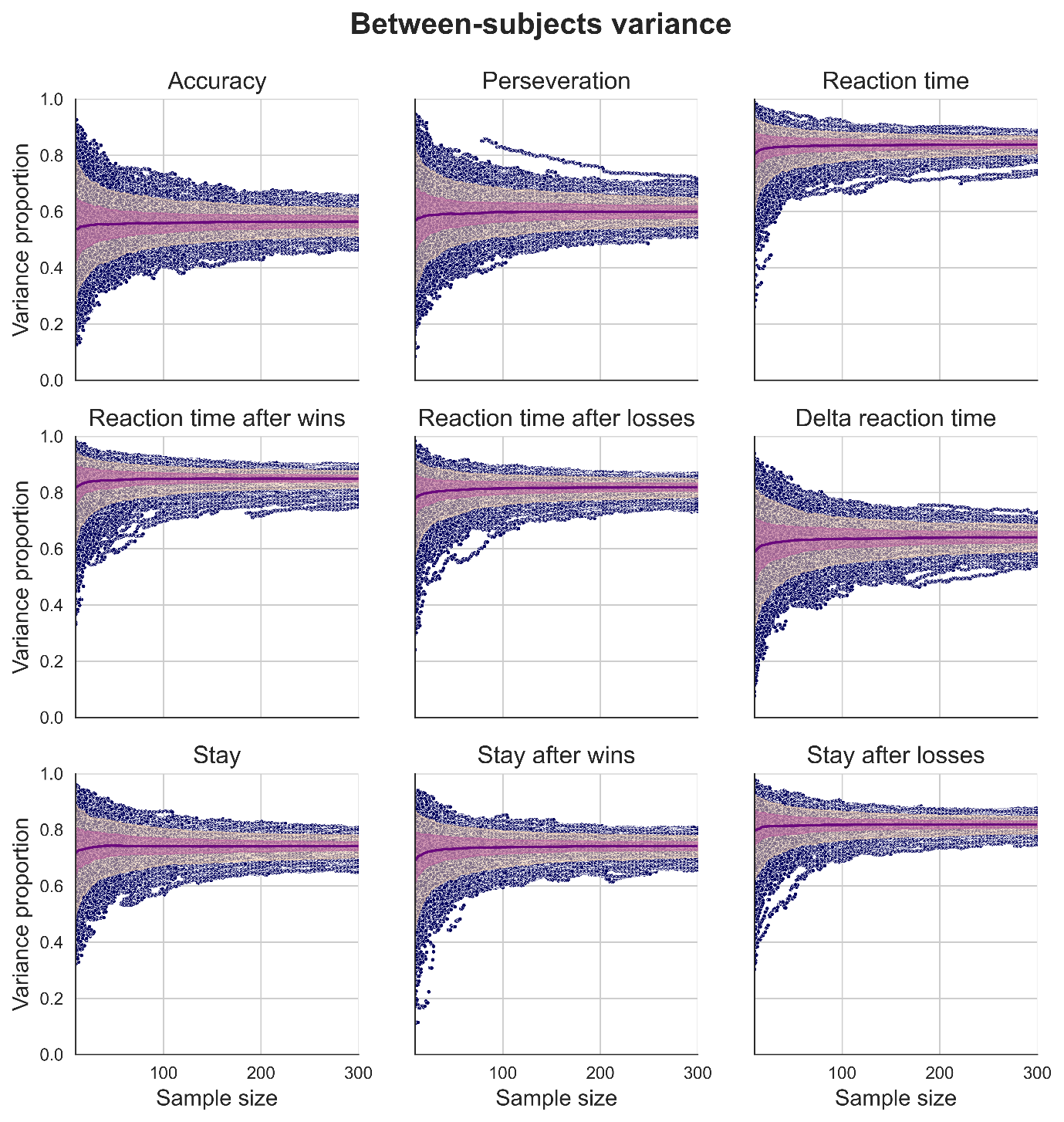


Figure 11: Distributions of between-subject variance proportions for simulated measures of behavioural performance generated using our regression-based approach. These data are generated using behavioural measures estimated using the “separate” regression models, which involved using separate regression models to estimate effects for each session. Overlaid on individual datapoints (blue), is the mean proportion of variance for each sample size (purple), 90th interpercentile range (dark pink) and interquartile range (light pink).


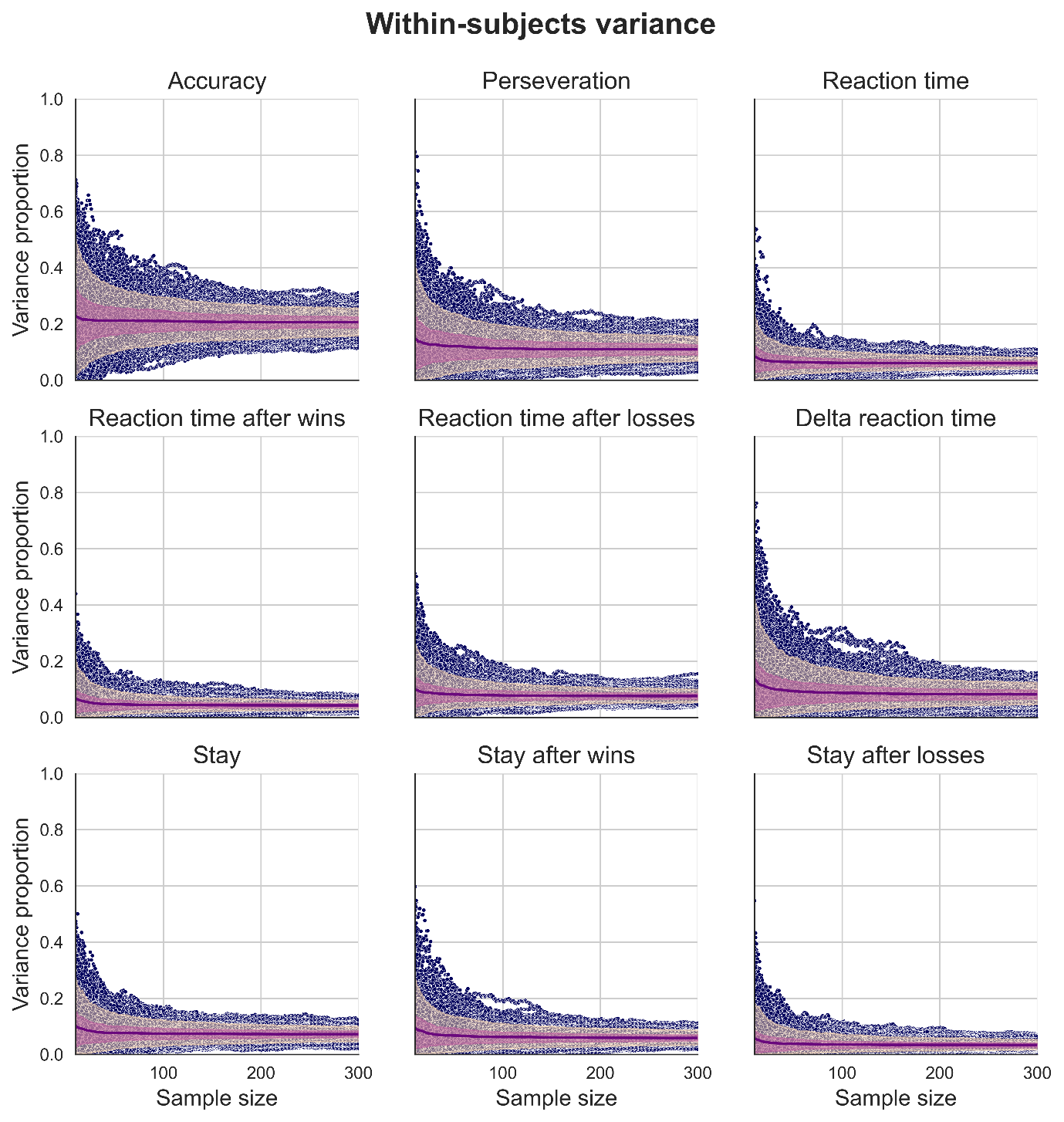


Figure 12: Distributions of within-subject variance proportions for simulated measures of behavioural performance generated using our regression-based approach. These data are generated using behavioural measures estimated using the “separate” regression models, which involved using separate regression models to estimate effects for each session. Overlaid on individual datapoints (blue), is the mean proportion of variance for each sample size (purple), 90th interpercentile range (dark pink) and interquartile range (light pink).


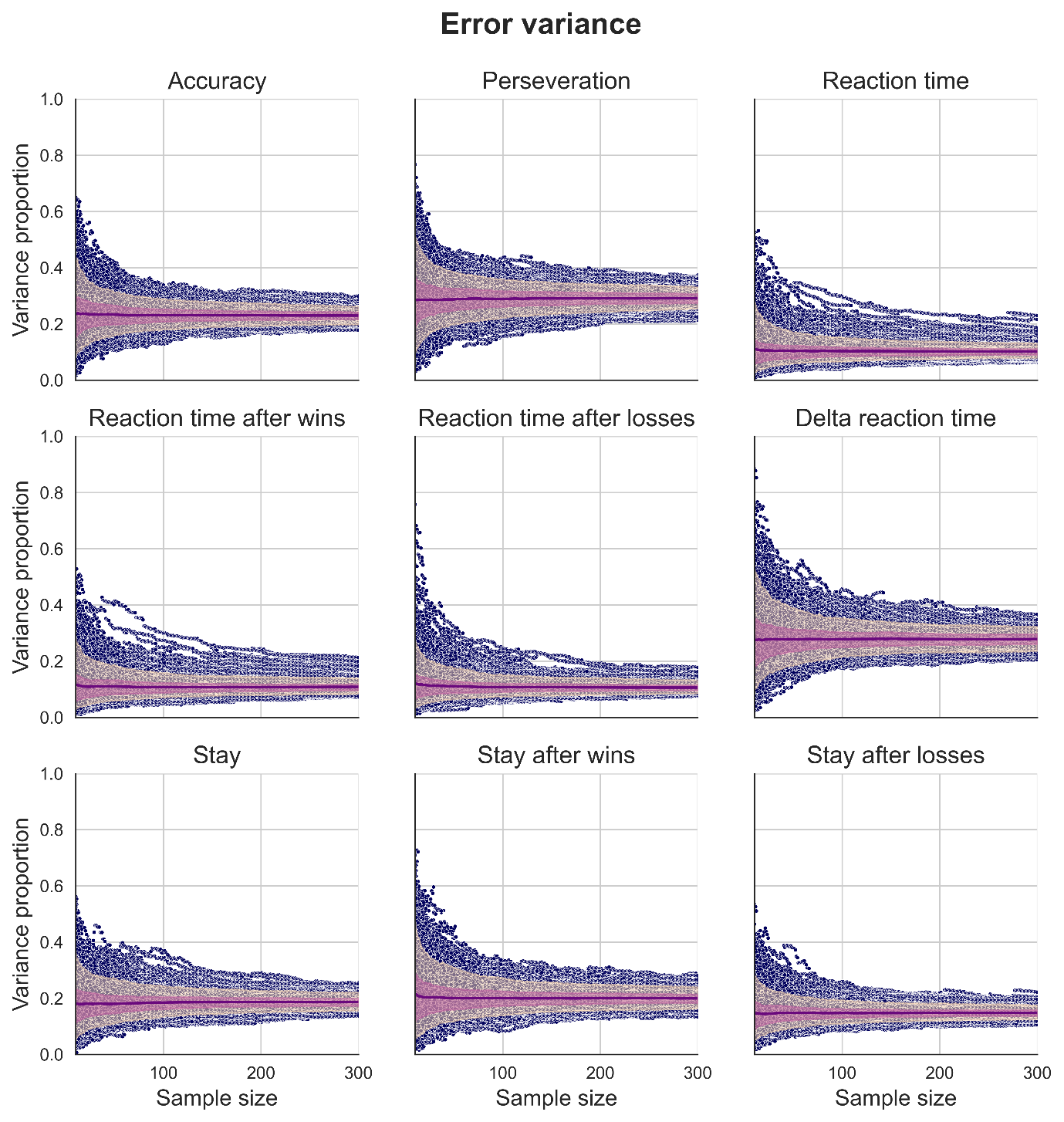


Figure 13: Distributions of error variance proportions for simulated measures of behavioural performance generated using our regression-based approach. These data are generated using behavioural measures estimated using the “separate” regression models, which involved using separate regression models to estimate effects for each session. Overlaid on individual datapoints (blue), is the mean proportion of variance for each sample size (purple), 90th interpercentile range (dark pink) and interquartile range (light pink).


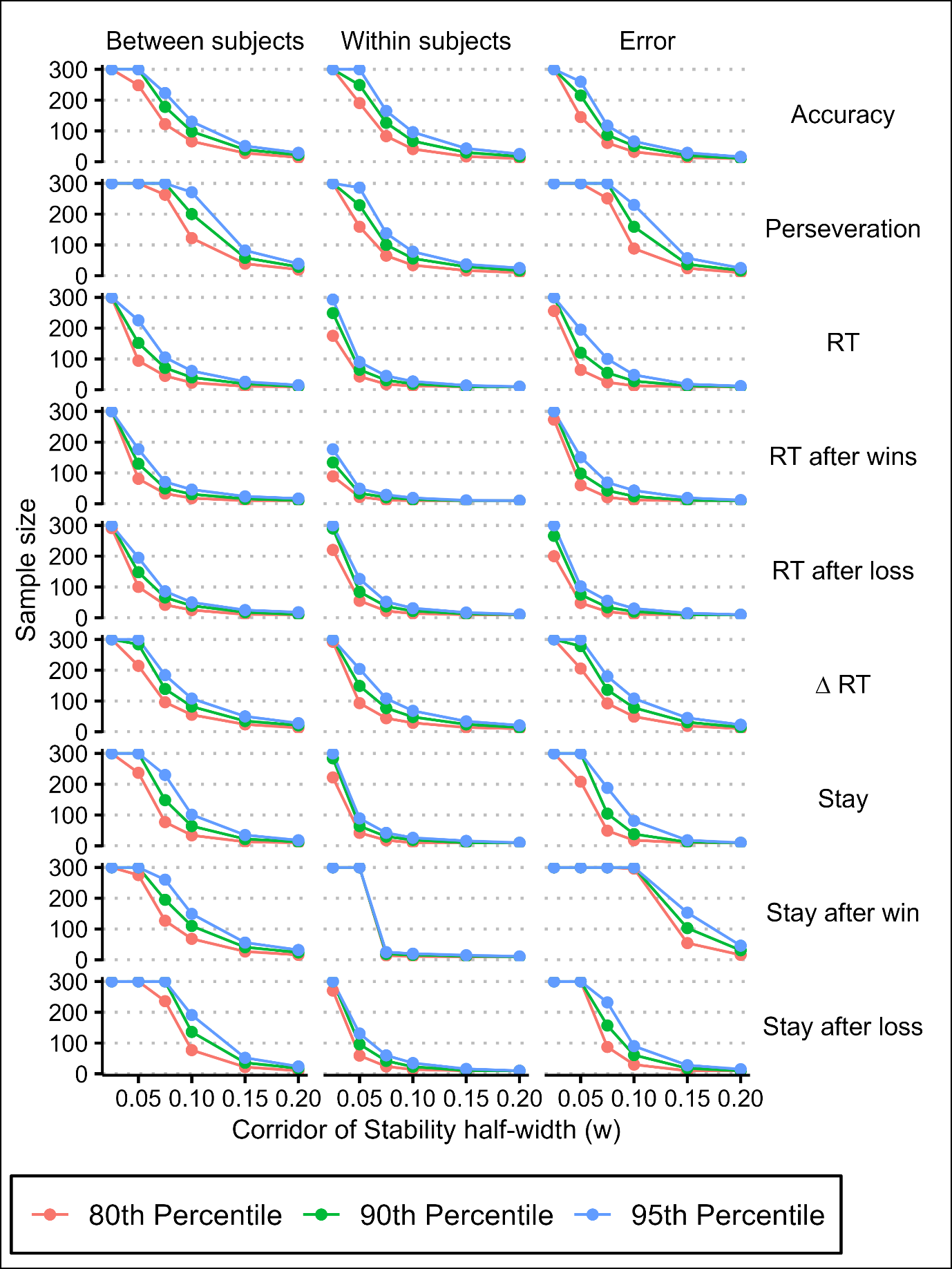


Figure 14: Critical Point of Stability of variance component estimates for synthetic behavioural measures across a range of Corridor of Stability half-width values. These plots are generated from synthetic data generated using the distributions of simple means.


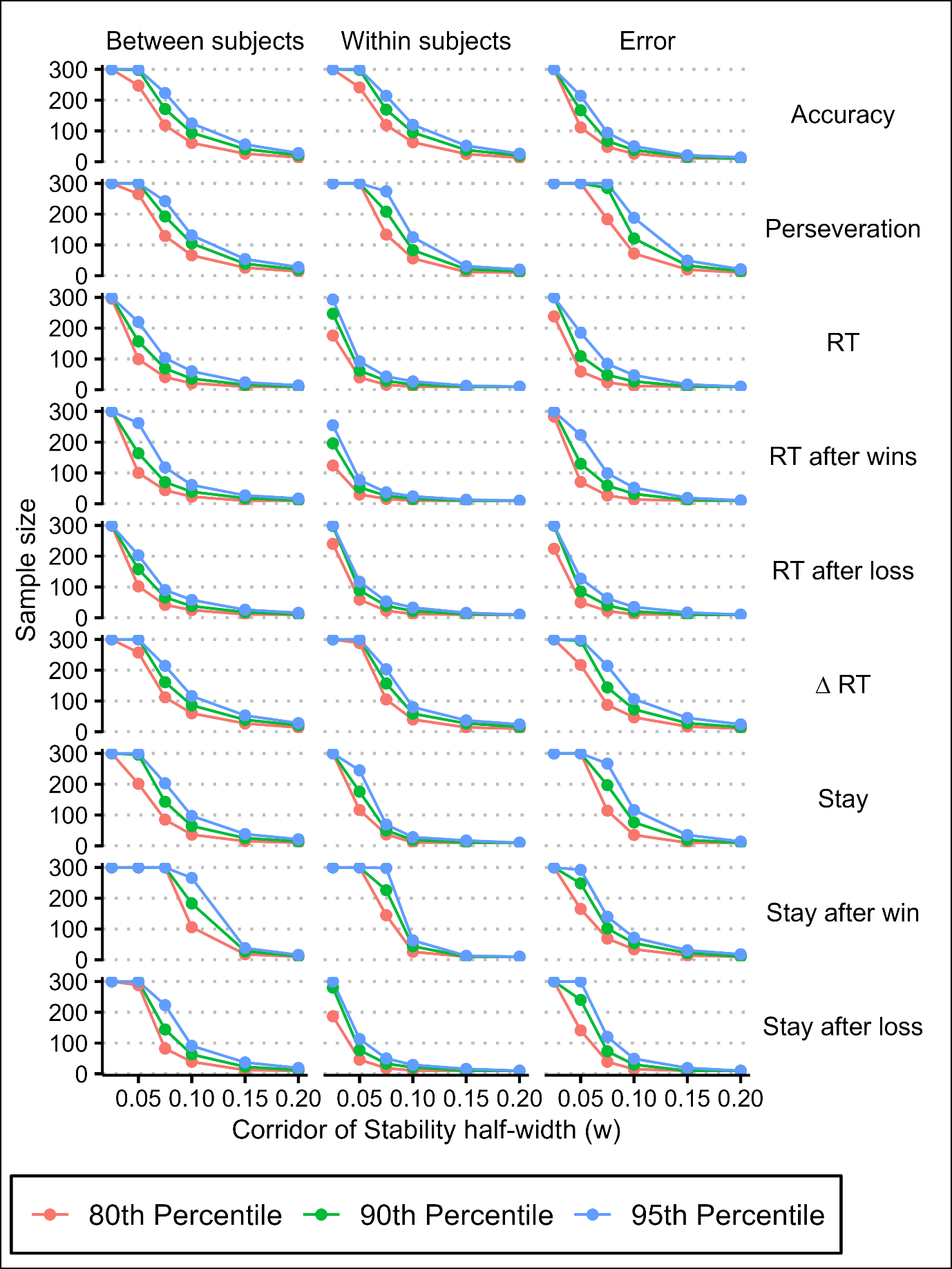


Figure 15: Critical Point of Stability of variance component estimates for synthetic behavioural measures across a range of Corridor of Stability half-width values. These plots are generated from synthetic data generated using the distributions of behavioural measures from the “separate” regression approach, which used separate regression models for each session.


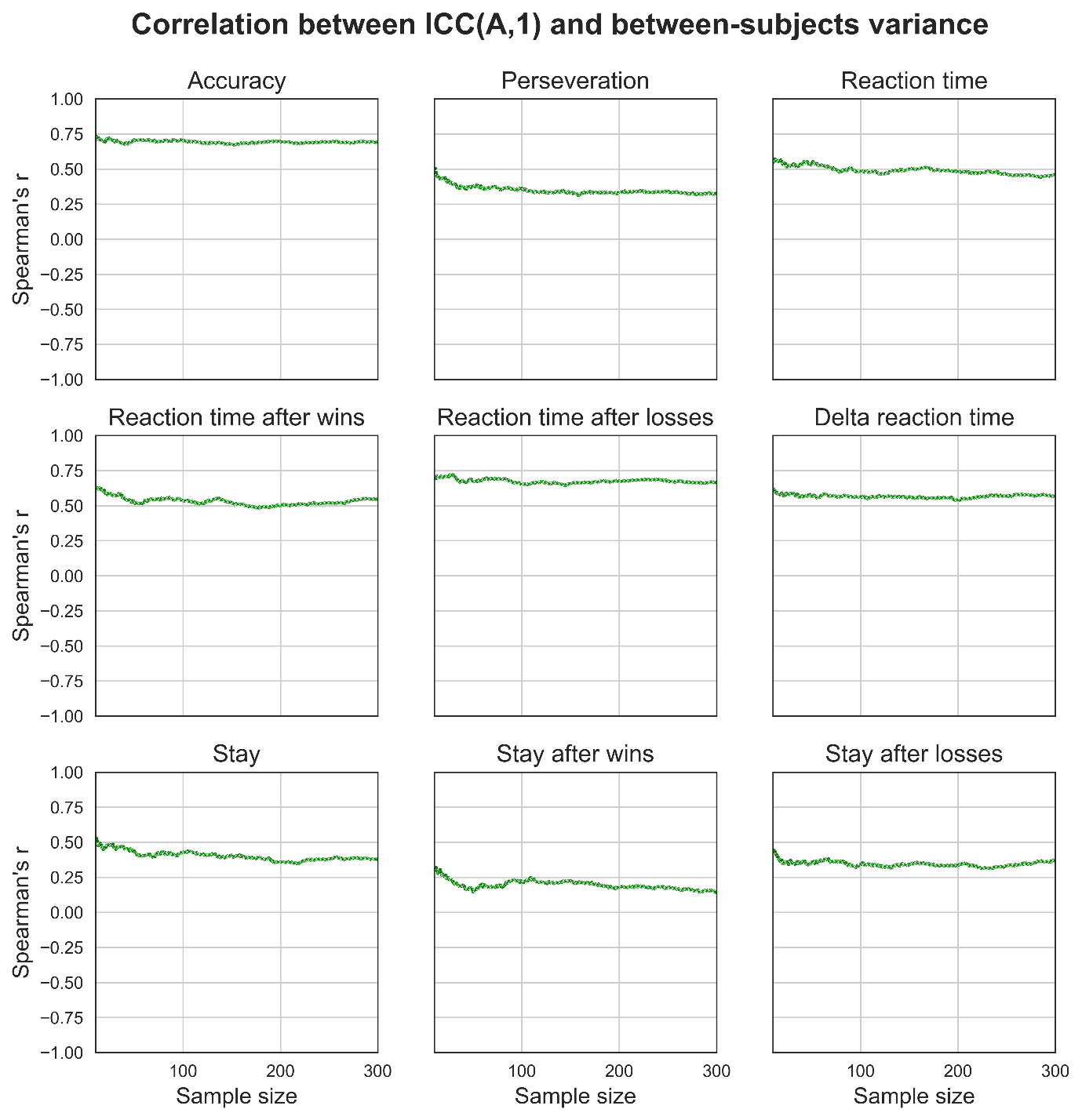


Figure 16: Sample size effects on the association between ICC coefficients and variance component estimates for behavioural measures. For each behavioural measure and at each sample size, we took the set of 1,000 simulated datasets and calculated correlation coefficients to measure the strength of the association between each dataset’s respective ICC(A,1) and variance component estimates. The point estimate for the correlation coefficient, and its statistical significance (coloured green for significant, red for non-significant; Bonferroni corrected) are then plotted. Overall, between-subjects variance was strongly positively correlated with ICC(A,1). These plots are generated from synthetic data generated using the distributions of simple means.


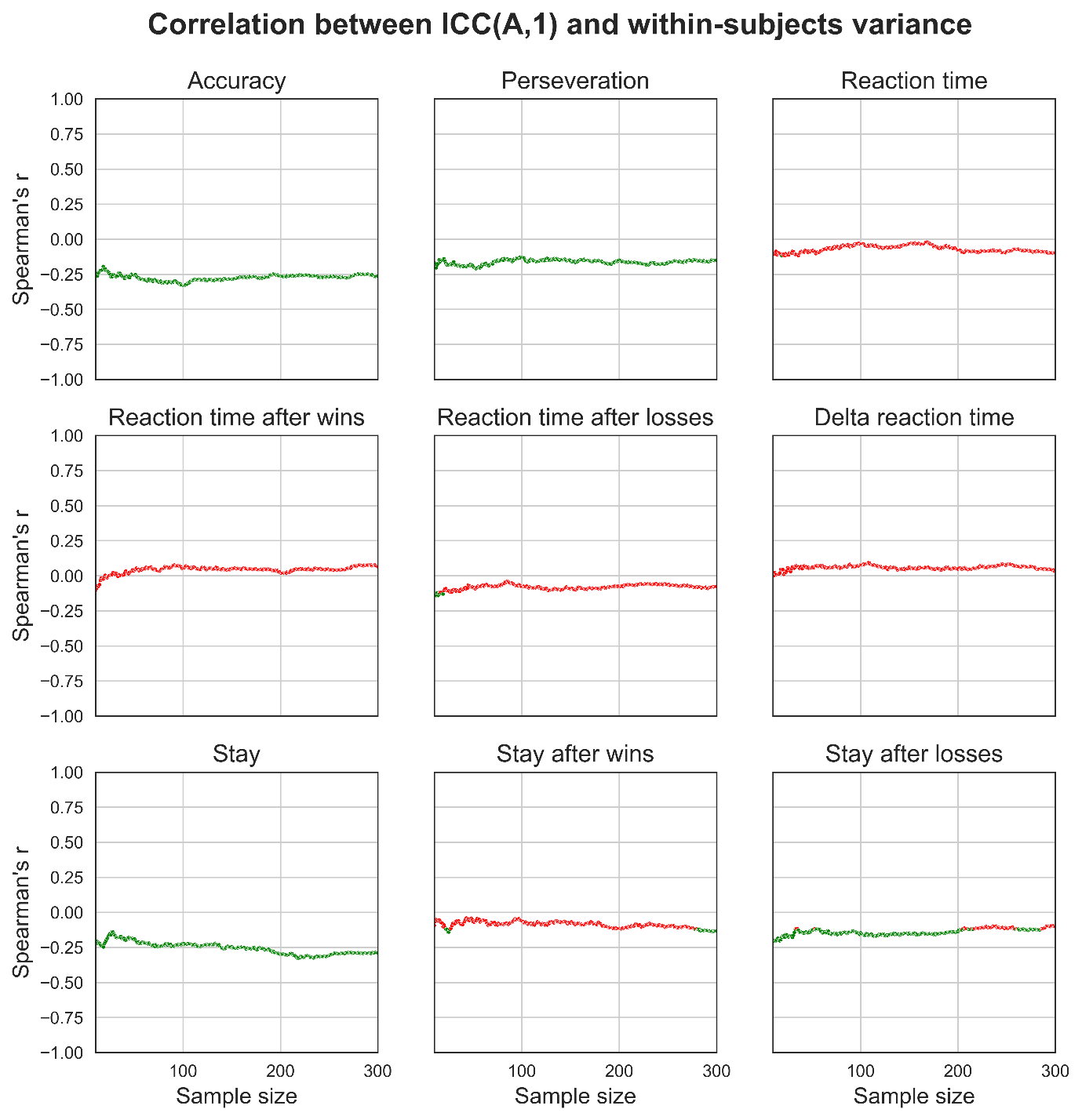


Figure 17: Sample size effects on the association between ICC coefficients and variance component estimates for behavioural measures. For each behavioural measure and at each sample size, we took the set of 1,000 simulated datasets and calculated correlation coefficients to measure the strength of the association between each dataset’s respective ICC(A,1) and variance component estimates. The point estimate for the correlation coefficient, and its statistical significance (coloured green for significant, red for non-significant; Bonferroni corrected) are then plotted. Overall, within-subjects variance was weakly or not correlated with ICC(A,1). These plots are generated from synthetic data generated using the distributions of simple means.


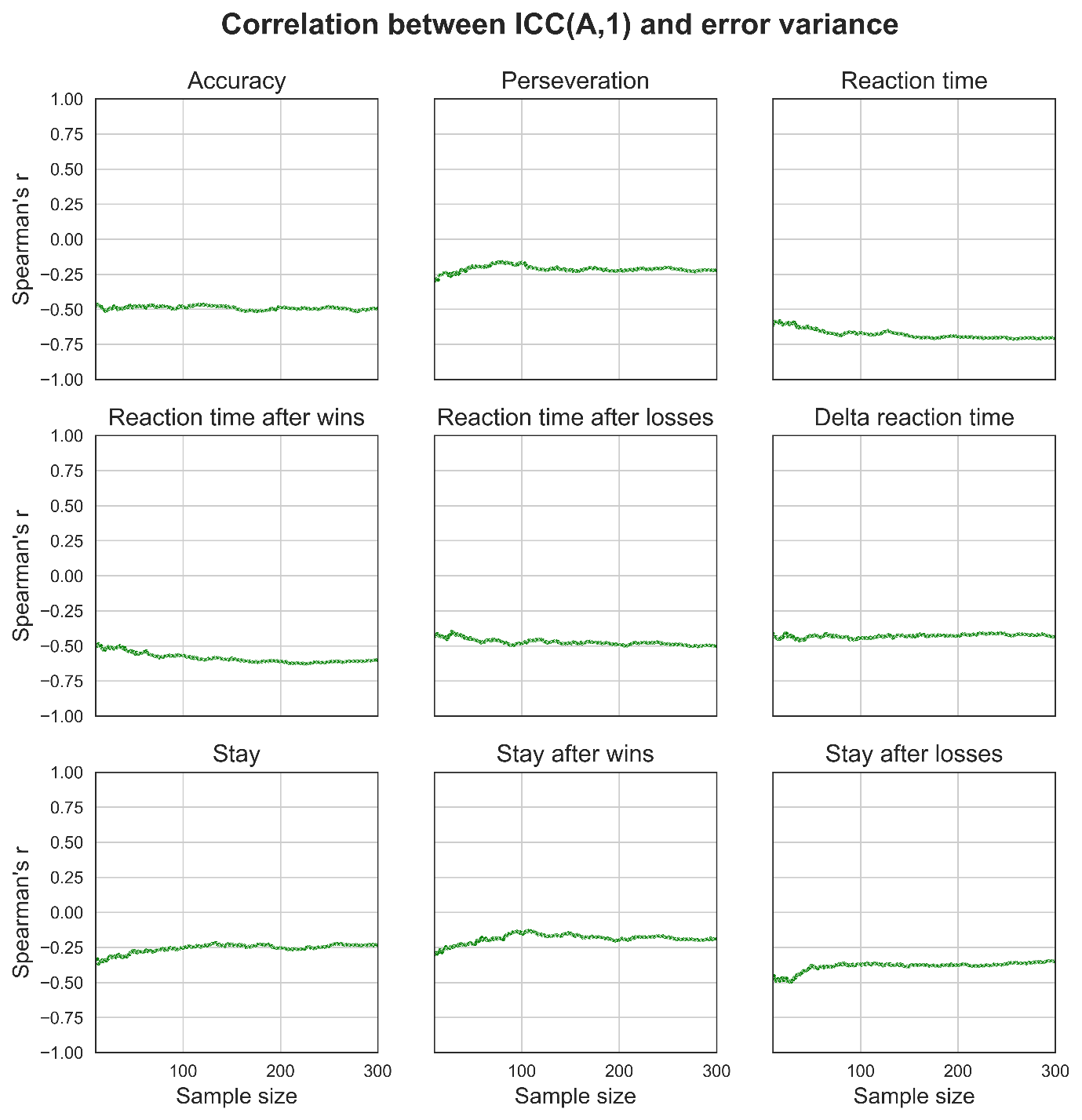


Figure 18: Sample size effects on the association between ICC coefficients and variance component estimates for behavioural measures. For each behavioural measure and at each sample size, we took the set of 1,000 simulated datasets and calculated correlation coefficients to measure the strength of the association between each dataset’s respective ICC(A,1) and variance component estimates. The point estimate for the correlation coefficient, and its statistical significance (coloured green for significant, red for non-significant; Bonferroni corrected) are then plotted. Overall, error variance was strongly negatively correlated with ICC(A,1). These plots are generated from synthetic data generated using the distributions of simple means.


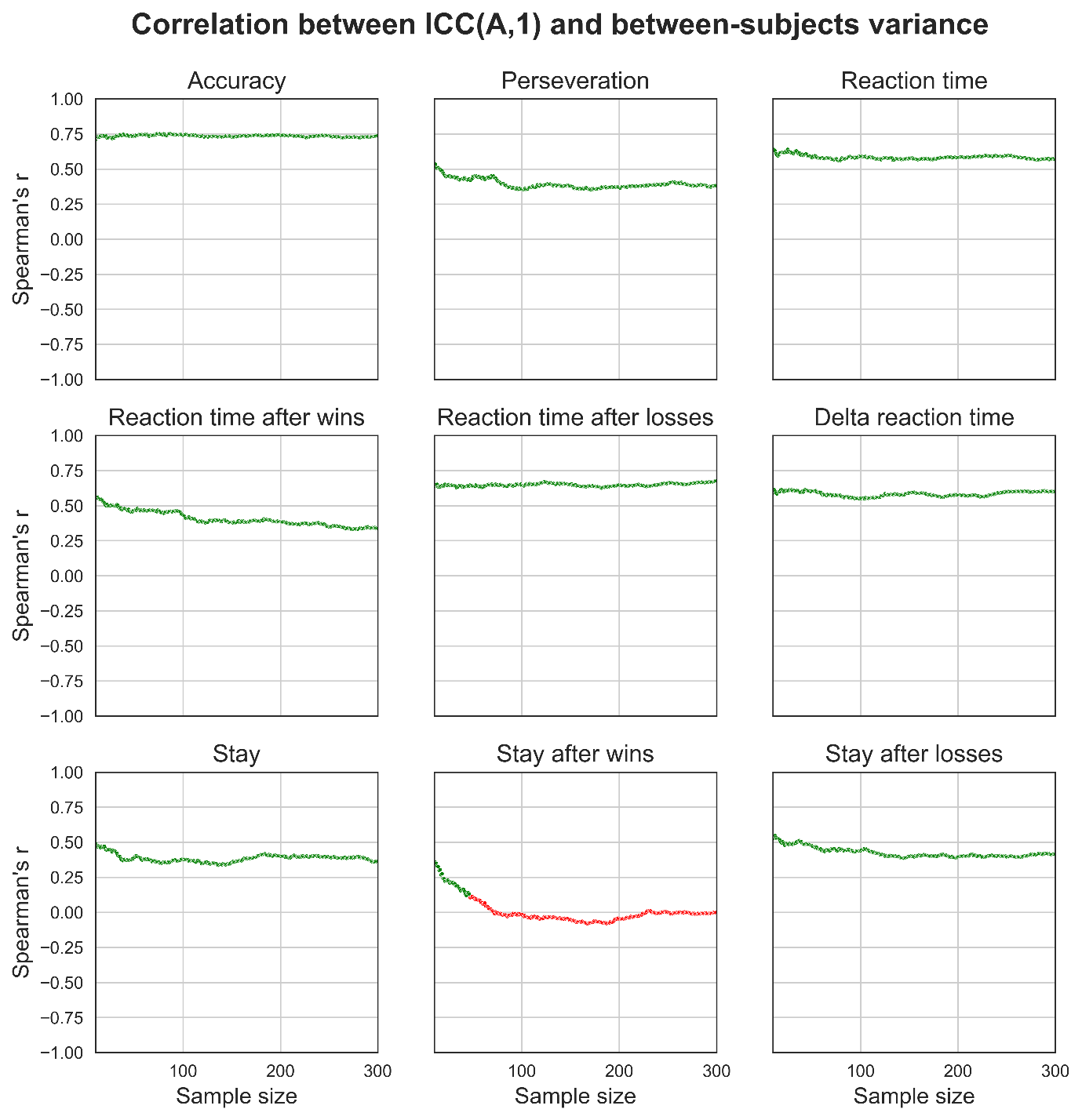


Figure 19: Sample size effects on the association between ICC coefficients and variance component estimates for behavioural measures. For each behavioural measure and at each sample size, we took the set of 1,000 simulated datasets and calculated correlation coefficients to measure the strength of the association between each dataset’s respective ICC(A,1) and variance component estimates. The point estimate for the correlation coefficient, and its statistical significance (coloured green for significant, red for non-significant; Bonferroni corrected) are then plotted. Overall, between-subjects variance was strongly positively correlated with ICC(A,1). These plots are generated from synthetic data generated using the distributions of behavioural measures from the “separate” regression approach, which used separate regression models for each session.


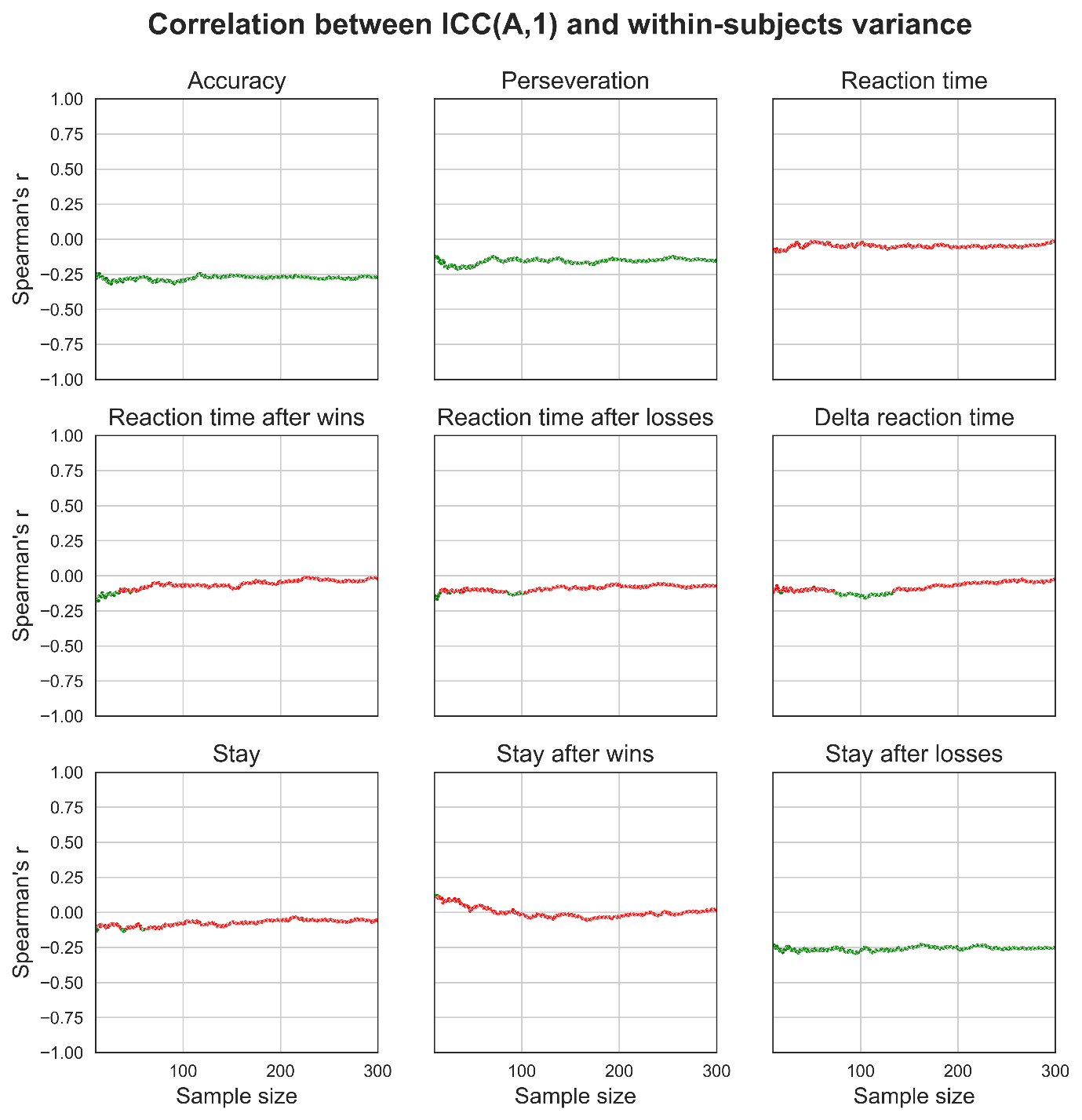


Figure 20: Sample size effects on the association between ICC coefficients and variance component estimates for behavioural measures. For each behavioural measure and at each sample size, we took the set of 1,000 simulated datasets and calculated correlation coefficients to measure the strength of the association between each dataset’s respective ICC(A,1) and variance component estimates. The point estimate for the correlation coefficient, and its statistical significance (coloured green for significant, red for non-significant; Bonferroni corrected) are then plotted. Overall, within-subjects variance was weakly or not correlated with ICC(A,1). These plots are generated from synthetic data generated using the distributions of behavioural measures from the “separate” regression approach, which used separate regression models for each session.


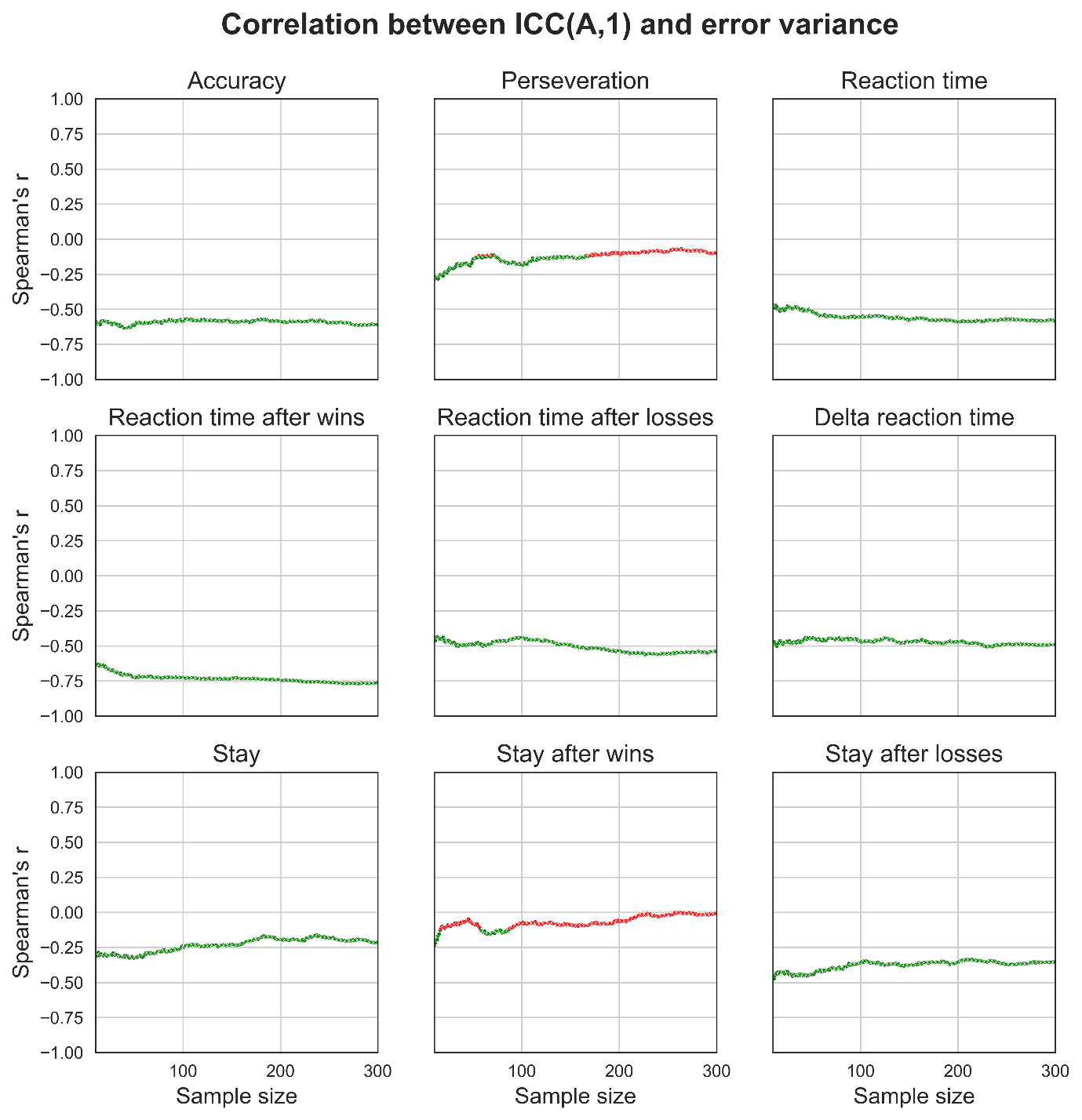


Figure 21: Sample size effects on the association between ICC coefficients and variance component estimates for behavioural measures. For each behavioural measure and at each sample size, we took the set of 1,000 simulated datasets and calculated correlation coefficients to measure the strength of the association between each dataset’s respective ICC(A,1) and variance component estimates. The point estimate for the correlation coefficient, and its statistical significance (coloured green for significant, red for non-significant; Bonferroni corrected) are then plotted. Overall, error variance was strongly negatively correlated with ICC(A,1). These plots are generated from synthetic data generated using the distributions of behavioural measures from the “separate” regression approach, which used separate regression models for each session.


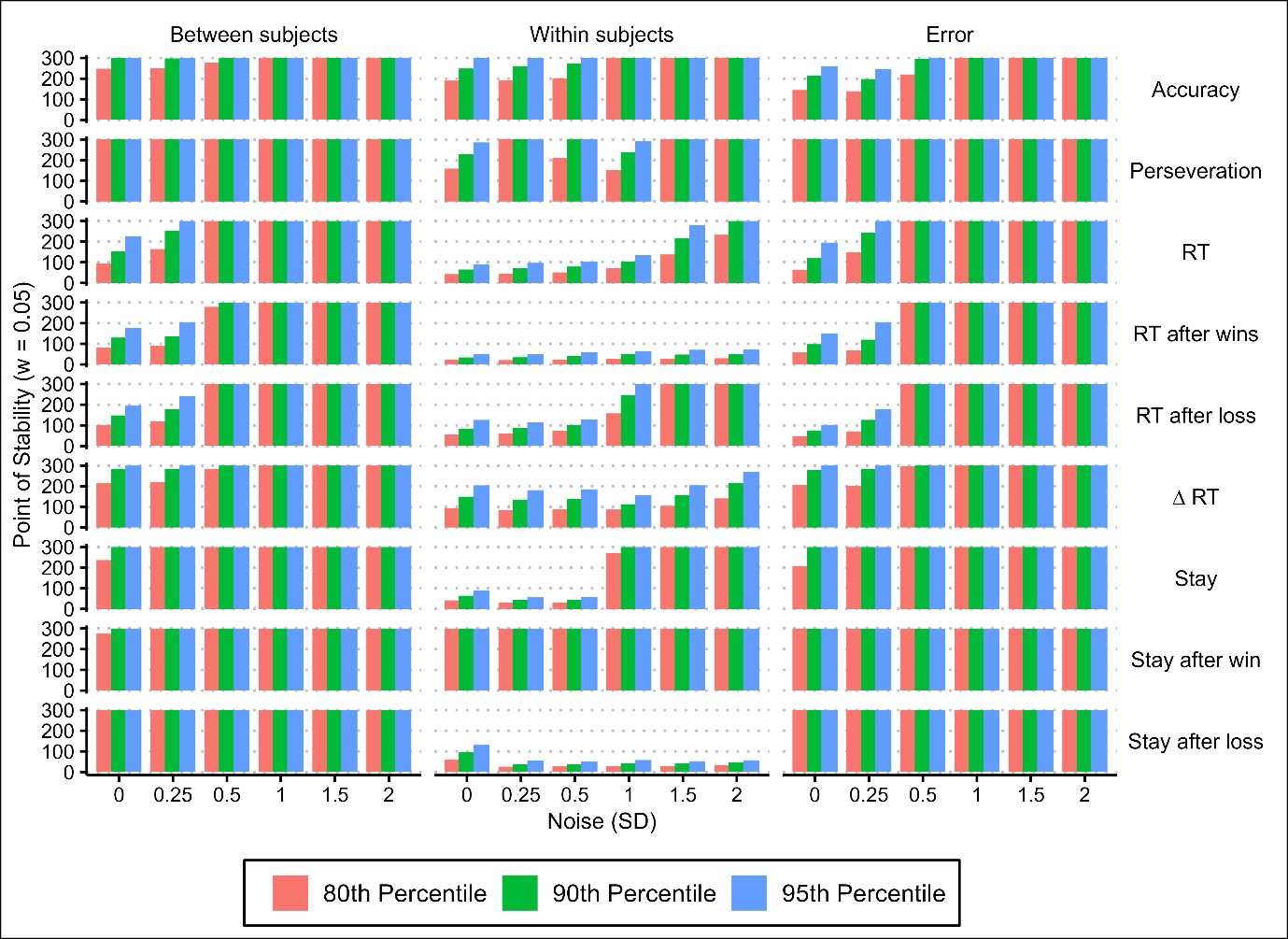


Figure 22: Noise effects on Point of Stability calculations for synthetically generated data. As the amount of noise added to synthesised behavioural and computational measures of task performance, a monotonic change in the Point of Stability for variance components is observed. For the majority of variance components, increasing amounts of simulated noise causes variance components to not reach a Point of Stability before the largest sample size is reached, meaning variance component estimates remain unstable. These plots are generated from synthetic data generated using the distributions of simple means.


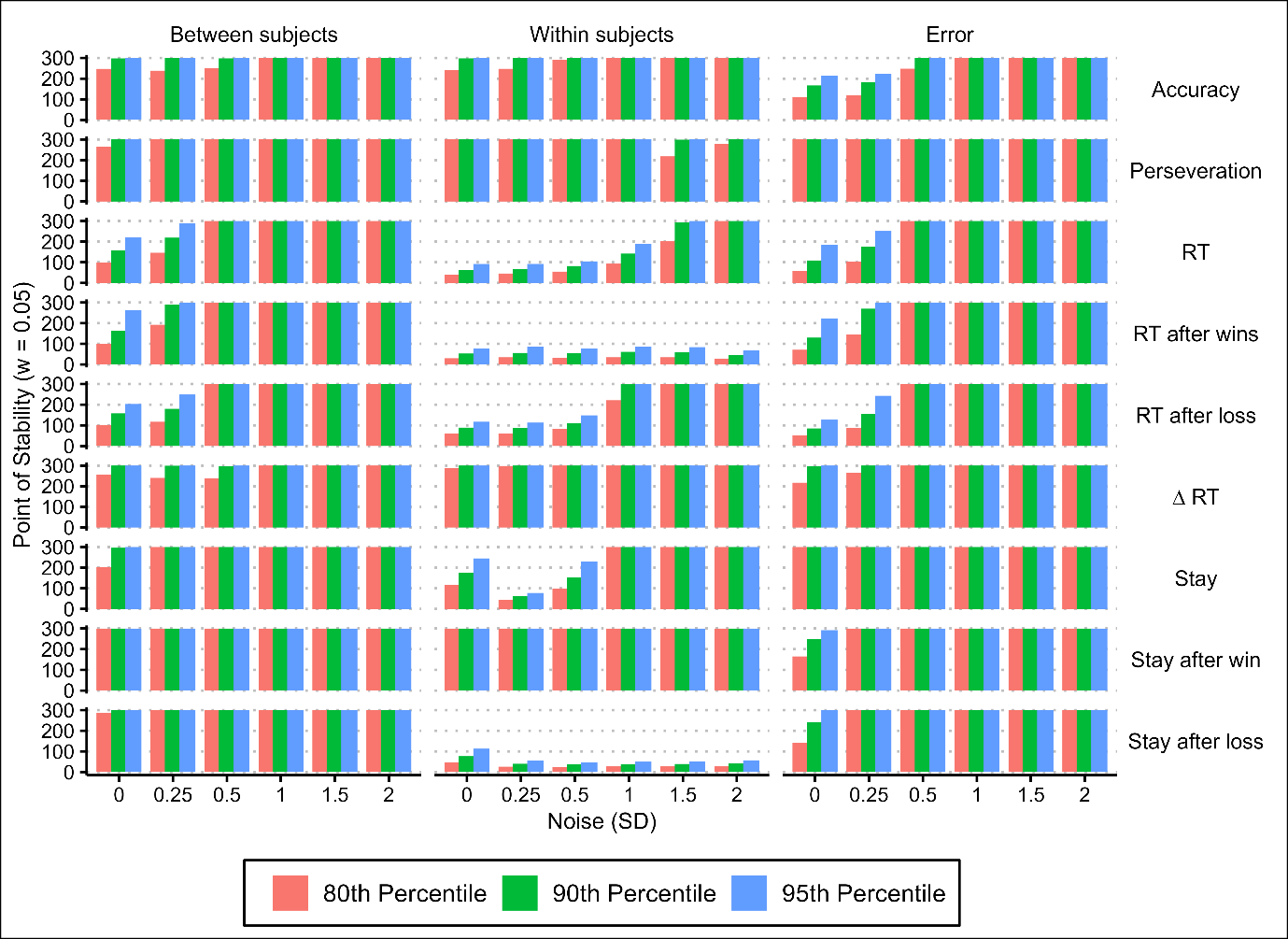


Figure 23: Noise effects on Point of Stability calculations for synthetically generated data. As the amount of noise added to synthesised behavioural and computational measures of task performance, a monotonic change in the Point of Stability for variance components is observed. For the majority of variance components, increasing amounts of simulated noise causes variance components to not reach a Point of Stability before the largest sample size is reached, meaning variance component estimates remain unstable. These plots are generated from synthetic data generated using the distributions of behavioural measures from the “separate” regression approach, which used separate regression models for each session.

# References

Bartko, J. J. (1966). The Intraclass Correlation Coefficient as a Measure of Reliability. *Psychological Reports*, *19*(1), 3–11. https://doi.org/10.2466/pr0.1966.19.1.3

Carleton, R. N., Norton, M. A. P. J., & Asmundson, G. J. G. (2007). Fearing the unknown: A short version of the Intolerance of Uncertainty Scale. *Journal of Anxiety Disorders*, *21*(1), 105–117. https://doi.org/10.1016/j.janxdis.2006.03.014

Cicchetti, D. V. (1994). Guidelines, criteria, and rules of thumb for evaluating normed and standardized assessment instruments in psychology. *Psychological Assessment*, *6*, 284–290. https://doi.org/10.1037/1040-3590.6.4.284

Gell, M., Eickhoff, S. B., Omidvarnia, A., Küppers, V., Patil, K. R., Satterthwaite, T. D., Müller, V. I., & Langner, R. (2023). *The Burden of Reliability: How Measurement Noise Limits Brain-Behaviour Predictions* (p. 2023.02.09.527898). bioRxiv. https://doi.org/10.1101/2023.02.09.527898

Huang, J. L., Bowling, N. A., Liu, M., & Li, Y. (2015). Detecting Insufficient Effort Responding with an Infrequency Scale: Evaluating Validity and Participant Reactions. *Journal of Business and Psychology*, *30*(2), 299–311. https://doi.org/10.1007/s10869-014-9357-6

McGraw, K. O., & Wong, S. P. (1996). Forming inferences about some intraclass correlation coefficients. *Psychological Methods*, *1*(1), 30. https://doi.org/10.1037/1082-989X.1.1.30

Niv, Y., Edlund, J. A., Dayan, P., & O’Doherty, J. P. (2012). Neural Prediction Errors Reveal a Risk-Sensitive Reinforcement-Learning Process in the Human Brain. *Journal of Neuroscience*, *32*(2), 551–562. https://doi.org/10.1523/JNEUROSCI.5498-10.2012

Waltmann, M., Schlagenhauf, F., & Deserno, L. (2022). Sufficient reliability of the behavioral and computational readouts of a probabilistic reversal learning task. *Behavior Research Methods*. https://doi.org/10.3758/s13428-021-01739-7

Zorowitz, S., Solis, J., Niv, Y., & Bennett, D. (2023). Inattentive responding can induce spurious associations between task behaviour and symptom measures. *Nature Human Behaviour*, *7*(10), 1667–1681. https://doi.org/10.1038/s41562-023-01640-7
